# Identification of HIF1A as a therapeutic target during SARS-CoV-2–associated lung injury

**DOI:** 10.1172/jci.insight.191463

**Published:** 2025-06-17

**Authors:** Bentley Bobrow, Samuel D. Luber, Paul Potnuru, Katherine Figarella, Jieun Kim, Yanyu Wang, In Hyuk Bang, David Robinson, Paulina B. Sergot, Steven K. Burke, Tingting Mills, Constanza de Dios, Robert Suchting, George W. Williams, Adit A. Ginde, Yafen Liang, Hongfang Liu, Charles Green, Marie-Francoise Doursout, Alparslan Turan, Daniel I. Sessler, Xiaoyi Yuan, Holger K. Eltzschig

**Affiliations:** 1 Department of Emergency Medicine, McGovern Medical School,; 2 Department of Anesthesiology, Critical Care and Pain Medicine, McGovern Medical School, and; 3 Center for Outcomes Research, Department of Anesthesiology, Critical Care and Pain Medicine, McGovern Medical School, University of Texas Health Science Center at Houston, Houston, Texas, USA.; 4 Akebia Therapeutics, Inc., Cambridge, Massachusetts, USA.; 5 Department of Biochemistry, McGovern Medical School, and; 6 Department of Psychiatry and Behavioral Sciences, McGovern Medical School, University of Texas Health Science Center at Houston, Houston, Texas, USA.; 7 Department of Emergency Medicine, University of Colorado School of Medicine, Aurora, Colorado, USA.; 8 Department of Health Data Science and Artificial Intelligence, D. Bradley McWilliams School of Biomedical Informatics, and; 9 Institute for Clinical Research and Learning Health Care, Department of Pediatrics, UTHealth Houston; Houston, Texas, USA.

**Keywords:** Inflammation, Therapeutics, Drug therapy, Hypoxia

## Abstract

Hypoxia-inducible factors (HIFs) promote lung protection and pathogen eradication during acute lung injury. We, therefore, tested the theory that pharmacologic stabilization of HIFs dampens lung injury during SARS-CoV-2 pneumonia. Initial studies in murine SARS-CoV-2 models showed improved outcomes after treatment with the FDA-approved HIF stabilizer vadadustat. Subsequent studies in genetic models implicated alveolus-expressed *Hif1a* in mediating lung protection. Therefore, we performed a randomized, double-blinded, multicenter phase II trial in patients admitted for SARS-CoV-2 infection and concomitant hypoxia (SpO_2_ ≤ 94%). Patients (*n* = 448) were randomized to oral vadadustat (900 mg/day) or placebo for up to 14 days. Safety events were similar between the 2 groups. Vadadustat treatment induced surrogate HIF target genes. The primary outcome of severe lung injury requiring high oxygen support on day 14 occurred in 43 patients in the vadadustat group and 53 patients in the placebo group (estimated probability, 13.3% vs. 16.9%). Among patients with baseline fraction of inspired oxygen of 80% or higher (*n* = 106), the estimated probability of the primary outcome was 12.1% (vadadustat) versus 79.1% (placebo), indicating an even greater benefit in patients with more severe baseline hypoxia. HIF1A is a likely therapeutic target during SARS-CoV-2–associated lung injury. Robust clinical trials of HIF stabilizers during pathogen-associated lung injury are warranted.

## Abstract 2

Murine studies of SARS-CoV-2-associated lung injury identify HIF1A as a therapeutic target. A subsequent clinical trial suggests a likelihood of benefit for HIF stabilizer treatment in patients.

## Introduction

Most patients with viral pneumonia experience only mild-to-moderate symptoms (1). But during the COVID-19 pandemic, up to 15% of patients with SARS-CoV-2 infections proceeded to severe pneumonia, and 5% experienced life-threatening acute respiratory distress syndrome (ARDS) (2–5). Pharmacological treatments that dampen pulmonary inflammation and promote resolution of lung injury during viral pneumonia, including SARS-CoV-2 infections, would be clinically meaningful (4, 6–8).

Hypoxia-inducible factors (HIFs) are a group of transcription factors discovered in the early 1990s (9), inducing expression of genes that promote adaptations to limited oxygen availability (10). For example, HIFs bind to the erythropoietin promoter and induce its expression during hypoxia (9, 11). Multiple genes are regulated by HIFs, including glycolytic pathway enzymes, growth factors, and genes that regulate innate and adaptive immunity along with cancer progression (12–14). HIFs dampen alveolar inflammation and pulmonary edema by optimizing carbohydrate metabolism in alveolar epithelial cells (15).

HIFs also contribute to the resolution of lung injury after an inflammatory insult (16, 17), which is considered an active process (18). HIFs are also crucial in pathogen defense. For example, HIF1A activation improves the bactericidal capacity of phagocytes (19), and HIF enhancement by a pharmacologic HIF stabilizer reduces mortality in mice infected with *Pseudomonas aeruginosa* (20). Furthermore, HIF stabilizer treatment reduces influenza A viral replication in vitro (21) and improves the outcomes of murine influenza A viral infection marked by faster weight recovery and improved survival (22). Finally, treatment with a HIF stabilizer reduces viral replication in human alveolar epithelial cells in vitro (23) and hamsters infected with SARS-CoV-2 in vivo (24).

Pharmacological HIF stabilizers inhibit HIF-prolyl hydroxylases (HIF-PHDs), a set of enzymes that target HIFs for proteasomal degradation (16, 18, 25). Two HIF stabilizers, daprodustat and vadadustat, are now FDA approved for treating anemia in patients with dialysis-dependent chronic kidney disease (26, 27). However, whether vadadustat is also effective for virus-associated lung injury remains unknown. We, therefore, tested the hypothesis that the HIF stabilizer vadadustat attenuates SARS-CoV-2–associated lung injury. Specifically, we conducted studies in mice and used the results to guide a randomized phase II trial to assess the efficacy and safety of vadadustat for treating acute SARS-CoV-2 lung injury.

## Results

### HIF stabilizer vadadustat provides lung protection during SARS-CoV-2 infection in mice

Previous experimental studies implicate HIF stabilizers in lung protection during ARDS or pathogen-associated lung injury (28). We thus initially pursued experimental studies using murine models to examine the functional role of the FDA-approved HIF stabilizer vadadustat during viral pneumonia with SARS-CoV-2. Specifically, we examined the effect of vadadustat treatment on HIF stabilization in the lungs of mice.

**Figure 1:**
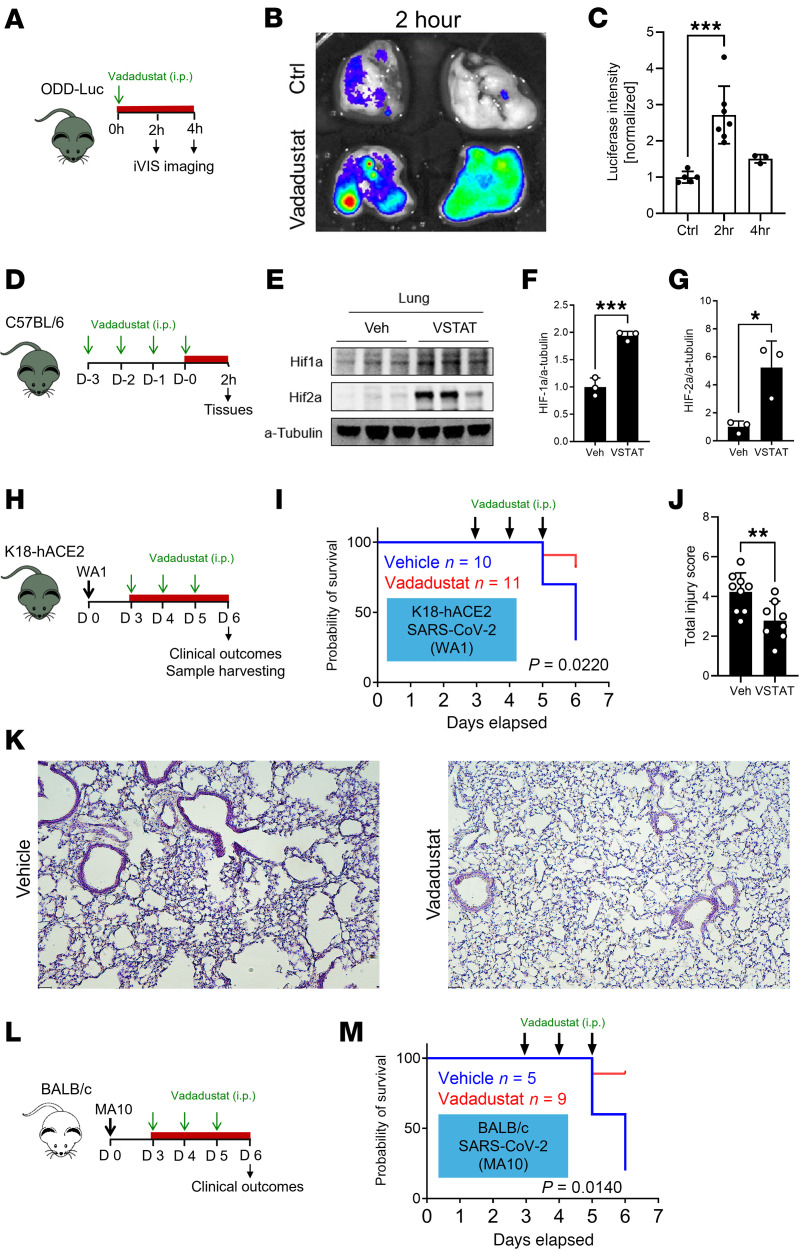
The HIF stabilizer vadadustat provided lung protection during SARS-CoV-2 infection in mice. (**A**) Schematic diagram of vadadustat treatment in HIF reporter ODD-luc mice. (**B**) Representative ex vivo bioluminescence imaging of luciferase activity using IVIS imager at 2 hours after vadadustat i.p. injection. (**C**) Quantification of bioluminescence intensity at 2 hours and 4 hours after vadadustat i.p. injection. Data are presented as mean ± SD. (**D**) Schematic diagram of vadadustat treatment in C57BL/6 mice. (**E**) Hif1A or Hif2A immunoblotting was performed on protein isolated from whole lung tissue after treatment with vadadustat. Each column represents 1 animal. (**F** and **G**) Quantification of Hif1a and Hif2a protein after treatment with vadadustat for 3 days. Data are presented as mean ± SD. (**H**) Schematic diagram of WA1 infection (280 PFU) in K18-hACE2 mice treated with vadadustat. (**I**) Kaplan-Meier plots of K18-hACE2 mice with vehicle or vadadustat treatment. *P* values were calculated with the Mantel-Cox test. (**J**) Blinded histological injury scores of the lungs were quantified as described in the Methods. Data are represented as mean ± SEM. (**K**) Representative H&E staining images of lung tissue from vehicle- and vadadustat-treated mice. Scale bars: 50 μm. (**L**) Schematic diagram of MA10 infection (200 PFU) in BALB/c mice treated with vadadustat. (**M**) Kaplan-Meier plots of BALB/c mice with vehicle or vadadustat treatment. *P* values were calculated with the Mantel-Cox test. *P < 0.05, **P < 0.01, ***P < 0.001 by 1-way ANOVA with Dunnett’s multiple-comparison test (**C**) or 2-tailed Student’s *t* test (**F**, **G**, and **J**).

We used previously described HIF reporter mice with transgenic expression of the human HIFA oxygen-dependent degradation domain linked to a luciferase reporter (ODD-luc mice) (29). There were significant increases in luciferase activity in the lungs of ODD-luc mice 2 hours after intraperitoneal (i.p.) injection of vadadustat (Figure 1, A–C). Luciferase activity in the lungs decreased 4 hours after the i.p. injection, consistent with the reported half-life of vadadustat (30, 31). To identify which isoform of HIFA is stabilized by vadadustat treatment, we performed Western blot analyses of lung tissues 2 hours after injection and observed significant stabilization of HIF1A and HIF2A (Figure 1, D–G). To investigate the activation of HIF signaling, we assessed the expression level of several HIF target genes and observed an increase in erythropoietin in the lung after vadadustat treatment (Supplemental Figure 1; supplemental material available online with this article; https://doi.org/10.1172/jci.insight.191463DS1).

To assess the potential therapeutic role of vadadustat treatment during SARS-CoV-2 pneumonia, we used mice with transgenic overexpression of human angiotensin-converting enzyme 2 (K18-hACE2 mice) (32, 33) and infected them with 280 PFU of SARS-CoV-2 (variant WA1) (34). To investigate vadadustat as a treatment for SARS-CoV-2 infection, instead of a preventative strategy, daily vadadustat treatment began 3 days after the infection (Figure 1H). Mice treated with vadadustat showed significant survival improvement compared with mice given vehicle (Figure 1I). Mortality in the vehicle group was 80%, whereas 18% of mice died in the treatment group (*P* = 0.022). We next investigated the impact of vadadustat treatment on lung injury. Pathological lung injury scores were attenuated in mice treated with vadadustat after SARS-CoV-2 infection (Figure 1, J and K, and Supplemental Figure 2). To further investigate the HIF stabilizer vadadustat in recovery from SARS-CoV-2 infection, we carried out a vadadustat treatment study in murine-adapted virus (MA10) (35) in BALB/c mice. Similarly, mortality in the vadadustat-treated group was significantly lower compared with the vehicle-treated group, with animals starting recovery on day 5 after infection (Figure 1, L and M, and Supplemental Figure 3). Taken together, our results indicate that treatment with the FDA-approved HIF stabilizer vadadustat stabilizes HIFs in the lungs of mice and improves clinically meaningful outcomes of SARS-CoV-2 infections of mice in vivo.

### Epithelia-expressed Hif1a mediates lung protection during SARS-CoV-2 pneumonia

Both dominant isoforms of HIFA (HIF1A and HIF2A) have been implicated in lung protection during ARDS (31, 36, 37). For example, there appear to be functional roles for HIF1A in optimizing alveolar-epithelial metabolism during ARDS (15). There is also a functional role of HIF2A in promoting endothelial barrier function and dampening lung edema during noninfectious ARDS (37). However, the roles of HIF1A and HIF2A during viral pneumonia, such as SARS-CoV-2 infections, remain unclear.

**Figure 2:**
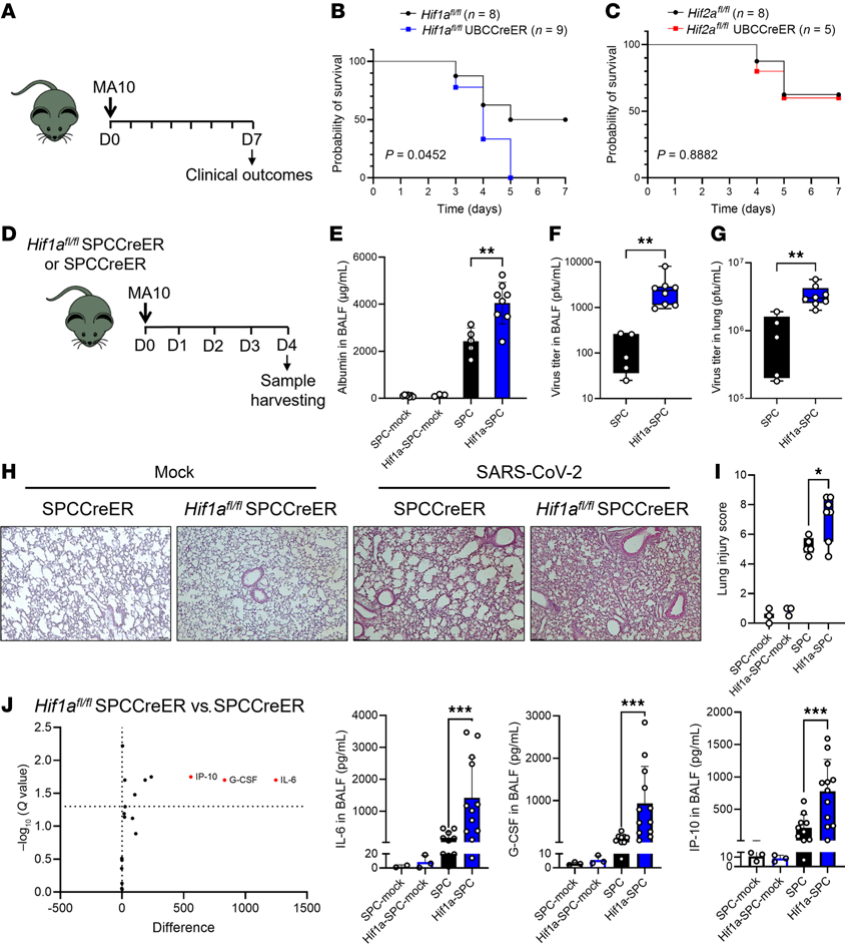
Selective role of HIF1A-mediated lung protection during SARS-CoV-2 infection. (**A**) Mice were inoculated with 3 × 10^4^ PFU of the murine-adapted SARS-CoV-2 strain (MA10) via oropharyngeal aspiration, and clinical outcomes were monitored over 7 days. (**B** and **C**) The survival rate in SARS-CoV-2–infected mice with whole-body deletion of Hif1a (*Hif1a^fl/fl^* UBCCreER) or (**C**) Hif2a-deleted mice (*Hif2a^fl/fl^* UBCCreER) compared to their respective *Hif^fl/fl^* litter mates. *P* values were obtained using the Mantel-Cox test. (**D**) Mice with a specific deletion of Hif1a in alveolar epithelial cells (*Hif1a^fl/fl^* SPCCreER) and their Cre-inducible counterpart (SPCCreER) were infected with 3 × 10^3^ PFU of the MA10 strain via oropharyngeal aspiration or mock infected, monitored for clinical outcomes and euthanized on day 4 to harvest BALF and lung tissue. (**E**) Albumin concentration in BALF was measured by ELISA. Data are represented as mean ± SEM. Two-tailed Student’s *t* test. (**F** and **G**) Viral load in BALF and lung tissue was detected by plaque assay. Gaussian distribution was assayed using the Shapiro-Wilk test. Unpaired 2-tailed Student’s *t* test or Mann-Whitney *U* test was applied to parametric or nonparametric data, respectively. (**H**) The lungs of infected SPCCreER and *Hif1a^fl/fl^* SPCCreER mice 4 days after infection were collected, fixed, and paraffin embedded. H&E staining was performed, and images were taken at ×10 magnification (*n =* 5 or 8, respectively; representative images are shown). Scale bars: 200 μm. (**I**) The lung injury score was performed blindly. In the bar-and-whisker plots, the bounds of the boxes represent the 25%–75% interquartile range, the lines within the boxes represent the median, the whiskers represent data min/max, and there are no outlying values. Two-tailed Student’s *t* test. (**J**) Inflammatory molecules were measured using a multiplex array in the BALF from SPCCreER and *Hif1a^fl/fl^* SPCCreER SARS-CoV-2– or mock-infected mice. Volcano plot resulting from an unpaired 2-tailed Student’s *t* test with Welch’s correction comparing both groups. Molecules that were highly differentially secreted are emphasized in red. The column graphs represent individual results for IL-6, G-CSF, and IP-10 (*n =* 10–12). Unpaired 2-tailed Student’s *t* tests with Welch’s correction or Mann-Whitney *U* test was applied to parametric or nonparametric data. Normality was established using the Shapiro-Wilk test. *P < 0.05, **P < 0.01, ***P < 0.001.

We, therefore, infected mice with induced global deletion of *Hif1a* (38) or *Hif2a* with SARS-CoV-2 (MA10, 3 × 10^4^ PFU) and tracked the disease progression for 7 days (Figure 2A). Both experimental groups were treated with tamoxifen for 5 days and rested for 7 days prior to infection. Interestingly, mortality was increased in mice with induced global deletion of HIF1A (*Hif1a^fl/fl^* UBCCreER) compared with control *Hif1a^fl/fl^* mice (Figure 2B). However, response rates were similar in *Hif2a^fl/fl^* UBCCreER and control mice (Figure 2C). HIF1A thus provides lung protection during murine SARS-CoV-2 infection, whereas HIF2A does not.

Since type II alveolar epithelial cells are the primary target for SARS-CoV-2 infection in the lungs (3), we examined lung inflammation and injury in mice with an inducible deletion of *Hif1a* specifically in alveolar epithelial cells (*Hif1a^fl/fl^* SPCCreER) (13) (Figure 2D). Four days after mock or SARS-CoV-2 infection, there was increased albumin leakage into bronchoalveolar lavage fluid (BALF) synonymous with pulmonary edema in the infection group, while baseline levels were similar between *Hif1a^fl/fl^* SPCCreER and SPCCreER control mice (Figure 2E). Previous studies suggest that HIFs promote pathogen clearance (39, 40), including during viral pneumonia (23, 24). We, therefore, performed plaque assays on BALF and lung tissue to assess the SARS-CoV-2 viral load. Indeed, the viral load in *Hif1a^fl/fl^* SPCCreER was significantly higher than in SPCCreER mice in both BALF samples and the lung tissue, suggesting that HIF1A is essential for controlling viral replication (Figure 2, F and G).

Consistent with higher viral loads, histologic lung injury was exaggerated in *Hif1a^fl/fl^* SPCCreER mice, while baseline levels were similar between *Hif1a^fl/fl^* SPCCreER and SPCCreER control mice (Figure 2, H and I). Several studies suggest an association between higher levels of inflammatory cytokines/chemokines and more severe disease outcomes during COVID-19 (41, 42). To assess lung inflammation, we used a multiplex platform (43, 44) to simultaneously assess the level of key cytokine/chemokine/growth factors in the BALF. IL-6, G-CSF, and IFN-γ–inducible protein-10 (IP-10) were significantly increased in *Hif1a^fl/fl^* SPCCreER mice during SARS-CoV-2 infection, with no major changes in the mock-infected groups (Figure 2J), suggesting heightened pulmonary inflammation, which is consistent with observed exaggerated injury.

### Vadadustat activates HIFs and improves outcomes in SARS-CoV-2–associated lung injury in patients

Inspired by preclinical findings suggesting a protective role of HIF activation in SARS-CoV-2 infection, we conducted a randomized, double-blind, placebo-controlled phase II trial in hypoxemic (oxygen saturation [SpO_2_] ≤ 94%) patients hospitalized for SARS-CoV-2 infection at 5 US sites (ClinicalTrials.gov NCT04478071). Between August 2020 and March 2022, we randomized 227 patients to placebo and 221 patients to receive vadadustat 900 mg daily for up to 14 days.

**Figure 3:**
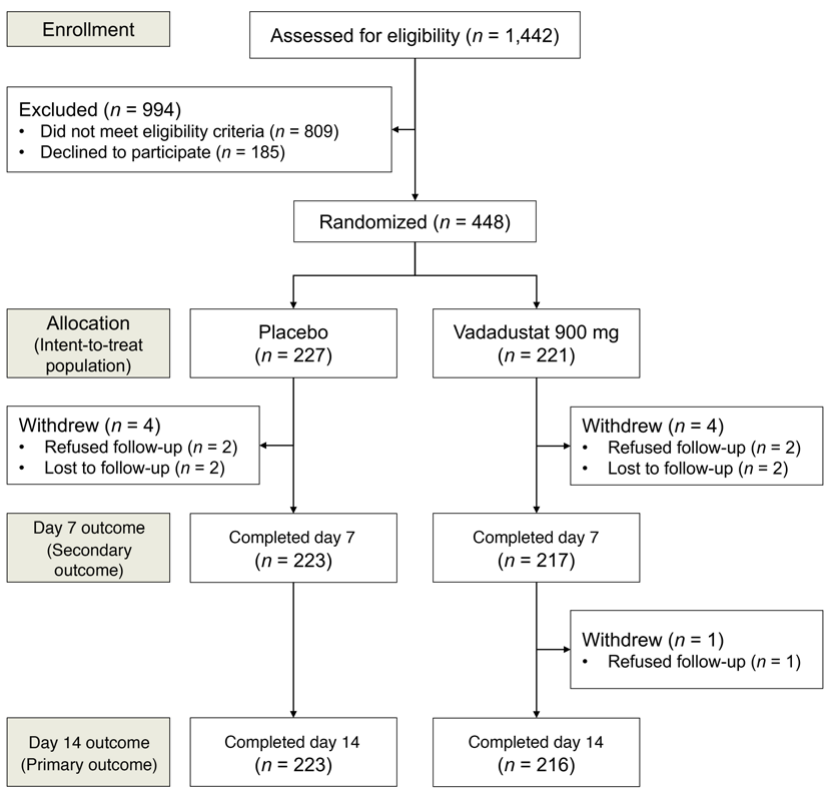
CONSORT diagram of study enrollment.

Our final intent-to-treat analysis included all 448 randomized patients allocated to a study group (Figure 3). Demographic, morphometric, and clinical characteristics were well balanced (Table 1).

#### Safety of vadadustat treatment in hospitalized patients with SARS-CoV-2.

**Figure 4:**
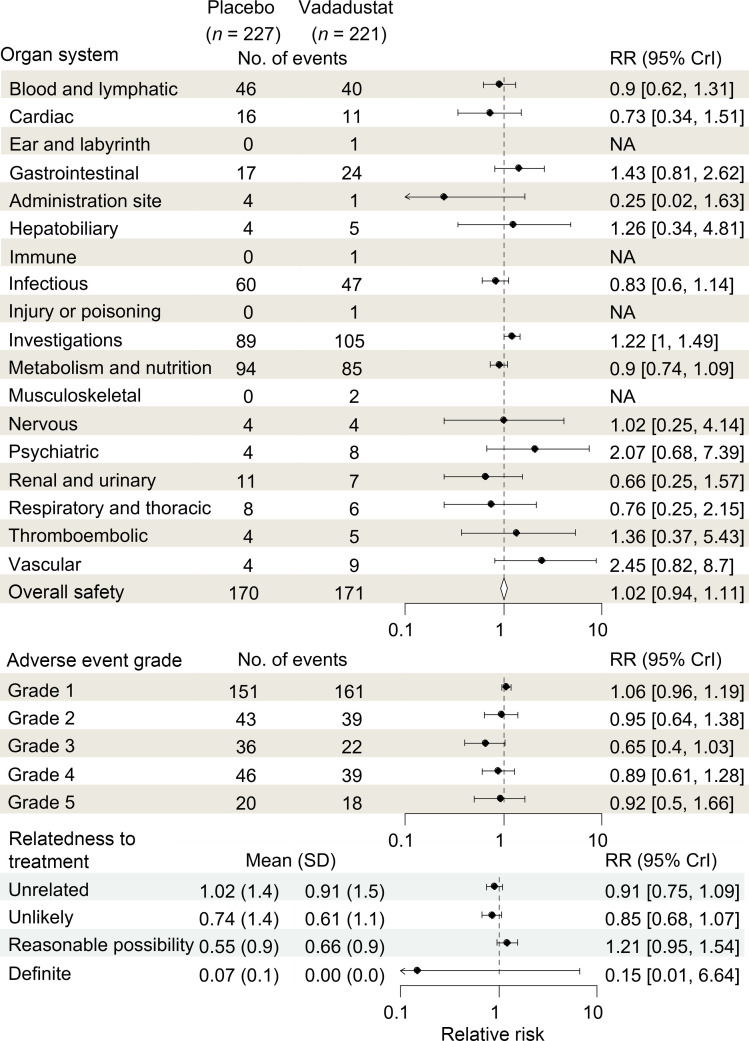
Vadadustat demonstrated a reassuring safety profile in hospitalized patients with SARS-CoV-2–associated lung injury. Forest plot of the favorable safety profile of vadadustat (900 mg daily) compared with placebo. Each category presents the estimated relative risk (RR) and corresponding 95% Bayesian credible interval (CrI), with dots indicating point estimates and error bars representing the CrIs. The overall pooled safety estimate, shown by the diamond, demonstrated vadadustat’s comparable safety relative to placebo across all organ systems, adverse event grades, and relatedness categories. Importantly, no increased risk was observed for thromboembolic events or serious adverse events (grade 3 and higher). These findings provide strong reassurance regarding the safety of vadadustat in hospitalized patients.

Previous studies of vadadustat have primarily evaluated treatment of anemia in outpatients with chronic kidney disease. Our trial differed in evaluating vadadustat in critically ill patients, including those with ARDS, sepsis, and other forms of severe organ injury. This shift in patient population raised salient safety concerns, particularly the risk of serious adverse events, including thromboembolic complications, which are already common in critically ill patients. However, our safety analysis revealed that adverse events were comparable in patients randomized to vadadustat or placebo (relative risk [RR] = 1.02, 95% Bayesian credible interval [CrI] = 0.94 to 1.11; posterior probability RR < 1 = 34.5%) and across all organ systems, adverse event grades, and treatment relatedness (Figure 4). The absence of significant differences in adverse events between vadadustat and placebo groups provides strong reassurance of vadadustat’s safety profile in hospitalized and critically ill patients.

#### Pharmacological HIF activation.

**Figure 5:**
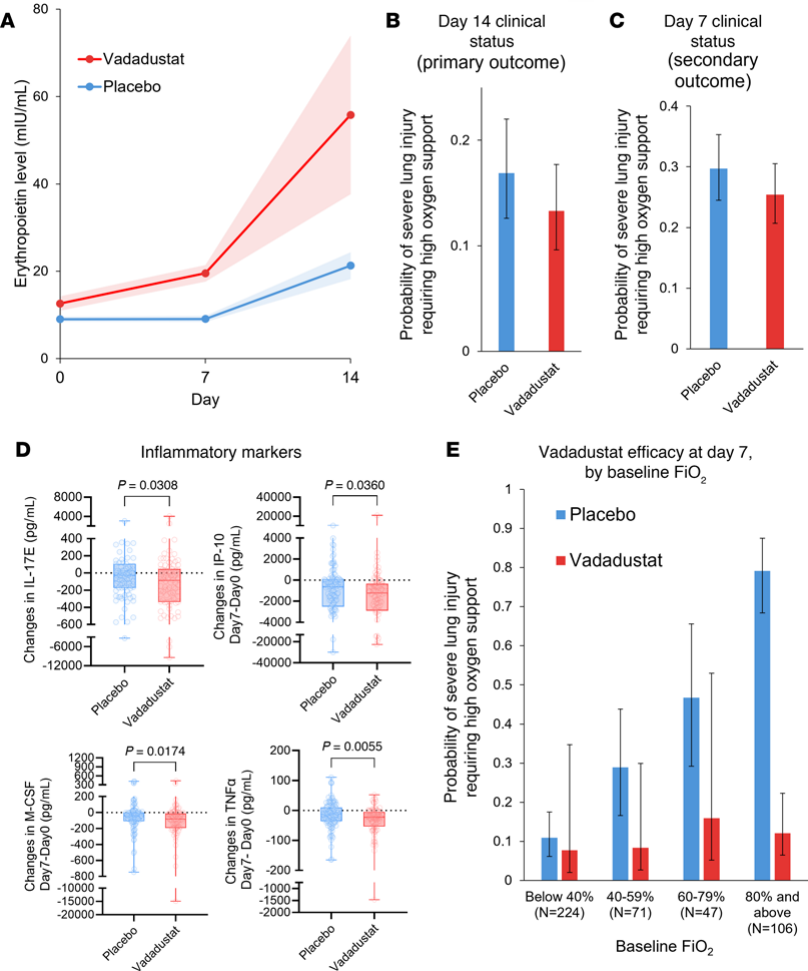
Efficacy of vadadustat in hospitalized patients with SARS-CoV-2 infection and concomitant hypoxia. (**A**) Erythropoietin level in the plasma measured by ELISA showing changes over 14 days. Mean slopes with 95% Bayesian credible intervals (CrIs) are shown, highlighting a 3-fold increase in erythropoietin levels in the vadadustat group (β = 3, 95% CrI = 1.94 to 4.03) compared with placebo (β = 0.85, 95% CrI = 0.61 to 1.08) with a >99% posterior probability (PP) of treatment and time interaction. (**B**) Proportion of patients with severe lung injury requiring high oxygen support (NIAID-OS score ≥ 6) on day 14 (primary outcome). The vadadustat group showed a reduced absolute probability (13.3%) compared with placebo (16.9%), with an absolute risk difference (ARD) of –3.6% (95% CrI = –8.4% to 0.9%). PPs of benefit for ARD < 0% and ARD ≤ –2.5% are 94% and 69%, respectively. (**C**) Proportion of patients with NIAID-OS score ≥ 6 on day 7 (key secondary outcome). Vadadustat treatment demonstrated a higher likelihood of clinical improvement compared with placebo, with an ARD of –4.2% (95% CrI = –9.0% to –0.1%) and a PP of benefit (ARD < 0%) of 97%. (**D**) Inflammatory mediators in the plasma measured by MILLIPLEX Multiplex Assays (IL-17E, IP-10, M-CSF, and TNF-α) between day 0 and day 7. Vadadustat treatment resulted in greater reductions in systemic inflammation compared with placebo, with significant differences noted for each marker. *P* values were obtained by Mann-Whitney *U* test. In the bar-and-whisker plots, the bounds of the boxes represent the 25%–75% interquartile range, the lines within the boxes represent the median, the whiskers represent data min/max, and there are no outlying values. (**E**) Subgroup analysis by FiO_2_ levels upon hospital admission. PPs of clinical benefit for vadadustat (relative to placebo) were highest in patients with baseline FiO_2_ ≥ 80% (PP > 99%), followed by lower probabilities for FiO_2_ 60%–79% (PP = 92%), 40%–59% (PP = 95%), and <40% (PP = 66%).

To assess whether treatment with the HIF stabilizer vadadustat increases HIF activity, we assessed HIF target gene expression. Since HIF stabilizers are used for renal anemia (45, 46), we chose the known target gene erythropoietin to indicate HIF activity (31). Consistent with the effect of vadadustat treatment on HIF stabilization, we observed elevated erythropoietin protein expression in treated patients. Erythropoietin expression increased 3-fold more in patients given vadadustat (β = 3, 95% CrI = 1.94 to 4.03) than placebo (β = 0.85, 95% CrI = 0.61 to 1.08; Figure 5A). Vadadustat dosing in our trial was thus sufficient to substantially augment HIF activity.

#### Efficacy endpoints.

The primary outcome of clinically severe lung injury requiring high-level supplemental oxygen support (National Institute of Allergy and Infectious Diseases Ordinal Scale [NIAID-OS] score ≥ 6) on day 14 occurred in 43 patients in the vadadustat group (estimated probability, 13.3%) compared with 53 patients in the placebo group (estimated probability, 16.9%), representing an absolute risk difference (ARD) of –3.6% (95% CrI = –8.4% to 0.9%) (Figure 5B). The number of patients at each NIAID-OS score is reported in Supplemental Table 1. There was a 69% posterior probability that vadadustat reduced the absolute risk of the primary outcome by 2.5%, which did not meet our strict prespecified criterion for superiority (≥85% posterior probability of ARD ≤ 2.5%). However, there was a 94% posterior probability that vadadustat improved the primary outcome to some degree (ARD < 0%) compared with placebo (Supplemental Table 1 and Supplemental Figure 4A). Additionally, there was a 97.3% posterior probability that vadadustat improved the key secondary outcome of clinically severe lung injury requiring high-level supplemental oxygen support (NIAID-OS score ≥ 6) on day 7 (Supplemental Table 1 and Supplemental Figure 4B). This key secondary outcome occurred more often in the placebo group (estimated probability, 29.7%) than in the vadadustat group (estimated probability, 25.4%), resulting in an ARD of –4.2% (95% CrI = –9.0% to 0.1%; Figure 5C). The additional secondary outcomes are reported in Supplemental Table 2. Vadadustat reduced systemic inflammation on day 7, with substantial decreases in IL-17E, IP-10, M-CSF, and TNF-α (Figure 5D).

#### Subgroup analysis.

We used Bayesian analyses to explore the probability of benefit from vadadustat (relative to placebo) in clinically important subgroups determined by baseline fraction of inspired oxygen (FiO_2_) ranges upon hospital admission, baseline Modified Sequential Organ Failure Assessment (mSOFA) respiratory score, and baseline SpO_2_. The strongest interaction was for FiO_2_ requirement upon hospital admission (posterior probability = 91%), with a benefit being most apparent in patients with high FiO_2_ requirements at hospital admission (Figure 5E). Specifically, there was a greater than 99% chance that treatment with vadadustat was associated with a clinical benefit on reducing lung injury severity in patients with baseline FiO_2_ of 80% or higher.

Lower probabilities of benefit were observed in patients with lower oxygen requirements (FiO_2_ 60%–79%: 92%; FiO_2_ 40%–59%: 95%; FiO_2_ < 40%: 66%). Modest evidence for treatment heterogeneity was observed for baseline SpO_2_ (posterior probability = 66%) (Supplemental Figure 5, A and B) and baseline mSOFA respiratory score (posterior probability = 72%) (Supplemental Figure 5, C and D). These results suggest that vadadustat treatment offers the most benefit in patients who have severe hypoxia and a high oxygen requirement.

## Discussion

HIFs are transcription factors that promote adaptation to hypoxic conditions and other environmental or biological stimuli (12, 31, 47, 48). Recent advancements in molecular targeting have made it a druggable target and both HIF inhibitors and stabilizers have now been FDA approved (31). However, HIF stabilizers are currently approved only for treating anemia in patients with chronic kidney disease (31). HIF stabilizers promote SARS-CoV-2 eradication (23), but it remains unknown whether these drugs improve pulmonary outcomes and survival during SARS-CoV-2 infections in vivo. The contributions of specific functionally important HIF isoforms (HIF1A vs. HIF2A) and cellular sources for HIFs have not been thoroughly characterized. The safety and efficacy of HIF stabilizers in attenuating SARS-CoV-2–associated lung injury in humans similarly remain unknown.

Our murine studies show that treatment with the HIF stabilizer vadadustat provides lung protection and improves survival in murine models and that alveolar epithelium–dependent HIF1A is the critical isoform for lung protection in murine models of SARS-CoV-2–associated lung injury. In translational extensions of these findings to patients with SARS-CoV-2 infection and concomitant hypoxia (SpO_2_ ≤ 94%), we conducted a randomized trial to evaluate the safety and efficacy of HIF stabilizers in human SARS-CoV-2–associated lung injury using the FDA-approved HIF stabilizer vadadustat. Vadadustat did not cause an increase in side effects, including thromboembolic complications, compared to placebo. Treatment tripled serum erythropoietin concentrations by the 14th day, indicating that vadadustat promotes the transcriptional activity of HIFs. Individuals randomized to vadadustat had a 94% likelihood of clinical improvement in the primary outcome compared with placebo (posterior probability ARR ≤ 0% = 94%). The treatment effect was substantially greater in patients who had high oxygen requirements upon hospital admission.

The primary trial outcome did not reach the strict predefined threshold for superiority (≥2.5% decrease in the proportion of patients with NIAID-OS score ≥ 6 with a posterior probability ≥ 85%) on day 14. This strict threshold for superiority was based on a target sample size of 650 patients, but enrollment was stopped early due to declining cases of SARS-CoV-2 infection as the pandemic abated. Nevertheless, on day 7 (key secondary outcome), there was a 97% chance that treatment with vadadustat resulted in meaningful clinical improvement over placebo. Furthermore, subgroup analysis showed strong evidence for progressively increased benefit from vadadustat in patients with severe hypoxemia, as indicated by high baseline oxygen requirements, with the most pronounced effect in patients who required more than 80% inspired oxygen (>99% posterior probability of benefit).

Patients treated with vadadustat had more substantial decreases in inflammatory mediators, including IL-17E, IP-10, M-CSF, and TNF-α, than patients given placebo. Interestingly, a recent in vitro study of alveolar epithelia infected with SARS-CoV-2 shows attenuated viral replication following treatment with a different HIF stabilizer, roxadustat (23). In a hamster model of SARS-CoV-2 infection, roxadustat treatment also reduced the viral load and epithelial damage (24). While promoting microbial defense, the antiinflammatory feature makes HIFs promising therapeutic targets for other respiratory infections. Indeed, HIFs repress respiratory syncytial virus replication, and the HIF stabilizer daprodustat reduces viral load and inflammatory cell infiltration into the lung in murine models of respiratory syncytial virus infection (49). Moreover, roxadustat treatment reduces lung inflammation and improves outcomes in murine influenza A infection (22). Overall, the reassuring safety profile of vadadustat in this patient population combined with the observed biological effects, improvement in clinical outcomes, and the dampening of inflammatory biomarkers are encouraging and warrant additional investigation of HIF stabilizers for treating pathogen-associated lung injury in more pivotal clinical trials.

The benefit of vadadustat treatment can be attributed to various HIF-associated lung protection mechanisms (36, 50–53). Studies in mice indicate that HIF-dependent increases in extracellular adenosine production and signaling through the Adora2A or Adora2b adenosine receptor attenuate inflammation (54) or promote alveolar fluid clearance, thereby dampening pulmonary edema during acute lung injury (55–58). Other studies demonstrate that HIF-dependent induction of vascular endothelial growth factors attenuates vascular leakage, promotes proliferation and repair of alveolar epithelial cells, and helps resolve lung injury (37). Additional experimental evidence indicates that HIFs improve alveolar epithelial carbohydrate metabolism during acute lung injury, thereby reducing lung inflammation in mice (13, 15, 59). HIFs may drive the induction of microRNAs, a group of small noncoding RNAs (60, 61), to repress mediators potentiating lung injury and inflammation. Furthermore, our study implicates HIF1A in alveolar epithelial cells regulating the secretion of chemokines associated with an IFN response. For instance, the fact that IP-10 is increased in mice with alveolar epithelium–specific deletion of HIF1A suggests that HIFa perhaps play a role in pruning IFN responses in epithelial cells. Indeed, previous studies suggested prolonged type I IFN responses increase inflammation and are associated with severe outcomes during SARS-CoV-2 infection (62). These detailed molecular mechanisms will need to be explored further.

Besides alveolar epithelial cells, HIF stabilization could also lead to modulation of adaptive immune responses, such as T cells or B cells, responsible for HIF-mediated lung protection during SARS-CoV-2 infection. For instance, HIF1A promotes Th17 differentiation via direct transcriptional induction of RORγT (63). HIF1A also plays a complex role in Treg cells, inducing Foxp3 as a direct transcriptional target gene, thus promoting Treg differentiation (64), or promoting proteasomal degradation to inhibit Treg differentiation (63). The diverse functional role of HIF1A in Treg cells likely stems from different tissue microenvironments or disease pathogenesis. During respiratory viral infection, B cells are crucial for the production of neutralizing antibodies. HIF stabilization has been implicated in impairing B cell maturation, while hypoxia enhances class switching (65, 66). However, the functional role of HIF1A in B cells during SARS-CoV-2 infection is unclear. Therefore, experimental studies are needed to identify various cellular mechanisms for HIF-dependent lung protection that contribute to dampening acute lung injury associated with SARS-CoV-2 infection.

In summary, the HIF stabilizer vadadustat confers lung protection during SARS-CoV-2–associated lung injury in mice, likely by promoting the stabilization of HIF1A in alveolar epithelial cells. Results from our randomized trial corroborate those findings and suggest that the drug improves pulmonary function in patients admitted with SARS-CoV-2 infection and concomitant hypoxia, especially in those who required considerable oxygen supplementation upon hospital admission. Taken together, our findings provide a strong rationale for pursuing pivotal randomized clinical studies of HIF stabilizers in patients with pathogen-associated acute lung injury.

## Methods

### Sex as a biological variable

Both sexes were involved in human and animal studies included in the study.

### Murine SARS-CoV-2 infection model

#### HIF-transgenic animals, MA10.

In this study mouse lines, including *HIF1a^fl/fl^* (B6.129-*Hif1a^tm3Rsjo^*/J, Jackson Laboratory), *HIF2a^fl/fl^* (stock *Epas1^tm1Mcs^*/J, Jackson Laboratory), *HIF1a^fl/fl^*-UBCCreER, *HIF2a^fl/fl^*-UBCCreER, *HIF1a^fl/fl^*-SPCCreER, *HIF2a^fl/fl^*-SPCCreER, and B6.129S-*Sftpc^tm1^*(*cre/ERT2*)*Blh*/J (Jackson Laboratory), bred in our animal facility, were utilized. Both male and female mice, aged 8–12 weeks, received i.p. injections of tamoxifen (75 mg/kg) at a dose of 1 mg/day for 5 consecutive days, followed by a 7-day recovery period. Subsequently, the mice were infected with the murine-adapted SARS-CoV-2 (MA10) strain via oropharyngeal aspiration (OPA) under anesthesia, with care taken to minimize discomfort. Briefly, animals were lightly anesthetized with 4% isoflurane, positioned at a 45-degree incline, and their tongues gently pulled out with forceps to expose the oropharynx. A viral suspension was administered using a pipette, and the nares were temporarily occluded to ensure proper aspiration into the lungs. Following administration, the mice were placed upright to facilitate airway clearance and returned to their home cages for recovery. After infection, animals were monitored daily for clinical signs and weight changes. At designated time points, mice were euthanized via pentobarbital overdose, and BALF, lung tissue, and blood samples were collected for further analyses.

#### Vadadustat treatment.

Mice were housed in a compliant, controlled environment. The room temperature (RT) was maintained at 20°C–24°C with relative humidity of 40%–60%. A 12-hour light/dark cycle was implemented.

Vadadustat was dissolved in 10% DMSO, 40% PEG 300, 5% Tween 80, and 45% saline to create a concentrated stock solution at 5 mg/mL. For dosage in vivo optimization, a time course study was initiated. Mice were randomly divided into 2 groups: vehicle group and vadadustat treatment group. The vadadustat group received i.p. injection of 50 mg/kg bodyweight at various time points (2 hours, 4 hours, and 74 hours). The vehicle group received i.p. injection of the same volume of vehicle.

In the curative treatment experiment, 2- to 3-month-old K18-hACE2 mice [B6.Cg-Tg(K18-ACE2)2Prlmn/J, Jackson Laboratory] or BALB/cJ (Jackson Laboratory) mice received daily i.p. injection of 50 mg/kg bodyweight of vadadustat 3 days after being infected with 280 PFU of WA1 (BEI, NR-53821) or 200 PFU of MA10 variant ([in isolate USA-WA1/2020 backbone], Infectious Clone [ic2019-nCoV MA10] in Calu-3 cells; BEI, NR-55329). Clinical assessments were conducted to assess the health of individual mice during the experimental period. At the end of certain experiments, lung samples were collected.

### Viral load

We followed the protocol by Mendoza et al. to quantify viral titers (67). Vero E6 cells were seeded into 6-well plates at a density of 3 × 10^5^ cells/mL and incubated overnight at 37°C with 5% CO_2_ to nearly reach confluence. BALF or lung homogenates were serially diluted (10^–1^ to 10^–6^) in high-glucose DMEM supplemented with 2% FBS and added to PBS-washed monolayers. After 1 hour of adsorption at 37°C with gentle rocking, cells were overlaid with a 1:1 mixture of 2× MEM containing 8% FBS and 3% carboxymethyl cellulose to limit viral diffusion. Plates were incubated undisturbed for 72 hours to allow plaque formation. After incubation, overlays were removed, and cells were fixed with 10% formalin and stained with 0.5% crystal violet. Plaques were visualized as clear zones and counted to calculate PFU/mL, adjusting for dilution and sample volume.

### In vivo imaging system detection

For in vivo imaging system (IVIS) imaging studies, ODD-luc HIF reporter mice were injected with intravenous (i.v.) D-luciferin monopotassium salt (Pierce, PI88292; 150 mg/kg) and euthanized 5 minutes after the injection. Lungs were imaged ex vivo using bioluminescence imaging mode on the IVIS imager (Lumina III) with serial exposure time. Region of interest (ROI) was defined by circling the lung tissue and bioluminescence intensity was calculated using IVIS software (Living Image).

### Immunoblotting

To examine the levels of HIF1A and HIF2A protein expression, frozen lung tissues were homogenized and the protein lysates were extracted by tissue protein extraction reagents (Thermo Fisher Scientific). The tissue homogenates were centrifuged at 12,000*g* at 4°C for 15 minutes and then supernatant, containing the proteins, was transferred to new tubes. Protein concentrations were determined by using Bradford assay reagent (Bio-Rad). The immunoblotting samples for SDS-PAGE were prepared by mixing the protein lysate with 4× Laemmli sample buffer (Bio-Rad) supplemented with 10% β-mercaptoethanol (Sigma-Aldrich), and then the mixtures were heated at 95°C for 10 minutes. Approximately 20 μg of protein per well was separated based on molecular weight using a 7.5% SDS-PAGE precast gel (Bio-Rad), transferred to a 0.45 μm poly-vinylidene fluoride (PVDF) membrane (Bio-Rad), and blocked in 5% skim milk in PBST. Membranes were probed with respective primary antibodies targeting Hif2a (NB 100-122, Novus; diluted 1:2000 in PBST), Hif1a (14179, Cell Signaling Technology; diluted 1:2000 in PBST), or α-tubulin (2144, Cell Signaling Technology; diluted 1:2000 in PBST) and incubated overnight at 4°C with gentle swirling. After incubating, the membranes were washed with PBST and incubated with secondary anti-rabbit (7074, Cell Signaling Technology; diluted 1:2000 in PBST) for 1 hour. A ChemiDoc Touch Imaging System (Bio-Rad) was used for the detection of protein bands. ImageJ software (NIH) was used for densitometry.

### Quantification of HIF target genes

RNA used for Figure 1E was extracted from the lung tissues with QIAzol (Qiagen) and the RNA was precipitated with isopropanol. One microgram of RNA was used for cDNA synthesis. The real-time qPCR was performed with SYBR reagent (GenDEPOT) with specific primers (MilliporeSigma) targeting each transcript. *Rps3* was used for the housekeeping gene. Primer sequences are RPS3-F: ATGGCGGTGCAGATTTCCAA; RPS3-R: GTAACTCGGACTTCAACTCCAG; mVegf-F: CTTGTTCAGAGCGGAGAAGC; mVegf-R: ACATCTGCAAGTACGTTCGTT; mEpo-F: AATGGAGGTGGAAGAACAGGCCAT; mEpo-R: CGAAGCAGTGAAGTGACGCTACGTA; mGlut1-F: CAGTTCGGCTATAACACTGGTG; mGlut1-R: GCCCCCGACAGAGAAGATG; mCd73-F: CTATGAGCCTCTTGAAATGG; mCd73-R: CTGATATCTTGATCACCAGAG; mAdora2b-F: CGCTCAGGTATAAAGGTTTG; mAdora2b-R: CACTGTCTTTACTGTTCCAC.

### Erythropoietin ELISA

Blood was collected from patients into red cap blood collection tubes and allowed to clot. Serum samples were collected by centrifugation at 1500*g* for 10 minutes at 4°C. The serum was aliquoted and stored at –80°C until analysis. Prior to performing the assay, serum from each sample was removed from storage, thawed on ice, and gently spun down at 3000*g* for 5 minutes. The supernatant was carefully collected for the assay.

Serum erythropoietin levels were quantified using the Human Erythropoietin Quantikine IVD ELISA Kit (DEP00, R&D Systems), following the manufacturer’s protocol with minor modifications. All reagents and the kit were brought to RT for at least 30 minutes before use. A total of 100 μL of assay diluent was added to each well, followed by 100 μL of undiluted serum samples or standards. Most serum samples were analyzed undiluted, but samples that exhibited saturated signals were diluted in 1:5, 1:10, or 1:50 ratios as necessary to fall within the assay’s dynamic range. The plate was sealed and shaken at RT for 1 hour at 500 rpm. Following incubation, the solution was aspirated without washing. Next, 200 μL of conjugate was added to each well, and the plate was resealed and shaken again at RT (500 rpm) for 1 hour. Afterward, the wells were aspirated and washed 4 times with 400 μL of 1× wash buffer. To initiate the colorimetric reaction, 200 μL of substrate solution was added to each well, and the plate was incubated at RT for 20 minutes. The reaction was stopped by adding 100 μL of stop solution to each well. Absorbance was measured at 450 nm within 15 minutes, with a wavelength correction at 600 nm. Erythropoietin concentrations were calculated by comparison with a standard curve, and assay sensitivity, as well as intra- and interassay variability, met the manufacturer’s specifications.

### Cytokine/chemokine multiplex immunoassays

The concentrations of various cytokines, chemokines, and growth factors in human plasma samples were measured using the MILLIPLEX Human Cytokine/Chemokine/Growth Factor Magnetic Bead Panel A (HCYTA-60K-PXBK48, MilliporeSigma). This multiplex assay facilitates the simultaneous quantification of 48 analytes using Luminex xMAP technology. However, in our study, the analysis of RANTES was excluded, resulting in measurements for 47 cytokines and chemokines.

Plasma samples were collected from study patients into EDTA tubes and immediately processed by centrifugation at 1500*g* for 10 minutes at 4°C. The plasma was aliquoted and stored at –80°C until analysis. Prior to performing the assay, plasma from each sample was removed from storage, thawed on ice, and gently spun down at 3000*g* for 5 minutes. The supernatant was carefully collected for the assay.

The assay was performed following the manufacturer’s instructions. Briefly, plasma samples were diluted 1:2 in assay buffer, and 25 μL of each sample was added to the wells of a 96-well plate precoated with magnetic beads specific to the 47 analytes. A standard curve was generated using the provided recombinant cytokine and chemokine standards, ranging from 3.2 to 10,000 pg/mL. The plate was incubated at RT for 2 hours with continuous shaking to allow for the binding of cytokines and chemokines to their respective capture beads. Following incubation, the plate was washed 3 times using a handheld magnetic plate washer to remove unbound material. Afterward, 25 μL of biotinylated detection antibodies were added to each well and incubated for 1 hour at RT with gentle shaking. The plates were washed again to remove excess detection antibodies, and 25 μL of streptavidin-phycoerythrin was added to each well, followed by incubation for 30 minutes at RT in the dark. The beads were resuspended in 150 μL of sheath fluid after the final wash, and the plate was read using a Luminex 200 instrument (Luminex Corporation). Data acquisition was performed using xPONENT software, with cytokine and chemokine concentrations calculated based on a 5-parameter logistic (5-PL) regression standard curve.

The results were analyzed using GraphPad Prism and analyte concentrations were expressed in pg/mL. All samples were assayed in duplicate, and intra- and interassay coefficients of variation were calculated to ensure precision.

### Human research study (phase II clinical trial)

This Bayesian multicenter, double-blind, placebo-controlled, randomized clinical trial assessed the safety and efficacy of vadadustat in hospitalized patients with SARS-CoV-2 infections and concomitant hypoxia (SpO_2_ ≤ 94%). The complete protocol and statistical analysis plan are provided in the Supplemental Methods.

The trial was conducted at 5 hospital sites across 2 large Houston health care systems. Eligible patients were adults aged 18 years or older with a confirmed SARS-CoV-2 infection by real-time PCR and concomitant hypoxia, defined as an SpO_2_ of 94% or less on room air. All met inclusion and exclusion criteria detailed in the full protocol.

Patients were randomized to vadadustat or identical-looking placebo, with both patients and investigators blinded to treatment assignments. Randomization was performed using a web-based system and stratified by site and organ injury severity (mSOFA score < 4 and ≥ 4) with block sizes of 2 or 4.

Patients randomized to vadadustat were given 900 mg orally daily, while reference patients were given a matching placebo. Treatment continued for 14 days while patients remained hospitalized. All patients received standard care for COVID-19 per institutional protocols, which could include supplemental oxygen, antiviral therapies, corticosteroids, and other supportive measures.

#### Outcomes.

The primary outcome was the proportion of patients on day 14 after randomization who had an NIAID-OS score of 6 or higher, a clinical measure of lung injury severity (68). The definitions of the NIAID-OS scores relevant to the primary outcome are as follows: 5, hospitalized and requiring low-flow supplemental oxygen; 6, hospitalized and requiring noninvasive ventilation or use of high-flow oxygen devices; 7, hospitalized and receiving invasive mechanical ventilation or extracorporeal membrane oxygenation (ECMO); and 8, death. A key secondary outcome was the proportion of patients with an NIAID-OS score of 6 or higher on day 7. Additional exploratory outcomes included the proportion of patients with a mSOFA score of 0 (indicating normal organ function) at 14 days, time to hospital discharge, and the following measures on day 7 and day 14: average mSOFA scores, ventilator-free survival, overall survival, incidence of hypotension (mean arterial pressure < 70 mmHg or requirement for inotropes or vasopressors to maintain blood pressure), and incidence of acute kidney injury (Supplemental Table 2). Safety endpoints included the incidence of adverse events and serious adverse events outlined in the full protocol (Supplemental Methods). Safety events were categorized by organ systems, adverse event grade, and relatedness to treatment. All safety endpoints were also evaluated on an ongoing basis during the trial by an independent data and safety monitoring board (DSMB) after 30, 120, and 400 study patients were randomized and when the trial was concluded.

### Statistics

For our clinical trial’s primary outcome, we used Bayesian analyses involving a large, credible interval (0.002–486.6) as the prior distribution to express initial uncertainty regarding the treatment effect (Supplemental Methods). Bayesian generalized linear modelling was used to evaluate treatment superiority. Primary, secondary, and safety outcomes were compared on day 7 and day 14. The differential linear change in erythropoietin levels was modeled as a function of the interaction between time and treatment group. Heterogeneity of treatment effects was tested via Bayesian analyses of the interaction of the treatment group with baseline FiO_2_, SpO_2_ (<92% vs. ≥92%), and mSOFA respiratory score (0–2 vs. 3–4). Follow-up analyses evaluated the probability of benefit of vadadustat relative to placebo in subgroups of patients at different baseline FiO_2_ levels. Expression of inflammatory biomarkers was compared with Mann-Whitney *U* tests.

All analyses were conducted on the intent-to-treat population, without imputing missing outcomes. Sensitivity analyses evaluated the robustness of the conclusions to missing data (Supplemental Methods). The trial was designed to accrue the largest possible sample size given available resources (*N* = 650; *n* = 325/group). Based on this expected sample size, a strict decision threshold for treatment superiority was predefined as a posterior probability of ≥85% of an absolute decrease of ≥2.5% in the percentage of patients with an NIAID-OS score of ≥6 on day 14 (primary outcome). The trial was approved to enroll up to 650 hospitalized COVID-19 patients; however, the trial was stopped early by blinded trial investigators in consultation with the DSMB in March 2022 due to declining cases of SARS-CoV-2 infection.

### Study approval

Animal care was performed according to the NIH *Guide for the Care and Use of Laboratory Animals* (National Academies Press, 2011). All experimental procedures were approved by the UTHealth Institutional Animal Care and Use Committee (protocols AWC-20-0120 and AWC-24-0083). All mice utilized in the study were from breeding colonies within UTHealth. Our clinical trial was approved by the UTHealth Institutional Review Board (protocol HSC-MS-20-0395) and written informed consent was received from all patients before enrollment. The trial was registered at ClinicalTrials.gov (NCT04478071) before the first patient was enrolled.

### Data availability

Deidentified patient-level data are available from the corresponding authors on a collaborative basis. Values for all data points in graphs are reported in the Supporting Data Values file. The study protocol and statistical analysis are included in the manuscript.

## Author contributions

All authors had access to and vouch for the trial data. BB, PP, SDL, XY, and HKE wrote the first draft of the manuscript. DR and PBS assisted with studies related to human COVID-19. Five authors (KF, JK, YW, IKB, and TM) led the murine SARS-CoV-2 infection model. PP, MFD, GWW, DIS, AT, AAG, and SKB were part of the data interpretation and manuscript revision process. Three authors (CG, CDD, and RS) verified the underlying data and led the analyses. YL and HL assisted with revisions and manuscript writing during the revision process. All authors gave final approval for manuscript submission. BB, SL, and PP share first authorship of this manuscript. BB and SL designed the study, conducted the experiments, acquired data, and wrote the manuscript. PP analyzed data, interpreted data, and wrote the manuscript. The order of co–first authors was assigned alphabetically among equally contributing authors.

## Supplementary Material

Supplemental data

Unedited blot and gel images

Supporting data values

## Figures and Tables

**Table 1:**
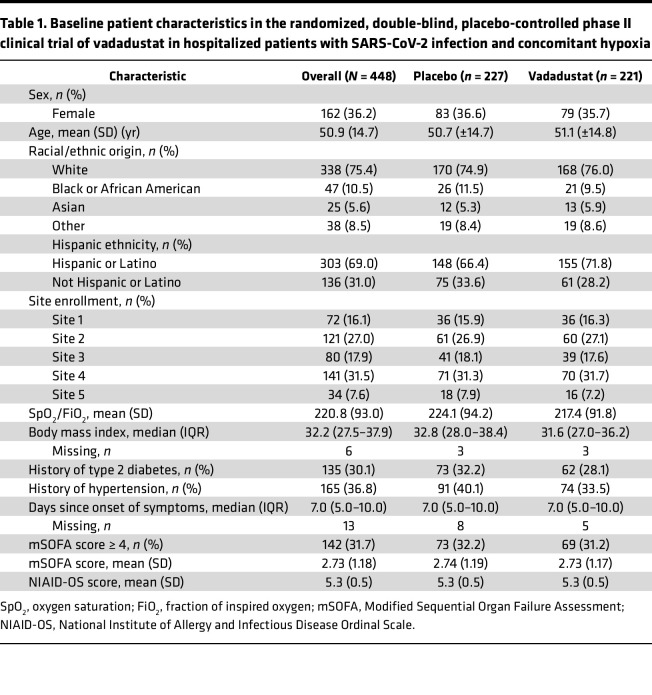
Baseline patient characteristics in the randomized, double-blind, placebo-controlled phase II clinical trial of vadadustat in hospitalized patients with SARS-CoV-2 infection and concomitant hypoxia

## Version 1 06/17/2025

In-Press Preview

## Version 2 07/22/2025

Electronic publication
