# Supplementary material for: Identification of HIF1A as a therapeutic target during SARS-CoV-2–associated lung injury
Source: JCI Insight. 2025 Jun 17;10(14):e191463. doi: 10.1172/jci.insight.191463 (PMC12288972; doi:10.1172/jci.insight.191463)
Supplement: Supplemental data [file jciinsight-10-191463-s221.pdf]

Supplementary Materials

Table S1. Bayesian Analysis of Primary and Key Secondary Outcomes

Outcome	Placebo (*n* = 227)	Vadadustat (*n* = 221)	Absolute Risk Difference [ARD] (95% CrI)	Posterior Probability (ARD < 0)
**NIAID-OS at Day 14 (Primary Outcome), *n* (%)**	53 (16.9)	43 (13.3)	-3.6% (-8.4% to 0.9%)	94%
NIAID-OS 6: Hospitalized, on non-invasive ventilation or high flow oxygen devices	28 (12.3)	19 (8.6)		
NIAID-OS 7: Hospitalized, on invasive mechanical ventilation or ECMO	19 (8.4)	17 (7.7)		
NIAID-OS 8: Death	6 (2.6)	7 (3.2)		
**NIAID-OS at Day 7 (Key Secondary Outcome), *n* (%)**	88 (29.7)	72 (25.4)	-4.2% (-9.0% to 0.1%)	97.3%
NIAID-OS 6: Hospitalized, on non-invasive ventilation or high flow oxygen devices	79 (34.8)	63 (28.5)		
NIAID-OS 7: Hospitalized, on invasive mechanical ventilation or ECMO	6 (2.6)	9 (4.1)		
NIAID-OS 8: Death	3 (1.3)	0 (0)		

Abbreviations: *NIAID-OS*, National Institute of Allergy and Infectious Disease Ordinal Scale; *ARD*, absolute risk difference; *ECMO*, extracorporeal membrane oxygenation

Table S2. Secondary Outcomes

Outcome	Overall (*N* = 448)	Placebo (*n* = 227)	Vadadustat (*n* = 221)
**mSOFA Score of 0 at Day 14 (Secondary Outcome), *n* (%)**	313 (69.9)	157 (69.2)	156 (70.6)
**Additional Secondary Outcomes at Day 7**			
Average mSOFA Score, mean (SD)	2.13 (2.75)	2.31 (3.01)	1.93 (2.44)
Ventilator Free Survival, *n* (%)	280 (62.5)	135 (59.5)	145 (65.6)
Overall Mortality, n (%)	3 (0.7)	3 (1.3)	0 (0.0)
Hypotension at Day 7, *n* (%)	20 (4.5)	11 (4.8)	9 (4.1)
Acute Kidney Injury, *n* (%)	12 (2.7)	7 (3.1)	5 (2.3)
**Additional Secondary Outcomes at Day 14**			
Average mSOFA Score, mean (SD)	1.88 (4.23)	1.88 (4.09)	1.87 (4.37)
Ventilator Free Survival, *n* (%)	343 (76.6)	170 (74.9)	173 (78.3)
Overall Mortality, *n* (%)	13 (2.9)	6 (2.6)	7 (3.2)
Hypotension, *n* (%)	24 (5.4)	10 (4.4)	14 (6.3)
Acute Kidney Injury, *n* (%)	12 (2.7)	7 (3.1)	5 (2.3)

Abbreviations: *mSOFA*, modified sequential organ failure assessment; *SD*, standard deviation

SUPPLEMENTAL FIGURE 1:

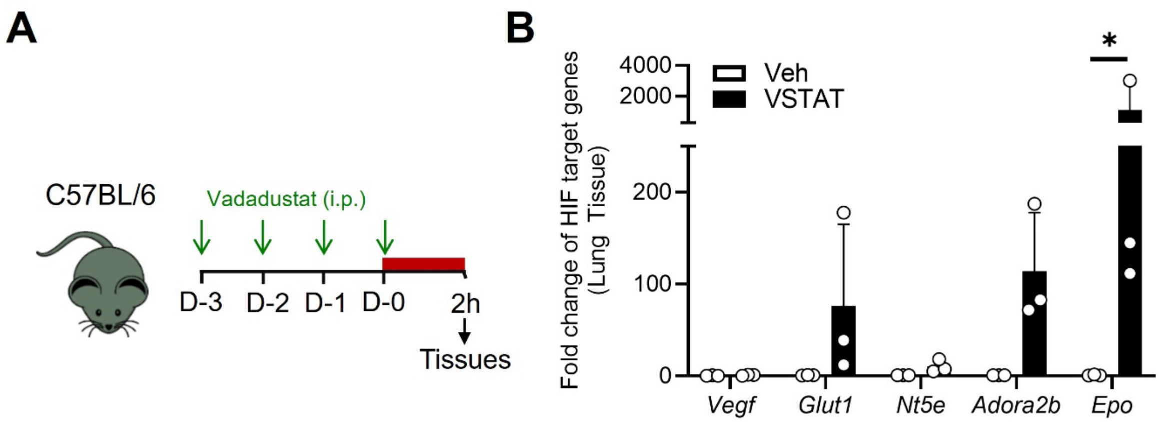

Fig. S1. The impact of HIF stabilizer vadadustat treatment on HIF target genes in the lung. **(A)** Schematic diagram of vadadustat treatment on C57BL/6 mice. **(B)** HIF target genes were measured in RNA isolated from whole lung tissue after treatment with the vadadustat by RT-qPCR. Each column represents one specific target gene. Data is presented as the means and standard deviations. *$p<0.05$, Two-Way ANOVA test.

SUPPLEMENTAL FIGURE 2:

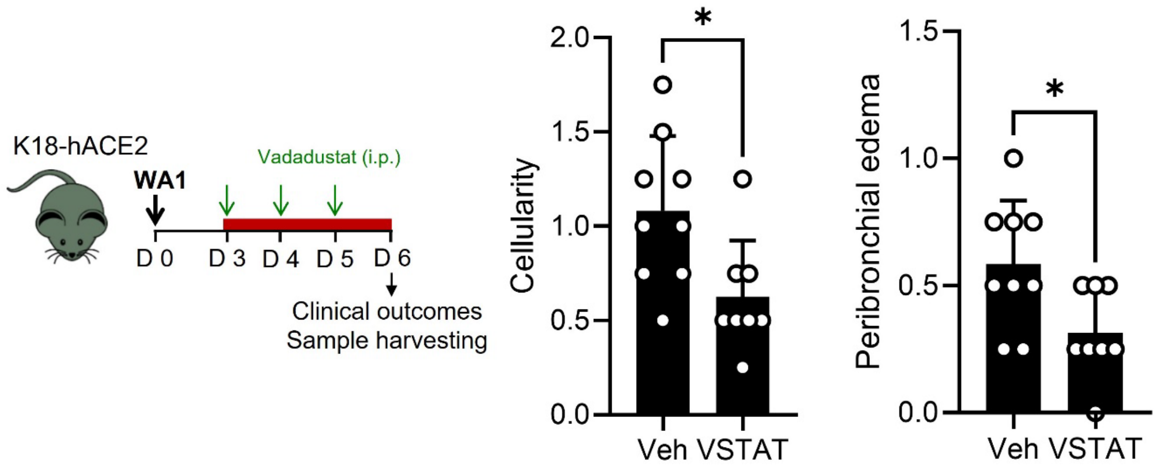

Fig. S2. HIF stabilizer vadadustat treatment reduces acute lung injury scoring in the K18-hACE2 models of SARS-CoV-2 infection. Schematic diagram of WA1 infection (280PFU) in K18-hACE2 mice treated with vadadustat. Blinded histological injury scores of the lungs were quantified as described in the materials and methods. Data are represented as mean ± standard error of the mean. *P < 0.05, **P < 0.01, Student t-test.

SUPPLEMENTAL FIGURE 3:

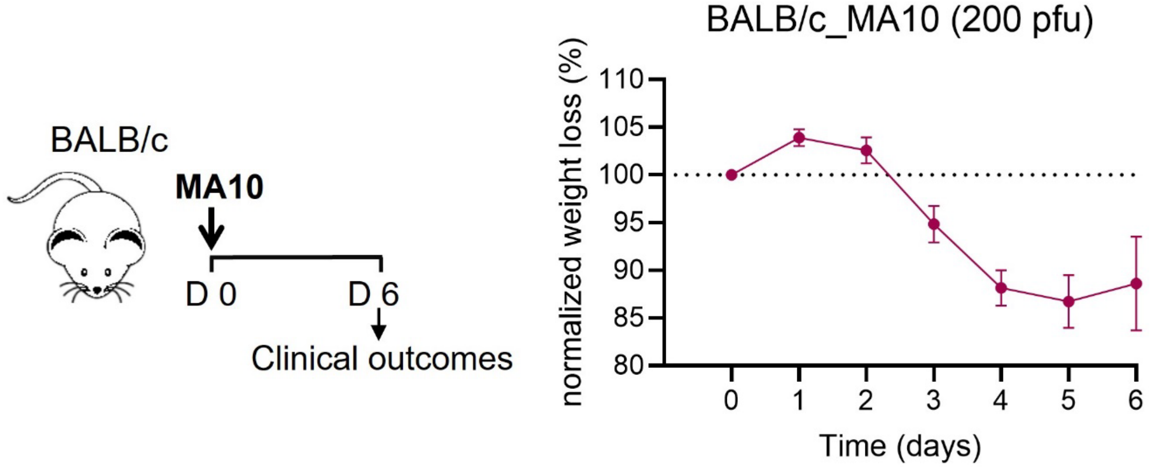

Fig. S3. Disease progression of SARS-CoV-2 infection (MA10) in BALB/C mice. Schematic diagram of MA10 infection (200 PFU) in BALB/c mice. Weight was tracked for 6 days post-infection. Data is presented as the means and standard deviations.

SUPPLEMENTAL FIGURE 4:

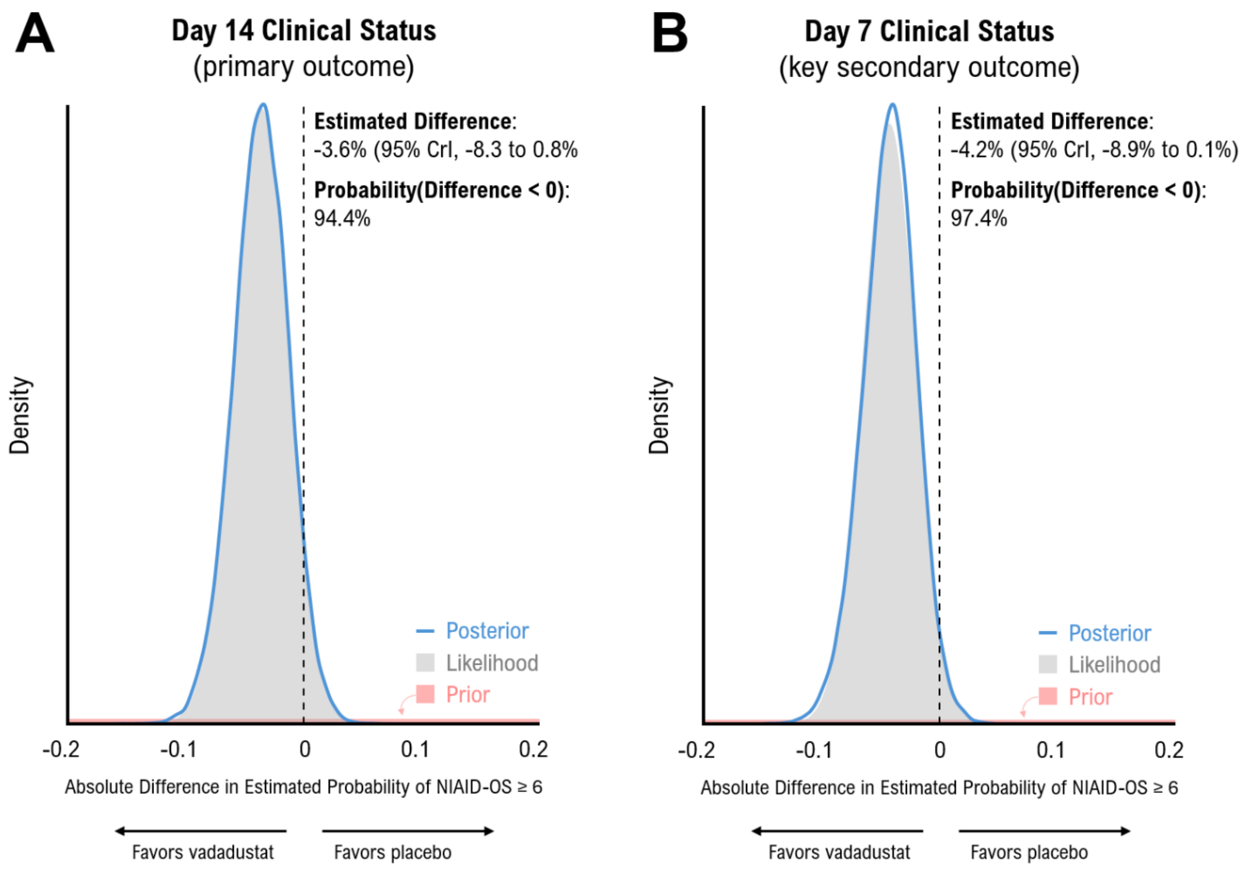

Fig. S4. Bayesian efficacy analysis of the primary outcome and key secondary outcome for vadadustat in hospitalized patients with SARS-CoV-2 infection and concomitant hypoxia. The Bayesian efficacy analysis used a diffuse neutral prior (normal distribution, $\mu = 0$, $\sigma^2 = 10$). This prior distribution (*pink shaded area*) reflects minimal prior assumptions about the treatment effect of vadadustat, allowing observed trial data (likelihood, *gray shaded area*) to primarily influence the final posterior distribution estimates (*blue line*). This prior distribution was selected to provide stable estimation while reflecting initial uncertainty about the efficacy of vadadustat (i.e., we assumed that vadadustat was neither beneficial nor harmful). The graphs show each component of the Bayesian analysis— prior distribution, likelihood, and posterior distribution—of the difference in the outcome incidence between the vadadustat group and the

placebo group. (**A**) For the primary outcome of severe lung injury requiring high oxygen support (NIAID-OS score ≥ 6) at day 14, the vadadustat group had an 3.6% lower absolute incidence of the outcome compared to placebo. This represents a 94.4% posterior probability of benefit with vadadustat compared to placebo. (**B**) For the key secondary outcome of severe lung injury requiring high oxygen support at day 7, the vadadustat group had a 4.2% lower absolute incidence of the outcome compared to placebo. The posterior probability of benefit with vadadustat for this key secondary outcome was 97.4%.

SUPPLEMENTAL FIGURE 5:

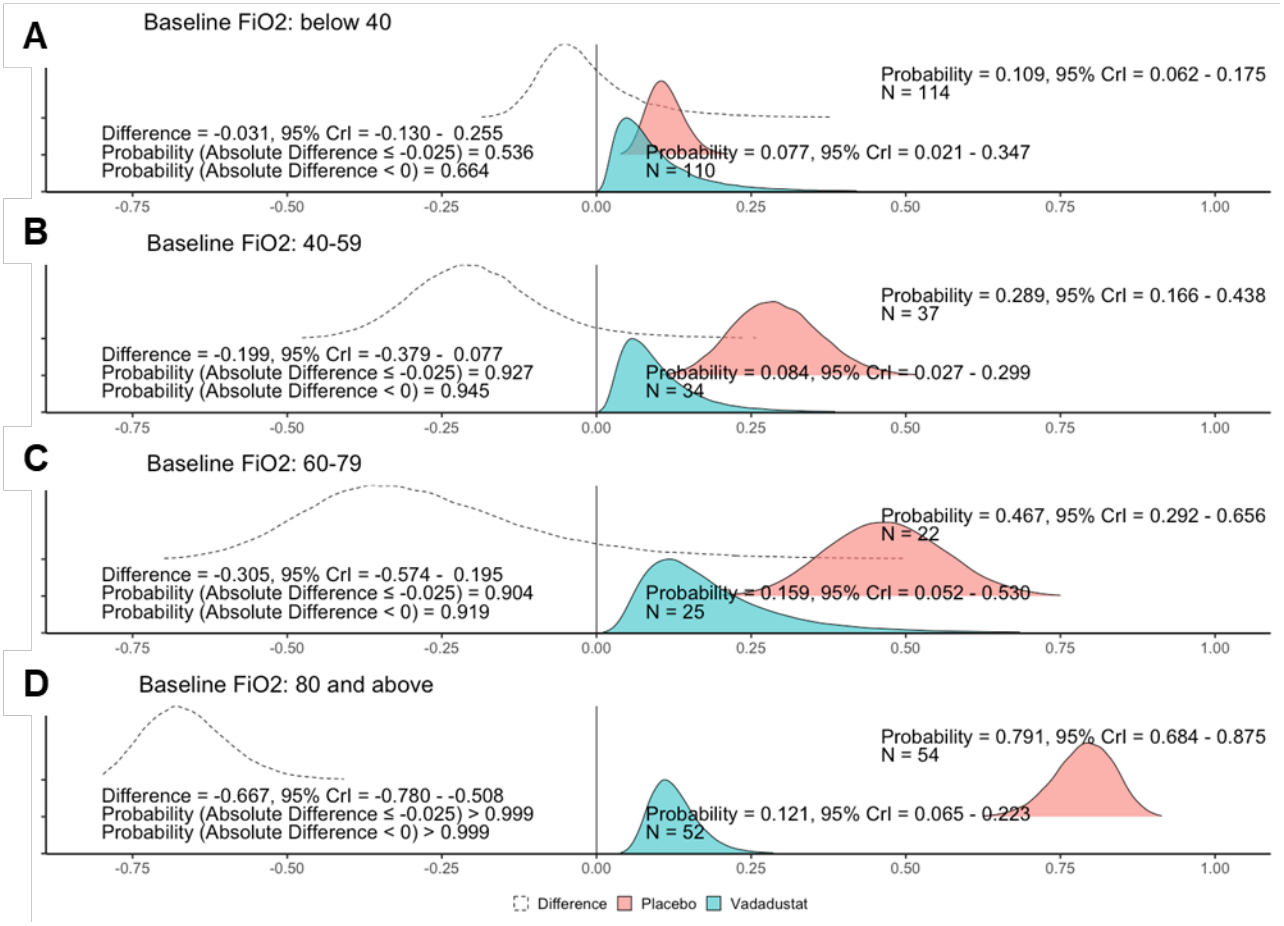

Fig. S5. Bayesian subgroup analysis of the efficacy of vadadustat at FiO_2 on admission. The posterior distributions of the probability of severe lung injury requiring high oxygen support (NIAID-OS ≥ 6) at day 7 for vadadustat (*blue shaded area*) and placebo (*orange shaded area*) alongside the difference in the outcome between the vadadustat and placebo groups (*dashed line*). (**A**) In patients receiving FiO_2 < 40% on admission (*n* = 224), patients treated with vadadustat had a 3.1% lower incidence of the outcome, representing a 66.4% probability of benefit from vadadustat compared to placebo. (**B**) A higher probability of benefit from vadadustat of 94.5% was observed in patients receiving 40-59% FiO_2 on admission (*n* = 71), with an estimated 19.9% lower incidence of the outcome in patients receiving vadadustat

compared to placebo. (**C**) Among patients with an FiO_2 60-79% on admission (*n* = 47), the vadadustat group had a 30.5% lower incidence of the outcome compared to the placebo group, representing a 91.9% probability of benefit. (**D**) In the subgroup of patients receiving the highest level of oxygen support with FiO_2 > 80% on admission (*n* = 106), vadadustat provided the strongest benefit with a >99.9% posterior probability of benefit over placebo. In this subgroup, patients treated with vadadustat had a 66.7% (95% CrI, 50.8-78%) lower outcome incidence compared to patients who received placebo.

Appendix

VSTAT COVID: Study Protocol

**Protocol Title:**	VSTAT COVID: Vadadustat Study Targeting ARDS Therapy in COVID and VSTAT-LoTuS: A Long-Term Follow-Up Study of COVID-19 patients treated with either vadadustat or placebo.
**Principal Investigator:**	Bentley Bobrow, MD (Akebia), Holger Eltzschig, MD, PhD (DoD)
**Co-Investigators:**	Elizabeth Jones, MD, Samuel Luber, MD, Paulina Sergot, MD, George Williams, MD, David Robinson, MD, Richard Gordon, MD, Benjamin Karfunkle, MD, Katheryn Larimore, MD, Rachel Bower, MD, Emily Ayub, MD
**Study Coordinators:**	Misty Ottman, BSN, RN, Misha Granado, MPH, MS, Christian Noval, MCR, BSN, RN, Katelyn Stout, BSN, RN, Neomi Sepulveda, BS, Talha Mubashir, BSc, MD, Marium Kaukab, MBBS, MD, Ashley Holt, RN, Alfredo Castellano, MBBS
**Sponsor:**	The University of Texas Health Science Center, 6431 Fannin Street, Houston, Texas 77030
**Study Type:**	Randomized, double-blind, placebo-controlled clinical trial
**Study Number:**	HSC-MS-20-0395
**ClinicalTrials.gov Identifier:**	NCT04478071
**Investigational Drug:**	Vadadustat
**Investigational New Drug (IND):**	150183
**Phase of Development:**	Phase 2
**Population:**	Up to 650 adult patients with COVID-19 hospitalized for hypoxemia
**Number of Sites:**	Multicenter
**Study Duration:**	18 months concluding December 31, 2023
**Patient Duration:**	28 days (24 months for the long-term follow-up)
**Protocol Version:**	Original Protocol Version 2.0 (17th June 2020) Revised Dec 23, 2021

Signature Page Protocol Approval

01/20/2022

Signature **Date**

Bentley Bobrow, MD
Professor and Chair, Department of Emergency Medicine
McGovern Medical School
The University of Texas Health Science Center at Houston

1. General Information

Vadadustat is a synthetic, orally bioavailable, small molecule which acts as an inhibitor of hypoxia-inducible factor prolyl-hydroxylases (HIF-PHs) and is currently being developed as a potential therapy for anemia of chronic kidney disease. This clinical trial aims at evaluating the efficacy of vadadustat on the prevention and treatment of ARDS in hospitalized patients with COVID-19

2. Background Information

2.1. Oxygen Homeostasis and Hypoxia-Inducible Factor

Erythropoiesis is closely regulated in response to changes in tissue oxygenation levels. It is controlled in a large part by changing levels or the oxygen-sensitive transcription factor known as Hypoxia Inducible Factor, or HIF. HIF largely exists as two related but distinct isoforms: HIF-1 and HIF-2. HIF controls pathways that facilitate oxygen delivery to cells and allow adaptation to hypoxia (Siddiq et al., 2007), including the expression of EPO (Peyssonnaux et al., 2007; Semenza, 1999).

The transcriptionally active form of HIF is a heterodimeric protein consisting of an oxygensensitive HIF-α subunit and a constitutively expressed HIF-β subunit. Under normoxic conditions, the HIF-α subunit is produced and continuously degraded with a $t_{1/2}$ of approximately 5 minutes (Berra et al., 2001). This degradation is initiated by a family of enzymes known as the HIF-prolyl hydroxylase domain, (or HIF-PHD or HIF-PH) enzymes (Bishop and Ratcliffe, 2014; Ke and Costa, 2006). The HIF-PHs function as critical sensors of cellular oxygen levels, and regulate oxygen homeostasis in response to hypoxia through inhibition of hydroxylation thus leading to stabilization of HIF-α, which in turn signals gene transcription and translation of proteins necessary for the cellular and organ responses to poor oxygenation (Myllyharju, 2013; Siddiq et al., 2007).

2.2. Inhibition of HIF PHD enzymes

The general, molecular mechanism of action proposed for small molecule HIF-PHD inhibitors such as vadadustat has been described extensively (Joharapurkar et al., 2018; Rabinowitz, 2013) and is illustrated in the Figure 1. Under conditions of tissue oxygen repletion (normal O_2, top Figure 1), the HIF-PHD enzymes rapidly hydroxylate HIF signaling its degradation in the proteasome. However, under low oxygen conditions (bottom, Figure 1), the HIF-PHD enzymes are substrate (O_2) deficient

and inhibited leading to the accumulation of the HIF- subunit, translocation to the nucleus and activation of transcriptional protocols that signal hypoxia-protective pathways. Vadadustat acts in a manner similar to physiological hypoxia, by inhibiting PHD enzymes in the active site, leading to stabilization and increased levels of HIF-α, and upregulation of hundreds of genes involved in cellular protection from and adaptation to pathologically low oxygen levels (Haase, 2017; Rabinowitz, 2013).

Figure 1 Mechanism of Action of Vadadustat

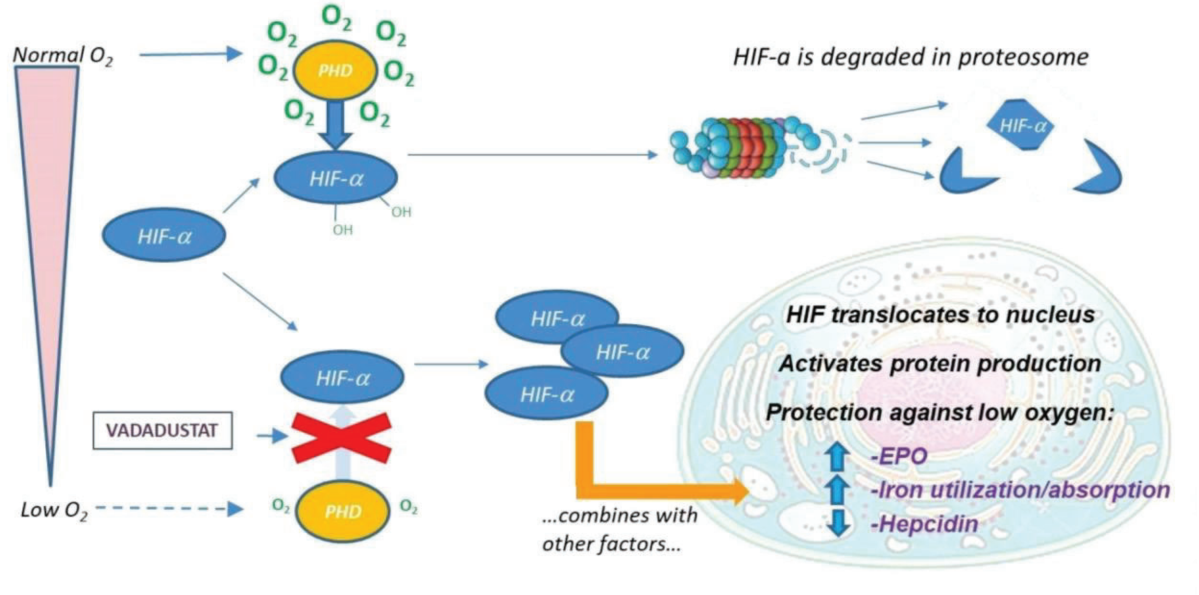

2.3. Vadadustat

Vadadustat is a small molecule active-site inhibitor of the HIF-PHD enzymes, the inhibition of which leads to increases in cellular HIF—the "hypoxic response" (see Figure 1). HIF-PHD enzymes are 2-oxoglutarate-dependent dioxygenases that require molecular oxygen in order to affect hydroxylation of proline residues in the oxygen-dependent degradation domain of HIF-α, and require Fe^{2+} and ascorbate as essential cofactors (Appelhoff et al., 2004; Masson and Ratcliffe, 2003). Each of the 3 known human HIF-PH isoenzymes regulate HIF-1α and HIF-2α in a nonredundant manner that is dependent upon the degree of hypoxia and the relative abundance of the specific HIF-PH isozyme in different tissues (Appelhoff et al., 2004; Fong and Takeda, 2008; Kant et al., 2013). Thus, pharmacologic inhibition of HIF-PHs represents a novel therapeutic approach to the treatment of anemia and also cellular and organ protection against ischemic insult (Lee et al., 2019; Schodel and Ratcliffe, 2019).

2.4. The Role of HIF in Acute Lung Inflammatory Diseases such as COVID-19 and Associated Organ Failure

Coronavirus disease 2019 (COVID-19) is a recently emerged respiratory disease caused by severe acute respiratory distress syndrome coronavirus 2 (SARS-CoV-2). During the past months, COVID- 19 has become a pandemic with currently over 2 million patients affected worldwide and over 600,000 documented infected patients only in the US (https://coronavirus.jhu.edu/map.html). While most patients with COVID-19 only experience mild to moderate symptoms, approximately 15% will proceed to severe pneumonia and about 5% among those patients will go on to experience life- threatening

acute respiratory distress syndrome (ARDS) (Cao, 2020; Huang et al., 2020; Wu et al., 2020; Xu et al., 2020; Zhou et al., 2020).

ARDS is an acute form of lung disease characterized by acute bilateral pulmonary edema, severe hypoxia – usually requiring intubation and mechanical ventilation (Ranieri et al., 2012; Thompson et al., 2017). Using the 2012 Berlin Definition (Table 2), stages of mild, moderate, and severe ARDS are associated with increased mortality (27%; 95% CI, 24%-30%; 32%; 95% CI, 29%34%; and 45%; 95% CI, 42%-48%) and increased median duration of mechanical ventilation in survivors (5 days; interquartile [IQR], 2-11; 7 days; IQR, 4-14; and 9 days; IQR, 5-17, respectively).

Table 1: 2012 Berlin Criteria for ARDS

Lung injury of acute onset, within 1 week of an apparent clinical insult and with progression of respiratory symptom
Bilateral opacities on chest imaging (chest radiograph or CT) not explained by other lung pathology (e.g., effusion, lobar/lung collapse, or nodules)
Respiratory failure not explained by heart failure or volume overload
Decreased PaO2/FiO2 ration measured with a minimum of 5 mmHg of positive end expiratory
Severity:<br>▯ Mild ARDS: 201 – 300 mmHg<br>▯ Moderate ARDS: 101 – 200 mmHg<br>▯ Severe ARDS: $\leq$100 mmHg

Therapeutic approaches for ARDS are very limited and currently focus on vigilant supportive care, prevention of nosocomial infections, and protective mechanical ventilation (Eltzschig et al., 2014; Thompson et al., 2017; Vohwinkel et al., 2015; Ware and Matthay, 2000). Similarly, treatment options for COVID-19 or COVID-19 associated ARDS are extremely limited. During COVID associated ARDS, an uncontrolled immune response with excessive infiltration of myeloid inflammatory cells and uncontrolled lung inflammation are characteristic features (Cao, 2020). The search for anti-inflammatory treatment approaches that could dampen excessive immune activation as a therapeutic strategy to decrease COVID-associated ARDS mortality is an area of intense investigation (Wu et al., 2020). Based on an extensive literature documenting effects as well as organ-protective effects of HIF stabilization in the area of acute injury, we propose vadadustat may offer benefit to patients suffering from COVID-19 and related acute respiratory syndromes.

2.4.1. Lung Protection

During conditions of hypoxia and ischemia, HIF is stabilized and triggers a wide-ranging of adaptive responses through activation of hypoxia-responsive gene products, such as erythropoietin (Eltzschig and Carmeliet, 2011; Semenza, 2011; Semenza, 2012). Several HIF target-genes have been implicated in improving ARDS outcomes: HIF-dependent increases in extracellular adenosine signaling (Eltzschig et al., 2012; Idzko et al., 2014a; Idzko et al., 2014b), increases in the glycolytic capacity of alveolar epithelia (Eckle et al., 2013a), or other anti-inflammatory signaling pathways that promote the resolution of mucosal inflammation (Clambey et al., 2012; Eckle et al., 2013a; Eckle et al., 2008a; Eckle et al., 2007; Eckle et al., 2014; Hoegl et al., 2015; Kiers et al., 2018; McClendon et al., 2017;

Mirakaj et al., 2010; Morote-Garcia et al., 2009; Mutz et al., 2010; Reutershan et al., 2009; Rosenberger et al., 2009). For example, experimental studies indicate that during ARDS, HIF is stabilized and provides protection from ARDS, as part of an endogenous protective pathway that could be targeted therapeutically (Eckle et al., 2013a; McClendon et al., 2017). Studies in mice with targeted deletion of HIF in alveolar epithelia revealed a more severe degree of ARDS, including increased pulmonary edema, increased lung inflammation and attenuated survival time. Similarly, studies utilizing pharmacologic inhibitors of the HIF-pathway demonstrated increased lung injury and attenuated ARDS survival time. In contrast, pretreatment with pharmacologic HIF-activators is associated with protection from ARDS induced by mechanical ventilation including attenuated pulmonary edema, improved gas exchange and improved survival (Eckle et al., 2013a). Taken together, these studies provide strong experimental evidence showing that HIF activators can be used therapeutically to prevent or treat lung inflammation during ARDS.

Acute lung injury (ALI) leads to pulmonary edema and hypoxia. Increase permeability of endothelial cells leads to protein rich fluid accumulation in alveoli (smallest airspaces in the lung) and thereby impairing gas (oxygen or O_2 and carbon dioxide or CO_2) exchange (Shimoda and Semenza, 2011; Urrutia and Aragones, 2018; Vohwinkel et al., 2015). Hypoxia itself may contribute to lung injury and thereby perpetuate the injury by impairing endothelial cells, attracting white blood cells, and promoting inflammation. Stabilizing HIF has been shown to be protective during ALI (Huang et al., 2019; Li et al., 2020a; Nagamine et al., 2016). HIF-1 stabilization has been shown to reduce I preclinically through both optimizing carbohydrate metabolism (shift to glycolysis) (Eckle et al., 2013a) and through upregulation of anti- inflammatory adenosine signaling (Bowser et al., 2017; Eckle et al., 2008a; Eckle et al., 2008b; Eckle et al., 2013b; Eckle et al., 2014; Hoegl et al., 2015) as well as improvements in general somatic epithelial and endothelial barrier integrity (Clerici and Planes, 2009; Gong et al., 2015; Huang et al., 2019; Kapitsinou et al., 2014; Rajendran et al., 2020).

Long-term upregulation of HIF may have deleterious effects on *chronic* organ inflammatory conditions such as pulmonary hypertension, pulmonary fibrosis, and chronic obstructive pulmonary disease, CKD, etc. although this seems less relevant to the acute application envisioned here (Fu and Zhang, 2018; Li et al., 2020b; Luo et al., 2015).

2.4.2. Protection of Other Organs Against Failure During Ischemia

In addition to its potential beneficial effects with respect to the treatment of anemia, and acute lung inflammation, HIF may have protective effects during ischemia, reperfusion, hypoxia, and inflammatory conditions (Heyman et al., 2016). Pharmacologic inhibition of PHDs is associated with increased HIF stabilization – even under normoxic conditions – and concomitant organ protection, including the heart, intestine, kidneys, liver as well as the lungs (Bowser et al., 2017; Eckle et al., 2013a; Eckle et al., 2012; Eckle et al., 2008d; Hill et al., 2008; Kapitsinou et al., 2014; Robinson et al., 2008).

The myriad potential benefits if HIF stabilization related to hypoxia have been extensively reviewed (Colgan et al., 2020; Fraisl et al., 2009; Lee et al., 2019; Prabhakar and Semenza, 2015; Zhu et al., 2019). In general:

- Hypoxia results in inflammation and stabilization of HIF can dampen tissue inflammation and promote repair (Colgan et al., 2020; Cummins and Taylor, 2017).
- HIF stabilization upregulates glycolytic enzymes that adapt tissue to oxygen deprivation and anaerobic energy production (Koivunen and Kietzmann, 2018).
- HIF promotes EPO and VEGF production. EPO increases red blood cell production and hemoglobin that carry oxygen to tissues. VEGF increases blood vessel growth to hypoxic tissue. The combination leads to increased oxygen delivery to hypoxic tissues (Pugh and Ratcliffe, 2003; Tanaka et al., 2015).
- HIF increases extracellular adenosine by stimulating genes that convert extracellular ATP to ADP, AMP, and eventually adenosine. HIF also upregulates adenosine receptors leading to cell signaling that decreases cellular inflammation (Eckle et al., 2014; Hart et al., 2011).
- Stabilizing HIF either pharmacologically or genetically, has been shown to afford protection against ischemic and other damage in multiple organs apart from lung: o Heart (Eckle et al., 2008c; Natarajan et al., 2006; Sridharan et al., 2007) o Kidney (Eckardt et al., 2007; Ito et al., 2020; Yang et al., 2018) o Liver (Guo et al., 2015; Ju et al., 2016; Schneider et al., 2010)
- HIF is a potent stimulator of EPO production, and EPO has been shown to have significant protective effects in the lung after ALI through a number of different protective mechanisms (Heitrich et al., 2016; Rocha et al., 2015; Zhu et al., 2019).

2.5. Vadadustat in Clinical Trials

A total of 34 Akebia studies spanning Phase 1 through 3 have been completed or are ongoing. In the studies completed to date (Phase 1 and 2 studies), a total of 1011 subjects have received vadadustat, including 553 healthy volunteers, 450 subjects with CKD, and 8 subjects with hepatic impairment. In 4 ongoing Phase 3 studies, 7237 subjects have received study medication (vadadustat or darbepoetin alfa).

Generally, in completed vadadustat studies with healthy volunteers, there were a low number of adverse events (AEs). The most frequently reported AEs in healthy volunteers were in gastrointestinal system disorders (nausea, diarrhea, abdominal pain, flatulence, dyspepsia) and nervous system disorders (headache, dizziness). The majority of the events were mild to moderate in severity. There were no serious adverse events (SAEs), deaths, or withdrawals due to AEs. There were no clinically relevant changes in mean laboratory values (including vascular endothelial growth factor or adrenal-related biomarkers), vital signs, or electrocardiograms (ECGs).

In completed Phase 2 studies, the most frequently reported AEs in subjects with CKD were in gastrointestinal disorders (nausea, diarrhea, vomiting), cardiovascular disorders (hypertension, hypotension, coronary artery disease), renal disorders (renal failure chronic, renal failure acute), infections and infestations (gastroenteritis, urinary tract infection, pneumonia), and metabolism and nutrition disorders (hyperkalemia, fluid overload).

The safety profile to date supports the ongoing clinical development of vadadustat as an orally bioavailable agent for the treatment of anemia associated with CKD.

2.6. COVID-19 and ARDS

Patients with COVID-19 present primarily with fever, myalgia/fatigue, or dry cough. In most cases, COVID-19 results in mild symptoms and patients can recover in 1 to 2 weeks. Most patients have a favorable prognosis; however, older patients and those with chronic underlying conditions may have worse outcomes. Patients with severe illness may develop dyspnea and hypoxemia within 1 week of onset, with the potential to progress to ARDS or end-organ failure (Wu et al, 2020). Typical findings of chest CT images of COVID-19 positive patients who are in the ICU include bilateral multiple lobular and subsegmental areas of consolidation. Analysis of 1324 laboratory-confirmed cases in Wuhan China showed that fever (87.9%) and cough (67.7%) were among the most common symptoms. Lymphopenia was observed in 82.1% of patients admitted to the ICU (Jin et al, 2020).

In a study of 5700 patients hospitalized with COVID-19 in New York City, the median age of patients affected was 63, with 39.7% female patients and 60.3% male patients. Common comorbidities included cancer (6%); cardiovascular disease, including hypertension (56.6%), coronary artery disease (11.1%), and congestive heart failure (6.9%); chronic respiratory disease, including asthma (9%), chronic obstructive pulmonary disease (5.4%), and obstructive sleep apnea (2.9%); immunosuppression, including HIV (0.8%) and history of solid organ transplant (1%); kidney disease, including chronic (5%) and end-stage disease (3.5%), and metabolic disease, including obesity (41.7%), morbid obesity (19%), and diabetes (33.8%). Among the 2634 patients who were discharged or died at the study endpoint, 373 (14.2%) patients were treated in the ICU, and 12.2% received invasive mechanical ventilation (Richardson et al, 2020). As of April 4, 2020, out of the patients requiring mechanical ventilation, 38 (3.3%) were discharged alive, 282 (24.5%) died, and 831 (2.2%) remained hospitalized. A total of 45 patients (2.2%) were readmitted during the study period.

In a study of 41 patients hospitalized with COVID-19 in Wuhan China, patients with severe illness developed ARDS and required ICU submission and oxygen therapy. The median time from onset of symptoms to first hospital admission was 7 days, to shortness of breath was 8 days, to mechanical ventilation was 10.5 days, and to ICU admission was 10.5 days. The median time to ARDS from onset of symptoms was 9 days, the time between hospital admission and ARDS being as short as 2 days (Huang et al, 2020).

2.6.1. COVID-19 and Hemoglobin

The mean hemoglobin levels for patients infected with COVID-19 across five studies is shown in Table 2.

Table 2: Mean Hemoglobin Levels in COVID-19 Positive Patients

**Study**	**Country**	**Sample Size (n)**	**Mean Hb g/dL**
Clinical Characteristics of Coronavirus Disease 2019	China	1099	13.4
Epidemiologic Features and Clinical Course of Patients Infected with SARS-CoV-2 in Singapore	Singapore	18	13.5

Clinical course and outcomes of critically ill patients with SARS-CoV-2 pneumonia in Wuhan, China: A single-centered, retrospective, observational study	China	34	12.8
Clinical Features of patients infected with 2019 novel coronavirus in Wuhan, China	China	41	12.6
Epidemiological and clinical characteristics of 99 cases of 2019 novel coronavirus pneumonia in Wuhan, China: a descriptive study	China	99	13.0

Source: Chen et al, 2020; Guan et al, 2020; Huang et al, 2020; Young et al, 2020; Yang et al, 2020

In 122 COVID-19 patients admitted to UT Health, the mean Hb on admission was 11.8, S.D. = 2.36. During the course of their hospitalization the mean low Hb for these patients was 10.53, S.D. = 2.45, with 12/3% (n = 15) receiving transfusions.

2.7. Bayesian Statistical Methods

Developing effective interventions for acute respiratory distress syndrome (ARDS) requires incremental improvement of theoretically sound treatments based on systematically accruing data. Often this incremental development is hampered by statistical tools not appropriate to the task. Classical, Frequentist statistics have advanced the field, but are less informative for the initial test of a new treatment. The reliance of the Frequentist framework on dichotomous, null hypothesis testing provides some control of the error rate in the context of multiple repeated trials; however, this is not what early-phase treatment testing requires. Developing nascent treatments requires investigators to bet on an alternative hypothesis. Investigators evaluating a theoretically sound intervention want to know the probability that the approach confers some level of benefit given the observed data: that is, they want to know the probability that the alternative hypothesis is true. While Frequentist inference does not directly address this issue, Bayesian statistical inference provides a principled approach to answer this question. Indeed, addressing the so-called "Pipeline Problem" in developing clinical applications, the FDA has indicated that Bayesian statistics offers one avenue for improved methodological efficiency (FDA. Innovation/Stagnation 2004, FDA. Innovation/Stagnation 2006, Irony 2008, O'Neill RT 2006, Woodcock 2005, Fitzmaurice & Laird 2000).

Decision-making based on an initial treatment trial is assisted by estimates of the probability of an effect of some specified magnitude. These statements, not part of the conventional, Frequentist statistical lexicon, are accessible via Bayesian approaches, particularly with small sample sizes (Lilford et al 1995, Spiegelhalter et al 1999). Detailed descriptions of Bayesian statistical reasoning exist elsewhere (Berry 2006, Goodman 2005, Wijeysundera et al 2009). Succinctly, Frequentist models estimate the probability of observing the data (or data more extreme) given that the null hypothesis is true; Bayesian analyses estimate the probability of the alternative hypothesis given the observed data (Wijeysundera et al 2009). Bayesian probability estimates incorporate prior information about plausible parameter values (i.e., the prior distribution) and the observed data (i.e., the likelihood). Combining these two distributions forms the posterior distribution which permits evaluation of the probability that the true value of the parameter falls in some range.

3. Primary Objectives

To assess the efficacy and safety of vadadustat for the prevention and treatment of ARDS in hospitalized patients with COVID-19

4. Study Design

This is a phase 2 multicenter, randomized, double-blind, placebo-controlled Bayesian clinical trial of vadadustat for the prevention and treatment of ARDS in hospitalized patients with COVID-19. Extended follow-up of participants will occur at 6, 12, and 24 months (see section 8.4.1).

Following screening, patients will be randomized in a 1:1 ratio to either vadadustat 900 mg or placebo. Randomization will be stratified by site, and MSOFA score at baseline (<4 and ≥4)

Figure 1: Study Schematic

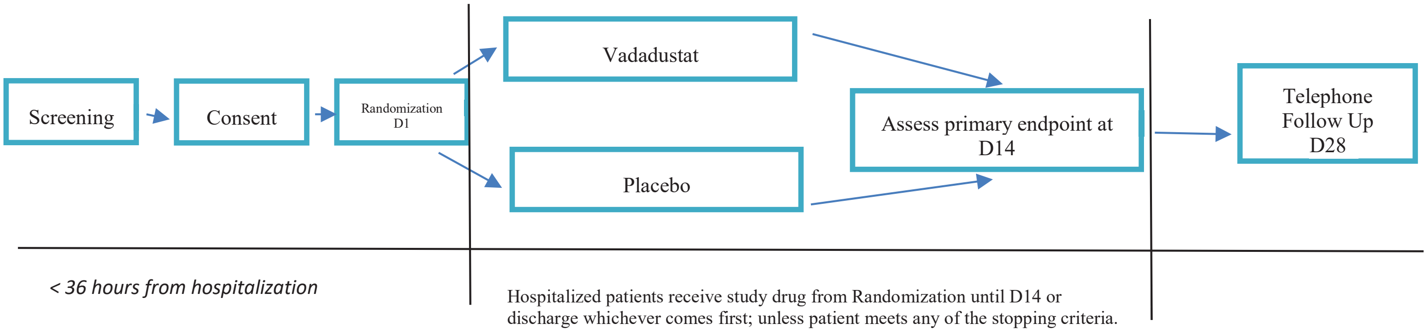

5. Study Outcomes:

5.1. Assessment of Efficacy

5.1.1. Primary Outcome

The proportion of patients at day 14 who are dead (8) or hospitalized, on invasive mechanical ventilation or ECMO (7) or hospitalized, on non-invasive ventilation or high flow oxygen devices (6) on the NIAID-OS.

Table 4: National Institute of Allergy and Infectious Disease Ordinal Scale (NIAID-OS)

Death	8
Hospitalized, on invasive mechanical ventilation or extracorporeal membrane oxygenation (ECMO)	7
Hospitalized, on non-invasive ventilation or high flow oxygen devices	6
Hospitalized, requiring supplemental oxygen	5
Hospitalized, not requiring supplemental oxygen – requiring ongoing care (COVID19 related or otherwise)	4

Hospitalized, not requiring supplemental oxygen – no longer requires ongoing medical care	3
Not hospitalized, limitation on activities and/or requiring home oxygen	2
Not hospitalized, no limitations on activities	1

Source: https://clinicaltrials.gov/ct2/show/NCT04421027?cond=COVID19&lead=Lilly&draw=2&rank=1

5.1.2. Secondary Outcomes:

Proportion of patients with a score of 0 at 14 days on the MSOFA scale.

Table 3: Modified Sequential Organ Failure Assessment (MSOFA) Score

Organ System	0	1	2	3	4
Respiratory SpO2/FiO2	> 400	≤ 400	≤ 315	≤ 235	≤ 150
Liver	No scleral icterus or jaundice			Scleral icterus or jaundice	
Cardiovascular, hypotension	No hypotension	MAP < 70 mm Hg	Dopamine ≤ 5 or dobutamine any dose	Dopamine > 5 Epinephrine ≤ 0.1 Norepinephrine ≤ 0.1	Dopamine > 15 Epinephrine > 0.1 Norepinephrine > 0.1
CNS, Glasgow Coma Score	15	13 – 14	10 – 12	6 – 9	< 6
Renal, Creatinine mg/dL	< 1.2	1.2 – 1.9	2.0 – 3.4	3.5 – 4.9	> 5.0

Source: Grissom et al, 2010

Note: Discharged patients will be assigned a 0 score and dead patients a score of 20.

5.1.3 Other Outcomes:

- Average mSOFA scores at day 7 and day 14, gathered either from in-hospital labs and clinical notes for inpatients, or from calling discharged patients
- Ventilator-free survival at day 7 and day 14, as assessed by the patient's best score on the NIAID-OS on day 7, day 14 and day 28
- Overall survival at day 7, day 14 and day 28
- Proportion of patients with hypotension (MAP <70 mm Hg or requirement for inotropes or vasopressors to maintain blood pressure) at day 7 and day 14
- Proportion of patients with acute kidney injury at day 7 and day 14
- Time to hospital discharge

5.2. Assessment of Safety

- Incidence of adverse events (AEs)
- Incidence of serious AEs (SAEs)
- Incidence of thromboembolic events
- Creatinine, bilirubin, AST and ALT, hemoglobin

In addition to the ongoing and final safety assessments through the course of the trial, a detailed review of safety by the DSMB will follow the ascertainment of the 14-day outcomes on the first 30 and 120 patients. After 30 and 120 patients have received treatment and have completed their day 14 event, enrollment will be suspended (suspension of enrollment will occur at n = 30, based on DSMB recommendation, enrollment will continue during the safety analysis at n = 120). An interim blinded safety report will be sent to the DSMB. Enrollment will continue according to DSMB recommendations. The DSMB will provide a report to the UTHealth COVID-19 Research Workgroup and UTHealth IRB to consider further patient screening and enrollment.

5.3. Exploratory Outcomes

Efficacy of vadadustat to induce known transcriptional targets of HIFs (e.g., erythropoietin, extracellular adenosine levels, circulating levels of CD73, VEGF, hypoxia-driven microRNAs that dampen lung inflammation) will be explored.

Various laboratory measurements (e.g., serum creatinine, FGF-23, markers of attenuated inflammation) will be measured and summarized.

6. Study Population

6.1. Eligibility Criteria

6.1.1. Inclusion Criteria

1. Men and women ≥ 18 years of age
2. Laboratory-confirmed diagnosis of COVID-19 by detection of SARS-CoV-2 RNA by RTPCR from any specimen respiratory
3. Admitted to the hospital within 36 hours
4. For patients admitted WITH respiratory symptoms, enrollment must occur within 36 hours of hospital admission. If the patient is admitted to the hospital with normal saturations, but develops respiratory symptoms DURING their hospital stay, enrollment may occur within 24 hours of desaturation to 94% or less on room air.
5. Oxygen saturation of hemoglobin by pulse oximetry at room air ≤94%
6. Understands the procedures and requirements of the study and provides written informed consent and authorization for protected health information disclosure

6.1.2. Exclusion Criteria

1. Hypersensitivity to vadadustat or any of its excipients
2. Placed on mechanical ventilation before randomization
3. Hemoglobin above the gender-specific upper limit of normal (ULN) at randomization: 16 g/dL for females and 18 g/dL for males
4. Patients who have erythrocytosis or polycythemia vera
5. Patient taking Probenecid, lopinavir or ritonavir.
6. Women who are pregnant or breastfeeding, or positive pregnancy test before randomization
7. Patients not on maintenance dialysis with eGFR< 31ml/min.
8. Patients who have received a solid organ transplant, heart, kidney, liver or lung
9. Patients who are prisoners
10. Patients who are currently Do Not Resuscitate (DNR) or Do Not Intubate (DNI)
11. Patients with modified Rankin scale of 3 or greater
12. Patient who are currently enrolled in any other interventional clinical trial
13. Acute thromboembolism
14. Home oxygen (O2)

7. Study Drug

7.1. Dosage

Vadadustat 900 mg or matching placebo

7.2. Route and Form

Oral 150 mg tablets and matching placebo

7.3. Administration to Intubated Patients

Tablets can be crushed using a pill crusher and administered to an intubated patient through a nasogastric tube. Tablets may be dissolved in warm water after cutting the pill in half.

The research pharmacist or designee will dispense study drug to the patient and maintaining study drug accountability as described below.

7.4. Vadadustat

Refer to the Vadadustat Pharmacy Manual for details on the shipping, dispensing, storage, destruction, and accountability of vadadustat tablets.

7.4.1. Storage and Supplies

Vadadustat will be provided as 150 mg, white to off-white, round, biconvex film-coated, debossed or non-debossed tablets for oral administration. The tablets and matching placebo will be packaged in high density polyethylene bottles with child resistant closures, polypropylene liner, and induction seal.

Vadadustat and placebo will be stored per the investigational study drug label in a temperature controlled, locked facility, accessible only to authorized study personnel.

7.4.2. Dispensing of Vadadustat or Placebo

Patients will receive one of the following doses for 14 days, or until their date of discharge, or whichever comes first, vadadustat 900 mg (6 tablets once daily) or matching placebo (6 tablets once daily) without regard for meals at approximately the same time each day.

7.4.3. Vadadustat Accountability and Destruction

All vadadustat will be accounted for and all discrepancies explained. The research pharmacy is responsible for keeping accurate records of the clinical supplies received from Akebia Therapeutics (Akebia) all supplies retained in inventory at the study site, and study drug dispensed to or returned from each patient. Records will be maintained that accurately reflect the drug accountability of vadadustat.

Drug accountability includes, but is not limited to:

- Monitoring expiration dates.
- Verifying physical inventory matches documented inventory.

Verifying accountability records are completed for all vadadustat received, dispensed, and returned, and that all required fields are complete, accurate, and legible. During the study, the research pharmacist will be notified of any expiration dates or retest date extensions of study drug. If an expiration date notification is received during the study, the study site will complete all instructions outlined in the notification, including segregation of expired study drug for return to the Akebia or its designee for destruction as specified by the Akebia.

Prior to study site closure and at appropriate intervals during the study, the research pharmacist of designee will perform study drug accountability and reconciliation.

At the end of the study, the investigator will retain all original documentation regarding study drug accountability, return, and/or destruction, and copies will be sent to the sponsor.

All unused and/or partially used vadadustat will be returned to Akebia or destroyed at the study site, as specified by Akebia. Appropriate records of the disposal will be documented and maintained. No unused vadadustat may be disposed of until fully accounted for by Akebia or designee. Empty containers may be disposed of according to local procedures with the permission of Akebia.

7.4.4. Dosing rationale

The planned dosing is a fixed daily dose of 900 mg (supplied as 6-150 mg tablets). We believe that the dose and magnitude of HIF stabilization needed to induce the hypothesized beneficial pulmonary effects are the same as that needed to induce erythropoiesis. In healthy volunteers who received single doses of vadadustat, the median tmax ranged between 3.5 hours for the subjects who were administered 900 mg. The peak erythropoietin concentration was reached after 18 hours from dosing. In healthy volunteers who received 10 daily doses of vadadustat, the mean change in

hemoglobin from baseline to day 11 among those who took vadadustat 900 mg was 0.43 g/dL. From published literature on COVID-19 patients, the median duration from onset of illness to admission is 7 days and takes a median of additional 3.5 days until ICU admission. In order to maximize the benefit of acute HIF stabilization and observe a potential benefit, a dose of 900 mg once daily for 14 days is recommended.

Vadadustat doses of 150-1200 mg per day were tested in healthy volunteers and patients with anemia secondary to chronic kidney disease (CKD). A dose of vadadustat 900 mg was administered to healthy volunteers and patients with CKD for 10 days without significant adverse effects. In healthy volunteers, the most commonly reported AEs (nausea, diarrhea, abdominal pain, flatulence, dyspepsia, headache, and dizziness) were generally reported to be mild to moderate in severity. There were no SAEs, deaths, or withdrawals due to AEs, and no clinically relevant changes in mean laboratory values, vital signs or ECGs. In CKD subjects, the most commonly reported AEs were nausea, diarrhea, vomiting, hypertension, hypotension, coronary artery disease, chronic and acute renal failure, gastroenteritis, urinary tract infection, and pneumonia.

8. Study Procedures

8.1. Recruitment

All COVID-19 patients will be screened for eligibility. If a patient meets screening eligibility, the study coordinator will introduce the study to the patient to determine interest. If the patient is interested in participating in the study, the study coordinator will introduce the patient to one of the study clinicians either in person or virtually utilizing WebEx. Written consent will be obtained for all patients interested in study participation. Patients will be randomized if meeting all eligibility criteria (as specified in inclusion/exclusion criteria). Initial recruitment will occur at MHHSouthwest and MHH-Texas Medical Center for the first 30 patients. After 30 and then 120 patients are treated, screening and enrollment will be suspended (suspension of enrollment will occur at n = 30, based on DSMB recommendation, enrollment will continue during the safety analysis at n = 120) to enable an interim blinded safety analyses by the DSMB. The DSMB will provide a report to the UTHealth COVID-19 Research Workgroup and UTHealth IRB to consider further patient screening and enrollment.

8.2. Concomitant medication

Probenecid is not allowed as it may increase systemic exposure to vadadustat. Investigators should adjust the dose of these concomitant medication as follows:

1. Simvastatin, maximum daily dose of 20 mg
2. Rosuvastatin, maximum daily dose of 10 mg

If the patient is currently taking erythropoiesis-stimulating agents or ESA (e.g., epoetin alfa, darbepoietin alpha, or methoxy polyethylene glycol-epoetin beta), the medication should be discontinued.

Sulfasalazine and other breast cancer resistance protein (BCRP) substrates (imatinib, gefitinib, nilotinib, prazosin, glyburide, cimetidine, AZT and lamivudine) should be used with caution as vadadustat can increase systemic exposure to these medications. Lopinavir and rotinavir are also prohibited due to observed higher LFTs in patients treated with these drugs.

Phosphate binders (iron-containing and non-iron containing) and oral iron supplements should be taken at least 2 hours before or after the dose of vadadustat as they can interfere with vadadustat absorption.

At enrollment, the study team will notify the treating physician of medications prohibited by the study. Study personnel will review the patient's current medications daily to identify the use of any prohibited medications and notify the treating physician when they are prescribed. The study team will discuss the use of alternative medications. If the prohibited medication cannot be stopped the patient will continue in the study but study medication will be stopped.

8.3. Complete Withdrawal from Study Visits/Assessments

A patient has the right to withdraw consent for participation in the study at any time. Withdrawal of consent is a patient's refusal to continue study treatment, data collection and ALL methods of follow-up noted in the informed consent form: procedures, participation in reduced procedures/study visits, telephone contact only or alternative contact only, source document or designated alternative contact, or access to medical records from alternative sources.

It is important for the Investigator to review options with a patient. The patient may decide to stop study medication but still be followed up in the hospital and contacted on Day 7, 14, and 28 after discharge to assess safety and efficacy outcomes. Alternatively, the patient may decide to completely withdraw consent from the study which entails not receiving study medication, not to be followed up in the hospital, and not to be contacted after discharge.

8.4. Data Collection

Refer to *Appendix 1 (Schedule of Activities)* for detailed timing and frequency of the below activities:

- Informed consent
- Demographic and contact information
- Oxygen saturation by pulse oximetry
- Pregnancy test (collected only at baseline)
-
- Hemoglobin, creatinine, liver function test (AST, ALT, Bilirubin) at baseline, day 7, day 14 and during long term follow up visits at 6mo, 12mo and 24mo (If the patient remains hospitalized after 14 days, or the drug is discontinued for any reason, creatinine and hemoglobin lab values will be collected for up to 2 days following the last dose, again ceasing upon discharge.)

- Height, weight, BMI
- Eligibility, at baseline
- Randomization
- Blood biomarkers (during hospitalization only) ○ Baseline, Day 7 and Day 14 (10 cc each collection)
- Urine biomarkers (during hospitalization only) ○ Baseline, Day 7 and Day 14 (5 cc each collection)
- Medical/Medication history
- Study medication dosing
- AE and SAEs
- Concomitant medications and COVID-19 specific treatment other than vitamins and minerals
- Modified SOFA, daily until discharge, & at Day 7, 14, and 28, with discharged patients receiving a score of 0 and deceased patients receiving a score of 20.
- NIAID-OS, daily and until discharge and at Day 7, 14, and 28
- For patients who are discharged, before or after completing 14 days of in-hospital treatment, a phone visit at day 7, 14, and 28 after randomization will be conducted to collect serious adverse events and NIAID-OS score.

8.4.1. Extended Follow Up

Eligible patients will be asked on the consent form if they would like to consent to participate in an extended follow up opportunity which will consist of the following:

- Return to the clinic at 6, 12 and 24 months
- Complete a quality of life questionnaire
- Provide a small blood sample (10 ml)
- Provide a urine sample (10 ml)
- Perform a pulmonary function test to see how well their lungs are working and a bedside echocardiogram to see how their heart is working.

The participant will receive a $100 gift card for each follow up visit completed. In addition, the parking and travel expenses will be covered.

To consent to the optional long term follow up, the patient will be asked to check a box and initial.

8.5 Protocol Deviations: Protocol deviations will be recorded in the RedCAP database. The type of deviation will be specified. The deviation will be described and corrective actions will be recorded.

9. Data and Safety Monitoring Board (DSMB)

A DSMB will be formed. DSMB is a group of external experts who will provide oversight to the conduct of the study and provide safety assessment through planned interim analysis. The DSMB

shall include an odd number of members, minimum of 3, preferably 5. It should include at least one of the following: one critical care physician, one physician-trialist and one statistician.

In addition to the ongoing and final safety assessments through the course of the trial, a detailed review of safety by the DSMB will follow the ascertainment of the 14-day safety on the first 30,120 and 400 patients. After 30,120 and 400 patients have received treatment and have completed their day 14 event, an interim blinded safety analysis by the DSMB will be completed. The DSMB will provide a report to the UTHealth COVID-19 Research Workgroup and UTHealth IRB to consider further patient screening and enrollment. In the n=400 safety analysis there will be no planned suspension in ongoing recruitment during evaluation of the findings.

10. Discontinuation of study treatment in individual patients (Stopping Criteria)
Vadadustat must be permanently discontinued if a patient meets one of the following pre-specified criteria:

1.	Patient completes 14 days of in-hospital treatment since randomization
2.	Patient is discharged before 14 days of treatment since randomization
3.	If the clinical team deems necessary that the patient receive a medication that was previously prohibited, or if the clinical team decides the patient should not receive vadadustat.
4.	Persistent eGFR decrease (greater than 2 days ≤ 30)
5.	Hemoglobin above the gender specific upper limit of normal (ULN): females (16 g/dL) and males (18 g/dL)
6.	Patient dies

11. Safety Assessment
11.1. Adverse Events (AEs)
For the purposes of this study, an AE is any untoward medical occurrence (including an abnormal laboratory finding) that occurs in the protocol-specified AE reporting period; the event does not necessarily have a causal relationship with that treatment or usage.

An AE includes medical conditions, signs, and symptoms not previously observed in the subject that emerge during the protocol-specified AE reporting period, including signs or symptoms associated with pre-existing underlying conditions that were not present prior to the AE reporting period.
AEs therefore include the following:

- All AEs, whether suspected to be causally related to study drug or otherwise.
- All AEs secondary to any medication overdose, medication error, abuse, withdrawal, sensitivity, or toxicity.
- Illnesses apparently unrelated to study drug, including the worsening of a pre-existing illness (see paragraph below on Pre-existing Conditions).

- Injury or accidents. Note that if a medical condition is known to have caused the injury or accident (for example, a fall secondary to dizziness), the medical condition (dizziness) and the accident (fall) will be reported as 2 separate AEs.
- Abnormalities in physiological testing or physical examination findings that require clinical intervention or further investigation (beyond ordering a repeat [confirmatory] test).
- Laboratory abnormalities that require clinical intervention or further investigation (beyond ordering a repeat [confirmatory] test) unless they are associated with an already reported clinical event. Laboratory abnormalities associated with a clinical event reported as an AE (for example, elevated liver enzymes in a subject with jaundice) will be described under 'Comments' on the report of the clinical event rather than reported as separate AEs.

The following guidelines are to be used when reporting AEs for this study:

- **Medical Diagnoses** – Whenever possible, a medical diagnosis term will be used to report AEs instead of signs and symptoms due to a common etiology, as determined by qualified medical study staff. For example, pneumonia will be the reported AE term, instead of fever, dyspnea, etc., when the diagnosis has been established. Signs and symptoms will be reported as event terms only when the medical diagnosis remains unknown and revised to a medical diagnosis term once it has been established.
- **Procedures** – Diagnostic and therapeutic noninvasive and invasive procedures, such as surgery, will not be reported as AEs. However, the medical condition for which the procedure was performed will be reported if it meets the definition of an AE. For example, an acute appendicitis that begins during the AE reporting period will be reported as the AE and the resulting appendectomy noted under "Comments."
- **Preplanned therapeutic procedures** not associated with a new medical condition or worsening pre-existing condition will not be reported as AEs.
- **Pre-existing Conditions** – In this study, a pre-existing condition (i.e., a disorder present before the AE reporting period started and noted on the pretreatment medical history/physical examination form) will not be reported as an AE unless the condition worsens, or episodes increase in frequency during the AE reporting period.
- **Abnormal Test Findings** – All laboratory test results will be reviewed by the research team. The research team will utilize his/her judgment in determining if out of range laboratory values are clinically significant. Laboratory tests that are considered clinically significant will be reported as AEs, either separately or as part of a description of a symptomatic AE. If there are significant changes in a laboratory report from a previous result that are determined to be clinically significant, these will also be reported as AEs. Any abnormal laboratory value, which requires treatment or further diagnostic testing or results in discontinuation from study will be reported as AEs. An expected laboratory abnormality from a condition that is part of the medical history is not considered clinically significant for the purposes of the study unless it represents a worsening of the condition.

Special daily safety monitoring for the risk of thrombosis and proinflammatory state is planned in this trial. This will be achieved by daily review of electronic medical records for clinical evaluation

of thromboembolic events. Furthermore, all patients will be managed according to local institutional anticoagulation protocols.

Thrombo-embolic events (complications) including:

- Deep vein thrombosis—catheter associated or spontaneous
- Pulmonary embolism
- Ischemic stroke
- Systemic arterial thrombosis
- Acute coronary syndrome/myocardial infarction.

11.2. Serious adverse events (SAEs)

SAEs are AEs that meet one or more of the following criteria/outcomes:

- Death
- Life-threatening
- In-patient hospitalization or prolongation of existing hospitalization
- Persistent or significant disability/incapacity
- Congenital anomaly/birth defect
- Is considered a medically important event not meeting the above criteria, but which may jeopardize a patient, or may require medical or surgical intervention to prevent one of the criteria listed in this definition

Serious also includes any other event that the investigator judges to be serious. If there is any doubt whether the information constitutes an AE or SAE, the information is to be treated as an SAE.

Life threatening – Defined as any event in which the subject was at risk of death at the time of the event. 'Life threatening' does not refer to an event which hypothetically might have caused death if it were more severe. For example, drug-induced hepatitis that resolved without evidence of hepatic failure would not be considered life threatening, even though drug-induced hepatitis of a more severe nature can be fatal.

Hospitalization – Defined as an overnight admission with observation of a minimum of 24 hours. A hospitalization planned before the start of the study for a preexisting condition that has not worsened during the AE reporting period does not constitute an SAE unless an untoward event occurs related to the procedure (e.g., elective hospitalization for a total knee replacement due to a pre-existing condition of osteoarthritis of the knee that has not worsened during the study).

Disability – Defined as a substantial disruption in a person's ability to conduct normal life functions.

In addition to the above criteria for classifying AEs as serious, the following situations will be also be classified as serious for purposes of this study:

Malignancies – If a subject develops basal cell carcinoma of skin, squamous cell carcinoma of skin, or cervical carcinoma in situ during the study, or has worsening of these events from baseline, the investigator will determine if the event is reported as an AE or SAE. Newly diagnosed malignancies or a recurrence of a malignancy will be reported as an SAE with the seriousness criterion “medically important” if no other seriousness criteria are met.

All cardiac events will be specifically elicited and followed. Troponins ordered by the clinical team will be recorded in the Daily lab section of the CRF. Troponin levels are not required, but will be recorded when available. Recent data using vadadustat in the PROTECT2 study showed an increase rate of major adverse cardiac events (MACE) in some study participants. This increase in MACE events occurred in patients who were not on dialysis, were treated outside the US and were treated for more than 6 months with vadadustat. This risk as not seen in dialysis patients and was not seen in study participants in the US. The DSMB was notified of these results. Based on the above data, the DSMB recommended that all cardiac events be tracked and troponins be recorded when ordered by the treating team.

Any SAE, regardless of causal relationship, must be reported to the sponsor within 24 hours after the Investigator becomes aware of the SAE. Compliance with this time requirement is essential so that sponsor may comply with its regulatory obligations.

11.3. Eliciting Adverse Event Information

The investigator is to report all directly observed AEs and all AEs spontaneously reported by the patient. Patients will be questioned daily about AEs if they are able to respond, and then followed through the duration of the study.

11.4. Reporting

Each AE is to be classified by the investigator as serious or non-serious.

All AEs that occur in study subjects during the AE reporting period specified in the protocol must be reported, whether or not the event is considered to be related to study drug.

11.4.1. Reporting Period

The AE reporting period for this study begins upon initiation of study drug and ends up to 16 days after randomization (14 days of drug dosage plus two days), while SAEs will be continued to be reported up to Day 28, and will be assessed via follow-up, or through the patient’s chart. However, AEs considered secondary to protocol mandated procedures should be reported post signing of the informed consent form (ICF), whether or not they occur before or after initiation of study drug. Subjects discharged from the hospital will be contacted on day 7, 14, and 28 to assess for SAEs. In addition, SAEs that occur during the protocol-defined AE reporting period that the investigator assesses as unexplained and related to the study drug will also be reported as an SAE to the UTHealth IRB and the FDA. AEs and SAEs that occur after the protocol-defined period that the investigator assesses as related to the study drug will also be reported as an AE to UTHealth. If the

event is serious, related, and unexpected, the event will be reported to the investigator IRB and the FDA.

11.4.2. Reporting AEs

All AEs (serious and non-serious) are to be reported on the AE CRF in the clinical database during the AE reporting period as described in Section 11.1.

11.4.3. Reporting SAEs

Any SAE, regardless of causal relationship, will be reported to the sponsor's designee within 24 hours after the investigator becomes aware of the SAE. Compliance with this time requirement is essential so that the sponsor may comply with its regulatory obligations. Additionally, it may be necessary for UTHealth (or designee) to directly communicate with the investigator if additional information is required.

The initial SAE report should be completed as fully as possible but will contain, at a minimum item number 1 to 6:

1. Subject number/ID, sex, and age
2. The date of report
3. Name of the reporter
4. Name of the suspected medicinal product
5. A description of the event, including event term(s), seriousness criteria, and a clinical summary of the event
6. Causality assessment

The investigator will assess the relationship to each specific component of the study treatment. A SAE Report Form will be sent to the sponsor or its designee via email or the investigator will call the sponsor or its designee SAE hotline within 24 hours of being made aware of the SAE.

Refer to the SAE Report Form and SAE Report Form Guidelines for more details on SAE reporting, including timelines, and email for reporting and the contact information for any questions regarding SAE reporting.

The investigator must report follow-up information relating to an SAE to the study safety monitor as it becomes available by submitting a REDCAP SAE Report Form. The subject will be observed and monitored carefully until the condition is resolved or stabilizes.

All deaths are to be thoroughly investigated and reported to the UTHealth IRB. Autopsy reports and death certificates are to be obtained, if possible. In-hospital deaths will be investigated via electronic medical records (EMR). Out-of-hospital deaths will be investigated by calling their family, and reviewing available death certificates.

In addition to the sponsor's responsibility for reporting SAEs to all applicable regulatory agencies, Institutional Review Board (IRB) or Independent Ethics Committee (IEC), and investigators within

the required timeline, UTHealth and/or its designee are responsible for reporting SAEs to all applicable regulatory agencies, central ethics committees, and investigators as well within the required timeline.

11.5. Relationship to Study Drug

The investigator must provide an assessment of causality (relationship to study drugs) at the time of the report. If causality is difficult to determine, for example, due to incomplete clinical information at the time of the report, the investigator must use clinical judgment under consideration of the criteria outlined below and provide a causality assessment. It is also good practice to include a rationale for the causality assessment.

The assessment of causal relationship to study drug will be evidence-based, and not based on the premise that all AEs are possibly causally related to study drug until proven otherwise.

Examples of evidence that would suggest a causal relationship between the study drug and the AE include the occurrence of an AE that is uncommon and known to be strongly associated with drug exposure (e.g., angioedema, hepatic injury, Stevens-Johnson Syndrome), or an AE that is uncommon in the population exposed to the drug.

The site investigator, on the basis of his or her clinical judgment and the following definitions, determines the relationship of the AE to the protocol intervention as one of the following:

Algorithm to Determine Relatedness of Adverse Event in Study Agent	
Unrelated	The temporal relationship between treatment exposure and the adverse even is unreasonable or incompatible and/or adverse event is clearly due to extraneous causes (e.g. underlying disease, environment).
Unlikely	Must have both of the following two (2) conditions, but may have reasonable or only tenuous temporal relationship to intervention:<br>1. Could readily have been produced by the subject's clinical state, environmental, or other interventions.<br>2. Does not follow known pattern of response to intervention.
Reasonable Possibility	Must have at least two (2) of the following three (3) conditions:<br>1. Has a reasonable temporal relationship to intervention<br>2. Could not have readily been produced by the subject's clinical state or environmental or other interventions<br>3. Follows a known pattern of response to intervention.
Definitely	Must have all three (3) of the following conditions:<br>1. Has a reasonable temporal relationship to intervention 2. Could not possibly have been produced by the subject's clinical state or have been due to environmental or other interventions<br>3. Follows knows pattern of response to intervention.

Default assessments using the 'related' category without supportive evidence for a causal relationship to study drug is generally uninformative and does not contribute meaningfully to the development of the safety profile of the drug or to subject protection.

Investigators are encouraged to choose the most plausible cause for the event(s) from the following list: medical history, lack of efficacy/worsening of treated condition, study treatment, other treatment (concomitant, or previous), withdrawal of study treatment, administration error, protocol-related procedure, others (specify).

11.6. Severity

The investigator will assess each AE using the CTCAE guidelines to describe the maximum severity of the AE:

- **Grade 1 Mild** – asymptomatic or mild symptoms; clinical or diagnostic observations only; intervention not indicated.
- **Grade 2 Moderate** – minimal, local or noninvasive intervention indicated; limiting age appropriate instrumental ADL.
- **Grade 3 Severe or medically significant but not immediately life threatening** – hospitalization or prolongation of hospitalization indicated; disabling; limiting self care ADL.
- **Grade 4 Life threatening consequences** – urgent intervention indicated.
- **Grade 5 Death related to AE**.

Note that a severe AE is not necessarily a serious AE. For example, a headache may be severe in intensity, but would not be classified as serious unless it met one of the criteria for serious events listed above, see Section 11.4.3.

11.7. Emergency Unblinding:

A representative of the study leadership will always be available using 24-hour telephone number for communication with investigators and healthcare providers caring for the patient.

If a clinician requests emergency unblinding of study drug in response to a clinical event, the study representative will discuss the clinical utility of unblinding with the clinician. Emergency unblinding will only occur if the clinical and investigator agree that it is necessary to treat the patient. Emergency unblinding should be rare as there is no antidote to the drug, and it is given orally. All requests for emergency unblinding will be reported to the study safety officer. The safety officer will then determine if the patient should continue to receive study medication.

11.7.1 Non-Emergency Un-blinding

At times the sponsor or designee may request un-blinding of safety events in accordance with their reporting responsibilities. In these cases, if the treatment team does not need to be un-blinded, the database manager will un-blind the sponsor or their designee but will not un-blind the clinical or research team.

11.8 Follow-up of Unresolved Events

All AEs will be followed until they are resolved, or the investigator assesses them as chronic or stable or the subject's participation in the study ends (i.e., until a final report is completed for that subject).

All SAEs and non-serious events that are ongoing at the time a subject's participation in the study ends and that are assessed by the investigator as related to the study drug, will continue to be followed until they resolve or until the investigator assesses them as "chronic" or "stable". Follow-up information will be reported to UTHealth on the appropriate CRF or paper SAE Report Form if EDC is unavailable.

More details of the study safety plan are described in the study Manual of Procedures.

12. Sample Size and Power Calculation

The trial will investigate the superiority of vadadustat 900 mg per day to placebo for the prevention and treatment of ARDS among COVID-19 patients, defined by the proportion of patients at day 14 who are dead (8) or hospitalized, on invasive mechanical ventilation or ECMO (7) or hospitalized, on noninvasive ventilation or high flow oxygen devices (6) on the NIAID-OS (i.e. decrease in the proportion of participants with NIAID-OS $\leq$ 6 at day 14).

Anticipated sample size for the current study is N = 650. An evaluation by the DSMB will occur following ascertainment of 14-day safety outcomes for the first 30 patients that are randomized and dosed, and a second at the first 120 (i.e. n = 60 receiving Vadadustat).

Bayesian analysis for superiority will occur following ascertainment of outcome at fourteen days after randomization of N = 650 patients. We stipulate that a posterior probability of a > 2.5% decrease in the proportion of NIAID-OS $\geq$ 6 as a function of treatment that is > 0.85 constitutes sufficient evidence to declare that the active treatment condition is superior to placebo. The prior distribution for analysis will be ~Beta(a= 1, b = 1), or in the presence of covarying for stratification factors ~Normal (μ=0, σ2=1 x 10) for the coefficients in the log-form of the appropriate generalized linear model.

We anticipate a decrease in the proportion of NIAID-OS $\geq$ 6 participants due to treatment will be approximately 15%, with preliminary data suggesting a rate of 25.1% in the placebo condition. Assuming this effect, K = 1000 Monte Carlo clinical trial simulations, for N = 650 participants and the previously stated decision rules resulted in detection of a > 2.5% decrease in NIAID-OS $\geq$ 6 due to the active treatment 99.1% of the time with a Type I Error rate of 3.5%. For more modest effects, 12.5% and 10% decreases in NIAID-OS $\geq$ 6, the current sample and decision rules will lead to detection 96.1% and 86.3% of the time with Type I Error rates of 4.8% and 3.7% respectively.

13. Statistical Analysis

13.1. Specific Statistical Hypothesis Primary Outcome

Among adult hospital admissions with lab-confirmed diagnosis of COVID-19, vadadustat 900 mg will demonstrate superiority to placebo as defined by a lower probability, at treatment day 14, of death (8) or hospitalization, on invasive mechanical ventilation or ECMO (7) or hospitalization, on non-invasive ventilation or high flow oxygen devices (6) on the NIAID-OS. Generalized linear modeling will evaluate the probability of patients demonstrating this status at day fourteen of treatment as a function of treatment condition after covarying for stratification variables.

Secondary Outcome

Among adult hospital admissions with lab-confirmed diagnosis of COVID-19, vadadustat 900 mg will demonstrate superiority to placebo as defined by a higher probability, at treatment day 14, of recovery on the MSOFA scale (MSOFA = 0). Generalized linear modeling will evaluate the probability of patients demonstrating an MSOFA = 0 at day fourteen of treatment as a function of treatment condition after covarying for stratification variables.

13.2. Bayesian Modeling Strategy

Initial analyses examining group differences for baseline variables will use cross-tabulation, ANOVA's, and examination of correlations between baseline variables and specified outcomes. For the purposes of evaluating the comparability of groups, a posterior probability of > 95% will constitute evidence for statistically reliable differences. Baseline or demographic variables on which group differences are detected, and which are correlated with outcomes, meet the definition of confounders (Assmann et al 2005, Pocock et al 2002) and will result in two sets of analyses: one in which the relevant variable is included as a covariate and one in which it is not. This will permit determination of the degree to which any group differences might confound conclusions regarding treatment.

Broadly, the data analytic strategy will use generalized linear and multilevel models (R v. 3.6, and Stan 2.17) for both discrete and continuous outcomes (SAS Institute 2002-2012, R Core Team 2017, Carpenter et al 2017). Multilevel generalized linear modeling to account for clustering of patients within site and repeated observations within patients will evaluate continuous, dichotomous, and count data. All analyses will apply intention-to-treat principles with missingness addressed via joint modeling of observed outcomes and the missing data, an approach robust to ignorable missingness (i.e., MCAR and MAR) (Best & Thomas 2000). Sensitivity analyses will evaluate robustness of analytic conclusions to missing data. Non-ignorable missing data patterns will be addressed through pattern-mixture modeling methods (Fitzmaurice & Laird 2000). Evaluation of posterior distributions will permit statements regarding the probability that effects of varying magnitudes exist, given the data. Specification of diffuse, neutral priors will reflect the initial uncertainty regarding effect sizes. For all generalized linear models, priors for regression coefficients will be specified as ~Normal (μ=0, σ2=1 x 10), level one and two error variances will be specified as ~HalfNormal (μ=0, σ2=1 x 10^3). The choice of prior distribution for level two variances will follow

Gelman's recommendations (Gelman 2006). Priors for the comparison of proportions will be specified as ~Beta (α=1.0, β=1.0).

14. Ethics

The protocol titled "Vadadustat for the prevention and treatment of acute respiratory distress syndrome (ARDS) in hospitalized patients with Coronavirus Disease 2019 (COVID-19) will be submitted to the local Institutional Review Board (IRB).

The protocol will be reviewed by:

The Committee for the Protection of Human Subjects
The University of Texas Health Science Center at Houston
University of Texas Professional Building
6410 Fannin Street, Suite 1100
Houston, Texas 77030
Institutional Official: Anne Dougherty, MD FWA# 00000667

IRB Registration Numbers: IRB00000308, IRB00003763, IRB00004604, IRB00008445

The study will be reviewed, approved and conducted in accordance with regulations mandated by The University of Texas Health Science Center at Houston Institutional Review Board. In addition, the study will be conducted according to the College of American Pathologists standards that meet Good Clinical Practice (GCP). These standards respect the following guidelines:

- GCP: Consolidated Guideline (International Conference on Harmonization ICH of Technical Requirements for the Registration of Pharmaceuticals for Human Use, May 1996)
- Integrated Addendum to ICH E6 (R1): Guideline for Good Clinical Practice, E6 (R2); Current step 4 version dated 9 November 2016.
- United States (US) Code of Federal Regulations (CFR) dealing with clinical studies (21 CFR Parts 50, 54, 56, 312, 314, and 511)
- Declaration of Helsinki, concerning medical research in humans ("Recommendations Guiding Physicians in Biomedical Research Involving Human Subjects,") Helsinki 1964, amended Tokyo 1975, Venice 1983, Hong Kong 1989 and revised version of Somerset West, Republic of South Africa, October, 1996, Note of Clarification added by the WMA World Medical Association General Assembly, Washington 2002, Note of Clarification added by the WMA General Assembly, Tokyo 2004

15. Informed Consent

Potential subjects are to receive a thorough explanation of study details and procedures and provided time to ask any questions. All subjects must sign the IRB approved informed consent form before performing any study-related activity (including screening activities). Due to COVID-19 to prevent cross contamination, the original signed consent form is given to the patient. A photograph using HIPAA compliant Tiger Connect will be captured of the signature page and will be placed in the subject's study file. In the case where an adult is incapable of making an informed decision, they will be excluded from study participation.

16. Data handling and record keeping

16.1. CRFs/EDC

This study will utilize an electronic data capture (EDC) system to manage data collection during this trial. The system is fully Code of Federal Regulations 21 part 11 compliant. This EDC will reside behind the Zone 100 UTHealth firewall with Shibboleth-based user-access and identification. Patients will be assigned a unique ID number. A master key file will be stored on the shared password protected drive at UTHealth. Only Co-investigators at UTHealth and their staff (to be added through amendment at a later date when hired) will have access to this master key file.

The EDC in this study will utilize the REDcap system maintained on secure UTHealth servers. This database possesses a graphical user interface to facilitate data entry, capability of tracking data management and user activity, a data validation element to check user data (e.g. impermissible/outof-range or missing values), and a reporting function to assist with the review and analysis of data. The data base will utilize a relational structure to avoid so-called "orphan records" with staff password protected and access to and modification of data tables restricted based on their operational roles. Clinical Research Forms (CRF's) available through this system are required and will be completed for each randomized subject. Any data from the electronic system are the sole property of the sponsor and will not be made available in any form to third parties, except for authorized representatives of the sponsor or appropriate regulatory authorities, without written permission from the sponsor. The investigator has ultimate responsibility for the accuracy, authenticity, and timely collection and reporting of all clinical, safety, and laboratory data entered in the EDC or any other data collection forms. The CRFs will be signed electronically by the investigator to attest that the data contained on the CRFs are true.

In most cases, the source documents are contained in the subject's chart at the hospital, dialysis or research clinic, or the physician's office. In these cases, data collected on the CRFs will match the data in those charts.

16.2. Record Retention

To enable evaluations and/or audits from regulatory authorities or the sponsor, the investigator agrees to keep records, including the identity of all participating subjects (sufficient information to link records, e.g., CRFs and hospital records), all original signed informed consent forms, copies of all CRFs, SAE forms, source documents, detailed records of drug disposition, and adequate documentation of relevant correspondence (e.g., letters, meeting minutes, and telephone calls reports). The records will be retained by the investigator according to the International Conference of/Council for Harmonisation (ICH) of Technical Requires for Pharmaceuticals for Human Use local regulations, or as specified in the Clinical Study Agreement, whichever is longer.

If the investigator becomes unable for any reason to continue to retain study records for the required period (e.g., retirement and relocation), the sponsor will be prospectively notified. The study records will be transferred to a designee acceptable to the sponsor, such as another investigator, another institution, or to the sponsor. The investigator will obtain sponsor's written permission before disposing of any records, even if retention requirements have been met.

17. Quality control and assurance

17.1. Manufacture and Storage

Drug to be tested: Vadadustat is manufactured by Akebia and contains microcrystalline cellulose (MCC), sodium starch glycolate, hydroxypropyl methylcellulose (HPMC), colloidal silicon dioxide, and magnesium stearate, and a nonfunctional film coat in addition to vadadustat. Placebo: Placebo is manufactured by Akebia and contains MCC, colloidal silicon dioxide, magnesium stearate, and a nonfunctional film coat. Vadadustat and placebo are stored at room temperature.

17.2. Active Dose

Subjects will receive 900 mg as 6 x 150 mg tablets once daily.

17.3. Dispense and Handling of Study Drug

The research pharmacist or designee will dispense 6 tablets of vadadustat or placebo daily for each study patient. Administration to the study patients will follow the local institution's procedures.

18. Protocol Amendment

18.1 Long-Term Follow-up

The purpose of this amendment is to characterize the long-term effects on COVID-19 survivors who were hospitalized with SARS-CoV-2 and treated with either a HIF activator vadadustat or placebo. Specifically, we will 1) characterize the occurrence of long-term COVID-19 sequelae in a well-described patient cohort and 2) address the hypothesis that vadadustat would attenuate or prevent the long-term disease sequelae of SARS-CoV-2.

18.2 Study Objectives.

Primary: Evaluation of pulmonary capacity as a function of treatment, time and the interaction of treatment and time.

Secondary: Evaluate cardiac, hepatic and renal outcomes, quality of life, and long-term neurocognitive impairment of COVID-19 patients treated with either vadadustat or placebo

18.3 Study Diagram.

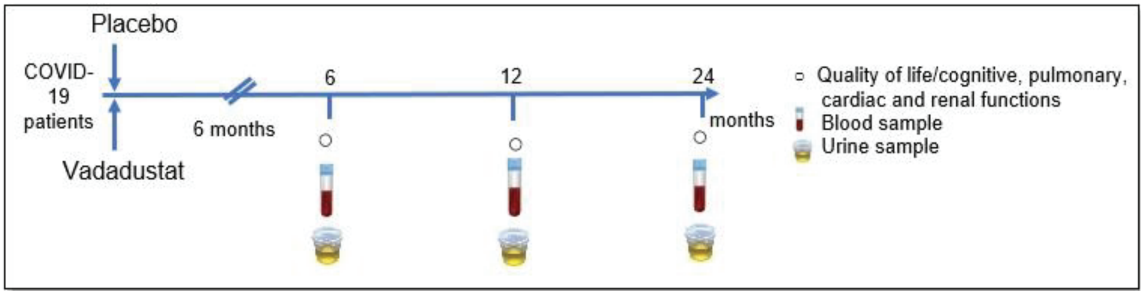

18.4 Study Population.

Eligibility Criteria

18.4.1 Inclusion Criteria

- Male or Female age ≥ 18.
- Previously enrolled in the vadadustat study and agreed for an extended follow-up.
- Patients who Speak/Write in English or Spanish

18.4.2 Exclusion Criteria

- Patients who were enrolled in the vadadustat study but withdrew before taking the 1st dose of the study drug.
- Patients who were enrolled in the vadadustat study but did not consent for an extended follow-up.
- Patients who do not speak English or Spanish.

18.5 Data Collection.

The following will be collected on the 6th, 12th and 24th month follow-up.

- Informed consent for the extended follow-up shall be referenced to the IRB approved ICF of vadadustat study starting February 5, 2021 wherein patients have agreed to participate in this long-term study.
  For those patients consented prior to February 5, 2021, please refer to Section 18.9 of the study protocol for the reconsenting process.
- Body Mass Index, height, weight
- Medication History
- Tobacco use
- Past Medical History
- Laboratory Tests: ALT, AST, Total Bilirubin, Creatinine, and Hemoglobin.
- Cardiac, Renal and Lung Biomarkers (Blood and urine)
  Pulmonary Function test
- Transthoracic echocardiography (TTE)
- Lung ultrasound
- PROMIS Scale v1.2 Global Health Survey MoCA Test (Montreal Cognitive Assessment)

18.6 Study Procedures.

Each study participant will undergo the following procedures on 6th, 12 and 24th month follow-up. There is a one (1) month window to complete each follow up appointment once a patient reaches their long term follow up due date. This one-month window provides flexibility to schedule and accommodate both the patient and the clinical teams' availability. If a patient cannot be scheduled

within one month of their follow up date, this will be deemed a loss to follow up and documented in RedCAP accordingly.

18.6. 1 Sample collection processing

Blood and urine samples will be collected at our Clinical Research Unit (CRU) and processed using standard protocols. Blood specimens will be collected from the patient (biomarkers and laboratory specific tests) and urine over the course of the visit. Universal precautions and institutional biosafety procedures will be followed.

18.6.2 Transthoracic Echocardiography (TTE)

This test is the primary noninvasive imaging modality for quantitative and qualitative evaluation of cardiac anatomy and function using an ultrasound. This test will be performed by physicians, fellowship-trained in ultrasonography.

The study currently evaluates pulmonary function and cardiac function as part of the long term follow up protocol. There is currently no lung imagining as part of the follow up process. Previous literature has shown ultrasound is as accurate as computed tomography for interstitial lung disease, without the cost or ionizing radiation. We are adding lung ultrasound as a seamless addition to the echo portion of the long term follow up study. We anticipate this exam will add approximately five minutes to the ultrasound portion of the protocol. There are no known harms associated with lung ultrasound.

C60 probe using abdominal preset. Axial images obtained via cranial caudal sweep of the right and left hemi-thorax in the mid-clavicular, mid-axillary, and peri-scapular. De-identified video clips of each location will be saved. We will make notation of any unusual signature with particular attention to previously described findings in COVID lung disease including B lines and sub-pleural consolidations.

Data Collection Sheet:

	R Midclav	L Midclav	R Midax	L Midax	R Periscap	L Periscap
3+ B lines						
Confluent B Lines						
Sub-pleural Conolidation						
Pleural Effusion						

Check mark indicates present of the finding

Other findings:

18.6.3 Pulmonary Function Tests

Participants will be subjected to a pulmonary testing using spirometry. Briefly, spirometry is used to measure forced expiratory flow rates and volumes. It is the most commonly used pulmonary function test and is useful in the evaluation of patients with respiratory symptoms (e.g., dyspnea, cough, wheeze) or risk factors for respiratory diseases. Specifically, we will be measuring FVC, FEV, FEV/FVC ratio.

18.6.4 Study Questionnaires

The PROMIS Global Health assess an individual's physical, mental and social health. The measures are generic, rather than disease-specific, and often use an 'In General' item context as it is intended to globally reflect individuals' assessment of their health. The adult PROMIS Global Health measure produces two scores: physical health and mental health. The Montreal Cognitive Assessment (MoCA) is a tool to assess short memory, visuospatial abilities, executive functions, attention, concentration and working memory, language and orientation to time and place.

18.6.5 Visit Compensation

Each time a patient completes follow up, she/he will receive a $100 gift card, (up to a maximum of $300 for completion of 3 follow-up in 24 months). Patients will be provided with a $100 SPA Card at the end of each visit. Additionally, the study team will arrange round trip transportation of the patient from and to their home or destination. Should they choose to drive, the research team will provide patients with parking validation.

18.6.6 Study Dissemination

Study clinicians will review any results found outside of expected ranges for the Transthoracic Echocardiography, pulmonary function test, lung ultrasound, labs and/or MoCA. Participants will be notified via mail to follow up with their PCP.

18.7 Withdrawal from Follow-up Visits

A patient can withdraw from participating in the long-term follow-up at any point for any reason. If possible, reasons for discontinuation or refusal will be documented.

18.8 Statistics

18.8.1 Specific Statistical Hypothesis of Primary Outcome

The primary outcome analysis will model pulmonary capacity as a function of time, treatment and the interaction of time and treatment after controlling for stratification variables (i.e. site and MSOFA < 4 or ≥ 4).

18.8.2 Sample Size and Power Calculation

For the purpose of sample size justification, we assume a small between-groups effect (Cohen's d = 0.2) unfolding in linear fashion over time (6, 12, and 24 months). Estimation will focus on the interaction of treatment and time to ascertain the degree to which the two group's trajectories of change differ. We further assume that pulmonary function will exhibit with subject autocorrelation of r = 0.3 for every six-month time lag with this correlation diminishing by 50% for every additional six-month time lag. We anticipate enrolling N = 200 participants, and assume attrition will be approximately 10% resulting in a final sample of n = 180. Stipulating a posterior probability of 0.80 for the existence of an interaction effect (i.e. there is an 80% chance that there are differential trajectories of change for the treatments, K = 1000 Monte Carlo simulations identify an interaction term 92% of the time.

18.8.3 Linear Multilevel Models

Analyses will focus on longitudinal trajectories of change for outcomes specified in objectives 1-3. Generalized linear multilevel models will evaluate change as a function of time, treatment, and the interaction of treatment and time after controlling for stratification variables (i.e. site and baseline severity as measured by the MSOFA < 4 or $\geq$ 4). Analyses will utilize Bayesian inference. Prior distributions will be neutral (centered on the null hypothesis) and weakly informative as described by Gabry and Goodrich (2020). This will have the benefit of regularizing any parameter estimates and render more conservative estimates of effect size. Regarding priors essentially as a set of assumptions, we will evaluate the robustness of our analytic conclusions to alternative prior specifications with varying degrees of regularization/skepticism (Gelman, 2007; Spielgelhalter, 2004).

18.9 Informed Consent

Patients enrolled in the vadadustat study after February 5, 2021 have had the option to participate in the extended follow-up. Only patients who provided consent for extended follow-up will be included in this study.

Patients who were enrolled to the vadadustat study prior to February 5, 2021 will be reconsented in order to participate. For these patients, an IRB approved letter was sent out to inform them of an opportunity to participate in the long-term follow-up. One week later, a study coordinator contacted these patients and provided additional information. For those patients that agreed to be part of the study, an electronic consent via RedCap was sent to the patient's email. Patients have the opportunity to review the informed consent form, provide personal information and sign.

19. Publication Plan

No publication or disclosure of study results will be permitted, except under the terms and conditions of a separate, written agreement between The University of Texas Health Science Center at Houston (the Sponsor) and the investigator and/or the investigator's institution. The Sponsor will have the opportunity to review and approve all proposed abstracts, manuscripts, or presentations regarding this study prior to submission for publication/presentation. Akebia will have the

opportunity to review all proposed abstracts, manuscripts, or presentations regarding this study prior to submission for publication/presentation. Any information identified by the Sponsor and Akebia as confidential will be deleted prior to submission.

For all publications relating to the study, the institution will comply with recognized ethical standards concerning publications and authorship, including: Section II - "Ethical Considerations in the Conduct and Reporting of Research" of the Uniform Requirements for Manuscripts Submitted to Biomedical Journals, http://www.icmje.org/index.html#authorship, established by the International Committee of Medical Journal Editors.

References

Abry J, Goodrich B. Prior Distributions for rstanarn Models, 2020. (https://cran.rproject.org/web/packages/rstanarm/vignettes/priors.html).

Appelhoff, R. J., Tian, Y. M., Raval, R. R., Turley, H., Harris, A. L., Pugh, C. W., Ratcliffe, P. J., and Gleadle, J. M. (2004). Differential function of the prolyl hydroxylases PHD1, PHD2, and PHD3 in the regulation of hypoxia-inducible factor. J Biol Chem 279, 38458-38465.

Assmann SF, Pocock SJ, Enos LE, Kasten LE. Subgroup analysis and other (mis)uses of baseline data in clinical trials. Lancet 2000;355:1064–9.

Berra, E., Roux, D., Richard, D. E., and Pouyssegur, J. (2001). Hypoxia-inducible factor-1α (HIF- 1α) escapes O2-driven proteasomal degradation irrespective of its subcellular localization: nucleus or cytoplasm. EMBO Rep 2, 615-620.

Berry DA. Bayesian clinical trials. Nature Reviews Drug Discovery 2006:5, 27-36.

Best N, Thomas, A. (2000) . (Editors). (pp. 387-402). Bayesian Graphical Models and Software for GLMs. In: D. Dey, S. K. Ghosh, Mallick BK, eds. Generalized Linear Models: A Bayesian Perspective. New York: Marcel Dekker, Inc., 2000.

Bishop, T., and Ratcliffe, P. J. (2014). Signaling hypoxia by hypoxia-inducible factor protein hydroxylases: a historical overview and future perspectives. Hypoxia (Auckl) 2, 197-213.

Bowser, J. L., Lee, J. W., Yuan, X., and Eltzschig, H. K. (2017). The Hypoxia-Adenosine Link during Inflammation. J Appl Physiol (1985), jap 00101 02017.

Cao, X. (2020). COVID-19: immunopathology and its implications for therapy. Nat Rev Immunol.

Carpenter B, Gelman A, Hoffman MD, et al. Stan, A Probabilistic Programming Language. J. Stat. Soft. 2017;76.

Clambey, E. T., McNamee, E. N., Westrich, J. A., Glover, L. E., Campbell, E. L., Jedlicka, P., de Zoeten, E. F., Cambier, J. C., Stenmark, K. R., Colgan, S. P., and Eltzschig, H. K. (2012). Hypoxia-inducible factor-1 alpha-dependent induction of FoxP3 drives regulatory T-cell abundance and function during inflammatory hypoxia of the mucosa. Proc Natl Acad Sci U S A 109, E2784-2793.

Clerici, C., and Planes, C. (2009). Gene regulation in the adaptive process to hypoxia in lung epithelial cells. Am J Physiol 296, L267.

Colgan, S. P., Furuta, G. T., and Taylor, C. T. (2020). Hypoxia and Innate Immunity: Keeping Up with the HIFsters. Annu Rev Immunol 38, 341-363.

Cummins, E. P., and Taylor, C. T. (2017). Hypoxia and inflammation. Biochemist 39, 34-36.

Eckardt, K.-U., Bernhardt, W., Willam, C., and Wiesener, M. (2007). Hypoxia-inducible transcription factors and their role in renal disease. Semin Nephrol 27, 363-372.

Eckle, T., Brodsky, K., Bonney, M., Packard, T., Han, J., Borchers, C. H., Mariani, T. J., Kominsky, D. J., Mittelbronn, M., and Eltzschig, H. K. (2013a). HIF1A reduces acute lung injury by optimizing carbohydrate metabolism in the alveolar epithelium. PLoS Biol 11, e1001665.

Eckle, T., Faigle, M., Grenz, A., Laucher, S., Thompson, L. F., and Eltzschig, H. K. (2008a) A2B adenosine receptor dampens hypoxia-induced vascular leak. Blood 111, 2024-2035.

Eckle, T., Fullbier, L., Wehrmann, M., Khoury, J., Mittelbronn, M., Ibla, J., Rosenberger, P., and Eltzschig, H. K. (2007). Identification of ectonucleotidases CD39 and CD73 in innate protection during acute lung injury. J Immunol 178, 8127-8137.

Eckle, T., Grenz, A., Laucher, S., and Eltzschig, H. K. (2008b). A2B adenosine receptor signaling attenuates acute lung injury by enhancing alveolar fluid clearance in mice. J Clin Invest 118, 3301-3315.

Eckle, T., Hartmann, K., Bonney, S., Reithel, S., Mittelbronn, M., Walker, L. A., Lowes, B. D., Han, J., Borchers, C. H., Buttrick, P. M., et al. (2012). Adora2b-elicited Per2 stabilization promotes a HIFdependent metabolic switch crucial for myocardial adaptation to ischemia. Nat Med 18, 774-782.

Eckle, T., Hughes, K., Ehrentraut, H., Brodsky, K. S., Rosenberger, P., Choi, D. S., Ravid, K., Weng, T., Xia, Y., Blackburn, M. R., and Eltzschig, H. K. (2013b). Crosstalk between the equilibrative nucleoside transporter ENT2 and alveolar Adora2b adenosine receptors dampens acute lung injury. FASEB J 27, 3078-3089.

Eckle, T., Kewley, E. M., Brodsky, K. S., Tak, E., Bonney, S., Gobel, M., Anderson, D., Glover, L. E., Riegel, A. K., Colgan, S. P., and Eltzschig, H. K. (2014). Identification of hypoxia-inducible factor HIF1A as transcriptional regulator of the A2B adenosine receptor during acute lung injury. J Immunol 192, 1249-1256.

Eckle, T., Kohler, D., Lehmann, R., El Kasmi, K., and Eltzschig, H. K. (2008c). Hypoxiainducible factor-1 is central to cardioprotection: a new paradigm for ischemic preconditioning. Circulation 118, 166-175.

Eckle, T., Kohler, D., Lehmann, R., El Kasmi, K. C., and Eltzschig, H. K. (2008d). HypoxiaInducible Factor-1 Is Central to Cardioprotection: A New Paradigm for Ischemic Preconditioning. Circulation 118, 166-175.

Eltzschig, H. K., Bratton, D. L., and Colgan, S. P. (2014). Targeting hypoxia signalling for the treatment of ischaemic and inflammatory diseases. Nat Rev Drug Discov 13, 852-869.

Eltzschig, H. K., and Carmeliet, P. (2011). Hypoxia and inflammation. N Engl J Med 364, 656665.

Eltzschig, H. K., Sitkovsky, M. V., and Robson, S. C. (2012). Purinergic signaling during inflammation. N Engl J Med 367, 2322-2333.

FDA. Innovation/Stagnation, Challenge and Opportunity on the Critical Path to New Medical Products, 2004.

FDA. Innovation or Stagnation, Critical Path Opportunities List, 2006.

Fitzmaurice, G.M. & Laird, N.M. Generalized linear mixture models for handling nonignorable dropouts in longitudinal studies. Biostatistics 2000:1(2): 141-156.

Fong, G. H., and Takeda, K. (2008). Role and regulation of prolyl hydroxylase domain proteins. Cell Death Differ 15, 635-641.

Fraisl, P., Aragones, J., and Carmeliet, P. (2009). Inhibition of oxygen sensors as a therapeutic strategy for ischaemic and inflammatory disease. Nat Rev Drug Discov 8, 139-152.

Fu, X., and Zhang, F. (2018). Role of the HIF-1 signaling pathway in chronic obstructive pulmonary disease. Exp Ther Med 16, 4553-4561.

Gelman A. Prior distributions for variance parameters in hierarchical models. Bayesian Analysis 2006:1(3), pp.515-533.

Gelman A, Hill J. Data analysis using regression and multilevel. Cambridge: Cambridge University Press, 2007.

Goodman SN. Introduction to Bayesian methods I, measuring the strength of the evidence. Clinical Trials and Meta-Analysis 2005;2:282–90.

Gong, H., Rehman, J., Tang, H., Wary, K., Mittal, M., Chaturvedi, P., Zhao, Y. Y., Komarova, Y. A., Vogel, S. M., and Malik, A. B. (2015). HIF2alpha signaling inhibits adherens junctional disruption in acute lung injury. J Clin Invest 125, 652-664.

Guo, Y., Feng, L., Zhou, Y., Sheng, J., Long, D., Li, S., and Li, Y. (2015). Systematic review with metaanalysis: HIF-1α attenuates liver ischemia-reperfusion injury. Transplant Rev (Orlando) 29, 127-134.

Haase, V. H. (2017). Oxygen sensors as therapeutic targets in kidney disease. Nephrol Ther 13 Suppl 1, S29-S34.

Hart, M. L., Grenz, A., Gorzolla, I. C., Schittenhelm, J., Dalton, J. H., and Eltzschig, H. K. (2011). Hypoxia-inducible factor-1alpha-dependent protection from intestinal ischemia/reperfusion injury involves ecto-5'-nucleotidase (CD73) and the A2B adenosine receptor. J Immunol 186, 4367-4374.

Heitrich, M., Garcia, D. M., Stoyanoff, T. R., Rodriguez, J. P., Todaro, J. S., and Aguirre, M. V. (2016). Erythropoietin attenuates renal and pulmonary injury in polymicrobial induced-sepsis through EPO-R, VEGF and VEGF-R2 modulation. Biomed Pharmacother 82, 606-613.

Heyman, S. N., Leibowitz, D., Mor-Yosef Levi, I., Liberman, A., Eisenkraft, A., Alcalai, R., Khamaisi, M., and Rosenberger, C. (2016). Adaptive response to hypoxia and remote ischaemia pre-conditioning: a new hypoxia-inducible factors era in clinical medicine. Acta Physiol 216, 395- 406.

Hill, P., Shukla, D., Tran, M. G., Aragones, J., Cook, H. T., Carmeliet, P., and Maxwell, P. H. (2008). Inhibition of hypoxia inducible factor hydroxylases protects against renal ischemiareperfusion injury. J Am Soc Nephrol 19, 39-46.

Hoegl, S., Brodsky, K. S., Blackburn, M. R., Karmouty-Quintana, H., Zwissler, B., and Eltzschig, H. K. (2015). Alveolar Epithelial A2B Adenosine Receptors in Pulmonary Protection during Acute Lung Injury. J Immunol 195, 1815-1824.

Huang, C., Wang, Y., Li, X., Ren, L., Zhao, J., Hu, Y., Zhang, L., Fan, G., Xu, J., Gu, X., et al. (2020). Clinical features of patients infected with 2019 novel coronavirus in Wuhan, China. Lancet 395, 497-506.

Huang, X., Zhang, X., Zhao, D. X., Yin, J., Hu, G., Evans, C. E., and Zhao, Y. Y. (2019). Endothelial Hypoxia-Inducible Factor-1alpha Is Required for Vascular Repair and Resolution of Inflammatory Lung Injury through Forkhead Box Protein M1. Am J Pathol 189, 1664-1679.

Idzko, M., Ferrari, D., and Eltzschig, H. K. (2014a). Nucleotide signalling during inflammation. Nature 509, 310-317.

Idzko, M., Ferrari, D., Riegel, A. K., and Eltzschig, H. K. (2014b). Extracellular nucleotide and nucleoside signaling in vascular and blood disease. Blood 124, 1029-1037.

Irony T. Bayesian Medical Device Clinical Trials in the Regulatory Setting. In:, 2008.

Ito, M., Tanaka, T., Ishii, T., Wakashima, T., Fukui, K., and Nangaku, M. (2020). Prolyl hydroxylase inhibition protects the kidneys from ischemia via upregulation of glycogen storage. Kidney Int 97, 687701.

Joharapurkar, A. A., Pandya, V. B., Patel, V. J., Desai, R. C., and Jain, M. R. (2018). Prolyl Hydroxylase Inhibitors: A Breakthrough in the Therapy of Anemia Associated with Chronic Diseases. J Med Chem 61, 6964-6982.

Kant, R., Bali, A., Singh, N., and Jaggi, A. S. (2013). Prolyl 4 hydroxylase: a critical target in the pathophysiology of diseases. Korean J Physiol Pharmacol 17, 111-120.

Kapitsinou, P. P., Sano, H., Michael, M., Kobayashi, H., Davidoff, O., Bian, A., Yao, B., Zhang, M. Z., Harris, R. C., Duffy, K. J., et al. (2014). Endothelial HIF-2 mediates protection and recovery from ischemic kidney injury. J Clin Invest 124, 2396-2409.

Ke, Q., and Costa, M. (2006). Hypoxia-inducible factor-1 (HIF-1). Mol Pharmacol 70, 14691480.

Kiers, D., Wielockx, B., Peters, E., van Eijk, L. T., Gerretsen, J., John, A., Janssen, E., Groeneveld, R., Peters, M., Damen, L., et al. (2018). Short-Term Hypoxia Dampens Inflammation in vivo via Enhanced Adenosine Release and Adenosine 2B Receptor Stimulation. EBioMedicine 33, 144-156.

Koivunen, P., and Kietzmann, T. (2018). Hypoxia-Inducible Factor Prolyl 4-Hydroxylases and Metabolism. Trends Mol Med 24, 1021-1035.

Lee, J. W., Ko, J., Ju, C., and Eltzschig, H. K. (2019). Hypoxia signaling in human diseases and therapeutic targets. Exp Mol Med 51, 1-13.

Lilford RJ, Thornton JG, Braunholtz D. Clinical trials and rare diseases: a way out of a conundrum. BMJ 1995;311:1621–5.

Li, M., Li, G., Yu, B., Luo, Y., and Li, Q. (2020a). Activation of Hypoxia-Inducible Factor-1alpha Via Succinate Dehydrogenase Pathway During Acute Lung Injury Induced by Trauma/Hemorrhagic Shock. Shock 53, 208-216.

Li, M., Li, G., Yu, B., Luo, Y., and Li, Q. (2020b). Activation of Hypoxia-Inducible Factor-1α Via Succinate Dehydrogenase Pathway During Acute Lung Injury Induced by Trauma/Hemorrhagic Shock. Shock 53, 208-216.

Luo, R., Zhang, W., Zhao, C., Zhang, Y., Wu, H., Jin, J., Zhang, W., Grenz, A., Eltzschig, H. K., Tao, L., et al. (2015). Elevated Endothelial Hypoxia-Inducible Factor-1alpha Contributes to Glomerular Injury and Promotes Hypertensive Chronic Kidney Disease. Hypertension 66, 75-84.

Masson, N., and Ratcliffe, P. J. (2003). HIF prolyl and asparaginyl hydroxylases in the biological response to intracellular O(2) levels. J Cell Sci 116, 3041-3049.

McClendon, J., Jansing, N. L., Redente, E. F., Gandjeva, A., Ito, Y., Colgan, S. P., Ahmad, A., Riches, D. W. H., Chapman, H. A., Mason, R. J., et al. (2017). Hypoxia-Inducible Factor 1alpha Signaling Promotes Repair of the Alveolar Epithelium after Acute Lung Injury. Am J Pathol 187, 1772-1786.

Mirakaj, V., Thix, C. A., Laucher, S., Mielke, C., Morote-Garcia, J. C., Schmit, M. A., Henes, J., Unertl, K. E., Kohler, D., and Rosenberger, P. (2010). Netrin-1 dampens pulmonary inflammation during acute lung injury. Am J Respir Crit Care Med 181, 815-824.

Morote-Garcia, J. C., Rosenberger, P., Nivillac, N. M., Coe, I. R., and Eltzschig, H. K. (2009). Hypoxiainducible factor-dependent repression of equilibrative nucleoside transporter 2 attenuates mucosal inflammation during intestinal hypoxia. Gastroenterology 136, 607-618.

Mutz, C., Mirakaj, V., Vagts, D. A., Westermann, P., Waibler, K., Konig, K., Iber, T., NoldgeSchomburg, G., and Rosenberger, P. (2010). The neuronal guidance protein netrin-1 reduces alveolar inflammation in a porcine model of acute lung injury. Crit Care 14, R189.

Myllyharju, J. (2013). Prolyl 4-hydroxylases, master regulators of the hypoxia response. Acta Physiol (Oxf) 208, 148-165.

Nagamine, Y., Tojo, K., Yazawa, T., Takaki, S., Baba, Y., Goto, T., and Kurahashi, K. (2016). Inhibition of Prolyl Hydroxylase Attenuates Fas Ligand-Induced Apoptosis and Lung Injury in Mice. Am J Respir Cell Mol Biol 55, 878-888.

Natarajan, R., Salloum, F. N., Fisher, B. J., Kukreja, R. C., and Fowler, A. A., 3rd (2006). Hypoxia inducible factor-1 activation by prolyl 4-hydroxylase-2 gene silencing attenuates myocardial ischemia reperfusion injury. Circ Res 98, 133-140.

O'Neill RT. FDA's Critical Path Initiative, A Perspective on the Contribution of Biostatistics. Biometrical Journal. 2006:48(4): 559-564.

Peyssonnaux, C., Zinkernagel, A. S., Schuepbach, R. A., Rankin, E., Vaulont, S., Haase, V. H., Nizet, V., and Johnson, R. S. (2007). Regulation of iron homeostasis by the hypoxia-inducible transcription factors (HIFs). J Clin Invest 117, 1926-1932.

Pocock SJ, Assmann SF, Enos LE, Kasten LE. Subgroup analysis, covariate adjustment and baseline comparisions in clinical trial reporting, Current practice and problems. Stat Med 2002;21:2917–30.

Prabhakar, N. R., and Semenza, G. L. (2015). Oxygen sensing and homeostasis. Physiology 30, 340-348.

Pugh, C. W., and Ratcliffe, P. J. (2003). Regulation of angiogenesis by hypoxia: role of the HIF system. Nat Med (N Y, NY, U S) 9, 677-684.

R Core Team. R: A Language and Environment for Statistical Computing. Vienna, Austria: R Foundation for Statistical Computing, 2017.

Rabinowitz, M. H. (2013). Inhibition of hypoxia-inducible factor prolyl hydroxylase domain oxygen sensors: tricking the body into mounting orchestrated survival and repair responses. J Med Chem 56, 9369-9402.

Rajendran, G., Schonfeld, M. P., Tiwari, R., Huang, S., Torosyan, R., Fields, T., Park, J., Susztak, K., and Kapitsinou, P. P. (2020). Inhibition of Endothelial PHD2 Suppresses PostIschemic Kidney Inflammation through Hypoxia-Inducible Factor-1. J Am Soc Nephrol 31, 501516.

Ranieri, V. M., Rubenfeld, G. D., Thompson, B. T., Ferguson, N. D., Caldwell, E., Fan, E., Camporota, L., and Slutsky, A. S. (2012). Acute respiratory distress syndrome: the Berlin Definition. JAMA 307, 2526-2533.

Reutershan, J., Vollmer, I., Stark, S., Wagner, R., Ngamsri, K. C., and Eltzschig, H. K. (2009). Adenosine and inflammation: CD39 and CD73 are critical mediators in LPS-induced PMN trafficking into the lungs. FASEB J 23, 473-482.

Robinson, A., Keely, S., Karhausen, J., Gerich, M. E., Furuta, G. T., and Colgan, S. P. (2008). Mucosal protection by hypoxia-inducible factor prolyl hydroxylase inhibition. Gastroenterology 134, 145-155.

Rocha, J., Eduardo-Figueira, M., Barateiro, A., Fernandes, A., Brites, D., Pinto, R., Freitas, M., Fernandes, E., Mota-Filipe, H., and Sepodes, B. (2015). Erythropoietin reduces acute lung injury and multiple organ failure/dysfunction associated to a scald-burn inflammatory injury in the rat. Inflammation 38, 312-326.

Rosenberger, P., Schwab, J. M., Mirakaj, V., Masekowsky, E., Mager, A., Morote-Garcia, J. C., Unertl, K., and Eltzschig, H. K. (2009). Hypoxia-inducible factor-dependent induction of netrin-1 dampens inflammation caused by hypoxia. Nat Immunol 10, 195-202. SAS Institute I. SAS v. 9.4. Cary, NC USA: SAS Institute, Inc, 2002-2012.

Schneider, M., Van Geyte, K., Fraisl, P., Kiss, J., Aragones, J., Mazzone, M., Mairbaeurl, H., De Bock, K., Jeoung, N. H., Mollenhauer, M., et al. (2010). Loss or silencing of the PHD1 prolyl hydroxylase protects livers of mice against ischemia/reperfusion injury. Gastroenterology 138, 1143-1154.

Schodel, J., and Ratcliffe, P. J. (2019). Mechanisms of hypoxia signalling: new implications for nephrology. Nat Rev Nephrol 15, 641-659.

Semenza, G. L. (1999). Expression of Hypoxia-inducible Factor 1: Mechanisms and Consequences. 59, 47-53.

Semenza, G. L. (2011). Oxygen sensing, homeostasis, and disease. The New England journal of medicine 365, 537-547.

Semenza, G. L. (2012). Hypoxia-inducible factors in physiology and medicine. Cell 148, 399408.

Shimoda, L. A., and Semenza, G. L. (2011). HIF and the lung: role of hypoxia-inducible factors in pulmonary development and disease. Am J Respir Crit Care Med 183, 152-156.

Siddiq, A., Aminova, L. R., and Ratan, R. R. (2007). Hypoxia inducible factor prolyl 4hydroxylase enzymes: center stage in the battle against hypoxia, metabolic compromise and oxidative stress. Neurochem Res 32, 931-946.

Sridharan, V., Guichard, J., Bailey, R. M., Kasiganesan, H., Beeson, C., and Wright, G. L. (2007). The prolyl hydroxylase oxygen-sensing pathway is cytoprotective and allows maintenance of mitochondrial membrane potential during metabolic inhibition. Am J Physiol Cell Physiol 292, C719-728.

Spiegelhalter DJ, Abrams KR, Myles JP. Bayesian approaches to clinical trials and health-care evaluation. Chichester: John Wiley & Sons, 2004

Spiegelhalter DJ, Myles JP, Jones, Abrams K. An introduction to Bayesian methods in health technology assessment. BMJ 1999;319:508–12.

Tanaka, S., Tanaka, T., and Nangaku, M. (2015). Hypoxia and Dysregulated Angiogenesis in Kidney Disease. Kidney Dis (Basel) 1, 80-89.

Thompson, B. T., Chambers, R. C., and Liu, K. D. (2017). Acute Respiratory Distress Syndrome. N Engl J Med 377, 562-572.

Temple R. How the FDA currently makes decisions on clinical studies. Clinical Trials and MetaAnalysis;2:276–81.

Urrutia, A. A., and Aragones, J. (2018). HIF Oxygen Sensing Pathways in Lung Biology. Biomedicines 6.

Vohwinkel, C. U., Hoegl, S., and Eltzschig, H. K. (2015). Hypoxia signaling during acute lung injury. J Appl Physiol (1985) 119, 1157-1163.

Ware, L. B., and Matthay, M. A. (2000). The Acute Respiratory Distress Syndrome. N Engl J Med 342, 1334-1349.

Wijeysundera DN, Austin PC, Hux JE, Beattie WS, Laupacis A. Bayesian statistical inference enhances the interpretation of contemporary randomized controlled trials. Journal of Clinical Epidemiology 2009;62:13-21 e5.

Woodcock J. FDA introductory comments, clinical studies design and evaluation issues. Clinical Trials and Meta-Analysis;2:273–5.

Wu, C., Chen, X., Cai, Y., Xia, J., Zhou, X., Xu, S., Huang, H., Zhang, L., Zhou, X., Du, C., et al. (2020). Risk Factors Associated With Acute Respiratory Distress Syndrome and Death in Patients With Coronavirus Disease 2019 Pneumonia in Wuhan, China. JAMA Intern Med.

Xu, Z., Shi, L., Wang, Y., Zhang, J., Huang, L., Zhang, C., Liu, S., Zhao, P., Liu, H., Zhu, L., et al. (2020). Pathological findings of COVID-19 associated with acute respiratory distress syndrome. Lancet Respir Med 8, 420-422.

Yang, Y., Yu, X., Zhang, Y., Ding, G., Zhu, C., Huang, S., Jia, Z., and Zhang, A. (2018). Hypoxia-inducible factor prolyl hydroxylase inhibitor roxadustat (FG-4592) protects against cisplatin- induced acute kidney injury. Clin Sci 132, 825-838.

Zhou, F., Yu, T., Du, R., Fan, G., Liu, Y., Liu, Z., Xiang, J., Wang, Y., Song, B., Gu, X., et al. (2020). Clinical course and risk factors for mortality of adult inpatients with COVID-19 in Wuhan, China: a retrospective cohort study. Lancet 395, 1054-1062.

Zhu, M., Wang, L., Yang, J., Xie, K., Zhu, M., Liu, S., Xu, C., Wang, J., Gu, L., Ni, Z., et al. (2019). Erythropoietin Ameliorates Lung Injury by Accelerating Pulmonary Endothelium Cell Proliferation via Janus Kinase-Signal Transducer and Activator of Transcription 3 Pathway After Kidney Ischemia and Reperfusion Injury. Transplant Proc 51, 972-978.

VSTAT COVID: Statistical Analysis Plan

CONTENTS

Synopsis 2
GENERAL STATISTICAL CONSIDERATIONS 3
Analysis Sample 3
Patient Accountability & Compliance 3
Randomization 3
Treatment Group Comparability 4
Preliminary Analysis 4
Multiplicity 4
Missing Data 4
EFFICACY ANALYSIS 4
General Data Analytic Strategy 4
Primary Outcome 5
Primary Analysis 5
Data Analytic Models 5
Sensitivity Analysis 7
Subgroup Analyses by Gender, Race, Ethnicity 7
SAMPLE SIZE JUSTIFICATION 7
References 8

SYNOPSIS

VSTAT COVID is a phase 2 multicenter, randomized double-blind placebo-controlled Bayesian clinical trial to estimate efficacy of vadadustat for the prevention and treatment of acute respiratory distress syndrome (ARDS) in hospitalized patients with Coronavirus disease 2019 (COVID-19). Developing effective interventions for ARDS requires incremental improvement of theoretically sound treatments based on systematically accruing data. Often such incremental development is hampered by statistical tools not appropriate to the task. Classical, Frequentist statistics have advanced the field, but are less informative for the initial evaluation of a new treatment. The reliance of the Frequentist framework on dichotomous, null hypothesis-testing provides some control of the error rate in the context of multiple repeated trials; however, this is not what early-phase treatment testing requires. Developing nascent treatments requires investigators to bet on an alternative hypothesis. Investigators evaluating a theoretically sound intervention want to know the probability that the approach confers some level of benefit given the observed data: that is, they want to know the probability that

the alternative hypothesis is true. While Frequentist inference does not directly address this issue, Bayesian statistical inference provides a principled approach to answer this question. Indeed, addressing the so-called "Pipeline Problem" in developing clinical applications, the FDA has indicated that Bayesian statistics offers one avenue for improved methodological efficiency [1–7].

Decision-making, based on an initial treatment trial, is assisted by estimates of the probability of an effect of some specified magnitude. These statements, not part of the conventional, Frequentist statistical lexicon, are accessible via Bayesian approaches, particularly with small sample sizes [8,9]. Detailed descriptions of Bayesian statistical reasoning exist elsewhere [10–12]. Succinctly, Frequentist models estimate the probability of observing the data (or data more extreme) given that the null hypothesis is true; Bayesian analyses estimate the probability of the alternative hypothesis given the observed data [12]. Bayesian probability estimates incorporate prior information about plausible parameter values (i.e., the prior distribution) and the observed data (i.e., the likelihood). Combining these two distributions forms the posterior distribution which permits evaluation of the probability that the true value of the parameter falls in some range.

GENERAL STATISTICAL CONSIDERATIONS

Analysis Sample

The primary analysis sample for efficacy will implement intention-to-treat (ITT) principles. The safety evaluation will include all subjects while they were taking study treatment.

The design will proceed as in Figure 1 below. Randomization to condition (a maximum of n = 325 per condition; N = 650) will provide efficacy estimates.

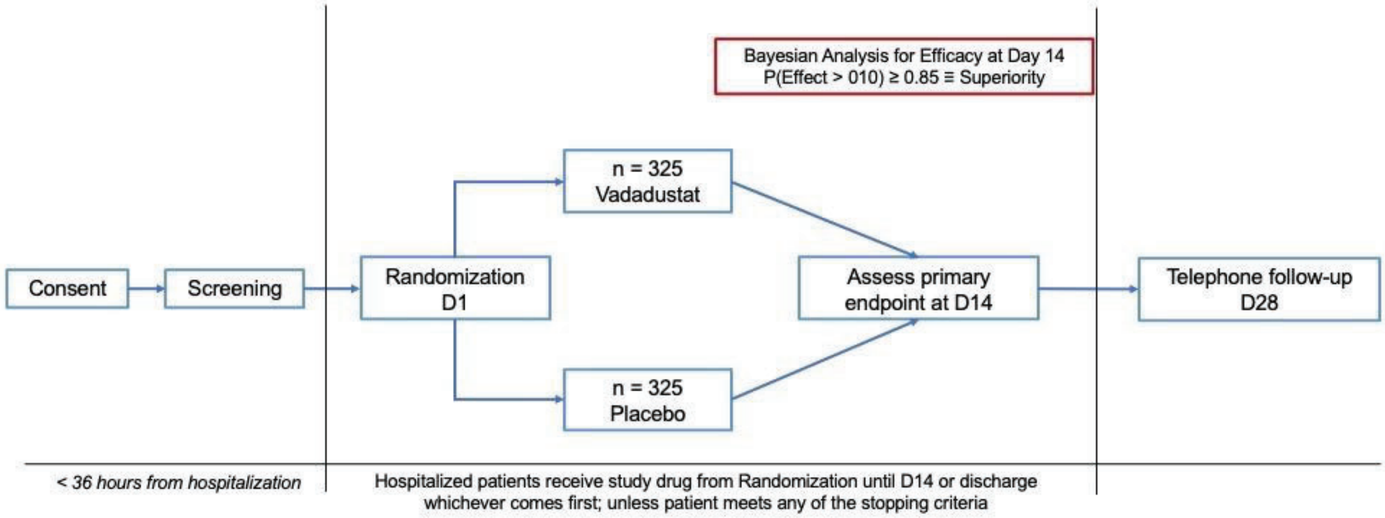

Patient Accountability & Compliance

A flowchart (CONSORT Diagram) will summarize participant status, listing the number and disposition of patients randomized to each treatment: (1) Placebo and (2) vadadustat 900 mg. Specifically, within each group, the CONSORT Diagram will list the numbers of patients who completed the study, withdrew consent, death, and those lost to follow up.

Randomization

Randomization (1:1 ratio) is stratified by severity (mSOFA < 4) and site with random blocks of 2 or 4.

Treatment Group Comparability

Initial analyses examining group differences for baseline variables will use cross-tabulation, ANOVAs, and examination of correlations between baseline variables and specified outcomes. For the purposes of evaluating the comparability of groups, a posterior probability of > 95% will constitute evidence for statistically reliable differences. Baseline or demographic variables on which group differences are detected, and which are correlated with outcomes, meet the definition of confounders[13,14] and will result in two sets of analyses: one in which the relevant variable is included as a covariate and one in which it is not. This will permit determination of the degree to which any group differences might confound conclusions regarding treatment.

Preliminary Analysis

For all continuous variables, outliers will be explored and extreme outliers will be queried to confirm that they are not erroneous before the data is locked for analysis, but outliers will not be removed from the analysis.

Multiplicity

In keeping with sound Bayesian analytic principles, salient error rates/operating characteristics for confirmatory analyses in each component of the trial, provided in the sample size justification section, result from Monte Carlo simulation. Any secondary analyses for which issues of multiplicity might be a consideration will use weakly, informative priors to regularize all estimates. Means for these regularizing priors will be centered on the null hypothesis, with variances determined by the scale of the data and credible effects previously reported in the literature in the most closely analogous studies. Of note is the principle that the more informative the priors are, based on credible estimates of these effects, the greater the degree of regularization, the more conservative the estimates, and the more likely the results are to replicate outside of the current sample. Indeed for any observed results, the Bayesian approach makes it possible to determine the sensitivity of the results to prior assumptions; the degree of prior skepticism an observer would require before dismissing the estimated treatment effect.[15] In the interest of transparency and reproducibility, the resulting reports will provide the prior specifications used for these secondary analyses.

Missing Data

Under the Intent-To-Treat (ITT) principle, all patients who are randomized are included in the analysis. Therefore, missing data, especially in the primary outcome measure, can be problematic. Missing data will result in joint modeling of observed outcomes and the missing data which is robust to ignorable missingness[16]. Sensitivity analyses will evaluate robustness of analytic conclusions to missing data. Non-ignorable missing data patterns will be addressed through pattern-mixture modeling methods[17].

EFFICACY ANALYSIS

General Data Analytic Strategy

VSTAT COVID focuses on estimating the effect of vadadustat 900 mg relative to placebo among adult hospital admissions with lab-confirmed COVID-19 diagnoses at treatment day 14, on probability of death (8) or hospitalization, on invasive mechanical ventilation or ECMO (7), or hospitalization, on non-invasive ventilation or high-flow oxygen devices (6) on the NIAID-OS. Our primary outcome is the probability of demonstrating this status at day 14 of treatment, and our primary analytical approach will involve Bayesian statistical methods to assess the probability that such an effect exists. Our analytical plan is shaped by the limitations of conventional (Frequentist) methods for addressing this question and the advantages of a Bayesian approach for assessing the probability that a given strategy might successfully be expanded into a larger-scale program for the prevention and treatment of ARDS. This data, valuable in its own right, can justify the commitment of resources

needed for such an expansion. Further, current uncertainty regarding the probability of outcome status on the NIAID-OS (i.e. categories 6-8) as a function of vadadustat is readily incorporated in a Bayesian approach permitting more robust trial planning and design.

The Bayesian approach addresses these questions: “Among patients with lab-confirmed COVID-19, what is the probability that vadadustat confers benefit relative to placebo on status on the NIAID-OS at treatment day 14? What is the best estimate of this effect? What is its precision?” By estimating the probability that such an effect exists, we are assessing the probability that the alternative hypothesis is true, a probability that is, by definition, not accessible to Frequentist methods. The FDA has discussed the use of Bayesian statistical methods to make decisions regarding the efficacy of new treatments as an alternative to Frequentist methods in developing clinical applications.[1,2,4,5,11,18] The current proposal will provide the optimal, unbiased estimates for the benefit conferred by vadadustat, while also estimating the probability that such effects exist. Posterior distributions can then be used as informative priors for continued monitoring in expansions of treatments and treatment strategies exhibiting initial promise.

Primary Outcome

The efficacy contrast will evaluate the proportion of patients at treatment day 14 who are dead (8) or hospitalized, on invasive mechanical ventilation or ECMO (7) or hospitalized, on non-invasive ventilation or high flow oxygen devices (6) on the NIAID-OS.

Primary Analysis

Initial analyses examining group differences for baseline variables will use cross-tabulation, ANOVAs, and examination of correlations between baseline variables and specified outcomes. For the purposes of evaluating the comparability of groups, a posterior probability of > 95% will constitute evidence for statistically reliable differences. Baseline or demographic variables on which group differences are detected, and which are correlated with outcomes, meet the definition of confounders[13,14] and will result in two sets of analyses: one in which the relevant variable is included as a covariate and one in which it is not. This will permit determination of the degree to which any group differences might confound conclusions regarding treatment.

Broadly, the data analytic strategy will use generalized linear and multilevel models (R and Stan) for both discrete, and continuous outcomes.[19–22] Multilevel generalized linear modeling to account for clustering of patients within site and repeated observations within patients will evaluate continuous, dichotomous, and count data. All analyses will apply intention-to-treat (ITT) principles with missingness addressed via joint modeling of observed outcomes and the missing data, an approach robust to ignorable missingness (i.e., MCAR and MAR).[16] Sensitivity analyses will evaluate robustness of analytic conclusions to missing data. Non-ignorable missing data patterns will be addressed through pattern-mixture modeling methods (Fitzmaurice & Laird 2000).[17]

Evaluation of posterior distributions will permit statements regarding the probability that effects of varying magnitudes exist, given the data. Specification of diffuse, neutral priors will reflect the initial uncertainty regarding effect sizes. For all generalized linear models, priors for (non-intercept) regression coefficients will be specified as ~Normal (μ=0, σ^2=1 x 10), level one and two error variances will be specified as ~Half- Normal (μ=0, σ^2=1 x 10^3). The choice of prior distribution for level two variances will follow Gelman’s recommendations.[23] Priors for the comparison of proportions will be specified as ~Beta (α=1.0, β=1.0).

Data Analytic Models

Log-binomial regression will utilize the following model for evaluating probability of outcome:

$$log\left(Prob_{y_i=1}\right) = \beta_0 + \beta_1 * mSOFA + \beta_2 * Site_1 + \beta_3 * Site_2 + \beta_4 * Site_3 + \beta_5 * Site_4 + \beta_6 * Treatment_1$$

$$y \sim Bernoulli(Prob_{y_i=1})$$

Where dummy coding provides indicators for each constituent predictors unless the number of sites increases to a point that a random effect is more appropriate. Where β_6 represents the treatment effect for each Bernoulli distributed outcome: 1) NIAID-OS $\geq$ 6 (Days 7 and 14), 2) mSOFA > 0 (Days 7 and 14), 3) Ventilator-free survival as assessed by best NIAID-OS score (Days 7 and 14), 4) Overall survival (Days 7, 14 and 28), 5) Proportion of participants with hypotension (MAP<70 mm Hg or vasopressors to maintain blood pressure; Days 7 and 14), and 5) Proportion of participants with acute kidney injury (Days 7 and 14).

Generalized linear modeling will evaluate continuous outcomes:

$$y = e^{\beta_0 + \beta_1 * mSOFA + \beta_2 * Site_1 + \beta_3 * Site_2 + \beta_4 * Site_3 + \beta_5 * Site_4 + \beta_6 * Treatment_1}$$

Where *y* (i.e. mSOFA scores (Days 7 and 14) and NIAID-OS scores (Days 7 and 14) may follow a variety of response distributions including: 1) Normal, 2) T-distribution, 3) Gamma, 4) Log-Normal, 5) Hurdle-Gamma, 6) Hurdle-Log-Normal, 7) Cumulative Logit, and 8) Adjacent-Category models. Finally, analysis of time to event outcomes will use Cox proportional Hazards are parametric survival models.

Hypothesis Testing for Primary Outcome: *Among adult hospital admissions with lab-confirmed diagnosis of COVID-19, vadadustat 900 mg will demonstrate superiority to placebo as defined by a lower probability, at treatment day 14, of death (8) or hospitalization, on invasive mechanical ventilation or ECMO (7) or hospitalization, on non-invasive ventilation or high flow oxygen devices (6) on the NIAID-OS.* Logistic regression will evaluate the probability of patients demonstrating this status at day 14 of treatment as a function of treatment condition after adjusting for stratification variables. The salient contrast is:

$$Vstat - Plc = (\beta_0 + \beta_1 + \beta_2 + \beta_3 + \beta_4 + \beta_5 + \beta_6) - \left((\beta_0 + \beta_1 + \beta_2 + \beta_3 + \beta_4 + \beta_5)\right)$$

Hypothesis Testing for Secondary Outcome: *Among adult hospital admissions with lab-confirmed diagnosis of COVID-19, vadadustat 900 mg will demonstrate superiority to placebo as defined by a higher probability, at treatment day 14, of recovery on the MSOFA scale (MSOFA = 0)*. Generalized linear modeling will evaluate the probability of patients demonstrating an MSOFA = 0 at day fourteen of treatment as a function of treatment condition after adjusting for stratification variables. The salient contrast is:

$$Vstat - Plc = (\beta_0 + \beta_1 + \beta_2 + \beta_3 + \beta_4 + \beta_5 + \beta_6) - \left((\beta_0 + \beta_1 + \beta_2 + \beta_3 + \beta_4 + \beta_5)\right)$$

Exploratory Outcomes: Efficacy of vadadustat to induce known transcriptional targets of HIFs (e.g., erythropoietin, extracellular adenosine levels, circulating levels of CD73, VEGF, hypoxia-driven microRNAs that dampen lung inflammation) will be explored.

Various laboratory measurements (e.g., serum creatinine, FGF-23, markers of attenuated inflammation) will be measured and summarized.

Bayesian generalized multilevel models will evaluate the differential trajectories of HIF target-gene expression and inflammatory indicators as a function of treatment, time and the interaction of treatment and time. Additional analyses will evaluate HIF target-gene expression cross-sectionally at each time point as a function of treatment. Though theses analyses are, exploratory in nature, and Type I Error can be less of an issue in the in the Bayesian context we will evaluate the robustness of any identified effects by utilizing priors specified for the purpose of regularizing estimates. Regularization will render any identified effects more likely to demonstrate replication in future samples. Both double-exponential and the horseshoe distributed priors will provide regularization following hyper-prior distributions: Half-Student's t and Chi-square distributions respectively.[24–26] Specifying the degrees of freedom for each of these hyper-priors constrains the degree of

shrinkage the regularization approach can impose on coefficients. A priori we set this value at degrees of freedom = 1 for each hyper-prior. Priors essentially form a set of assumptions that require evaluation.[27] As such we will evaluate a family of hyper-priors on the shrinkage parameters identified by the specified degrees of freedom for both the double-exponential and horseshoe priors.[28] An initial random split of the data into a training set (70%) and test set (30%) will provide a basis for model building and model evaluation. Within the training set, analyses will examine both double-exponential and horseshoe priors for hyper-parameters ranging from one to at least ten degrees of freedom using k-fold cross-validation to determine the best model based as defined by the ELPD (or associated quantities including the KFOLD-IC, LOOCV-IC and WAIC).[29] The R package "brms" contains functions for specifying each type of hyper-prior, and in combination with the R package "loo" provide facilities for k-fold cross-validation.[29–31] Regarding all other model parameters (i.e. those not subject to regularization) intercepts will use ~Normal (μ = 0, σ^2 = 1 x 10^3) priors, level-one error variances will use ~Inverse Gamma (shape = 2.001, scale = 0.0001). Level-two variances will follow recommendations by Gelman (i.e. ~Half-T (df = 3, mean = 0, standard deviation = 10).[23]

Safety Monitoring

Safety monitoring will occur via the DSMB in accordance with FDA AE and SAE reporting requirements with interim safety evaluations occurring following 14 day follow-up of n = 30 and n = 120.

Sensitivity Analysis

Per protocol analyses will evaluate only those participants treated according to the protocol using the same methods described above and applied to the ITT analysis. Inconsistencies with the primary ITT analysis will result in cautious interpretation of the trial findings.

Subgroup Analyses by Gender, Race, Ethnicity

Secondary analyses will evaluate heterogeneity of treatment effect as a function of gender, age, ethnicity and NIAID-OS strata. The approach to subgroup analyses will utilize skeptical, informative priors to increase the likelihood of future replication.[32–34]

SAMPLE SIZE JUSTIFICATION

The trial will investigate the superiority of vadadustat 900 mg per day to placebo for the prevention and treatment of ARDS among COVID-19 patients, defined by the proportion of patients at day 14 who are dead (8) or hospitalized, on invasive mechanical ventilation or ECMO (7), or hospitalized, on noninvasive ventilation or high flow oxygen devices (6) on the NIAID-OS (i.e. decrease in the proportion of participants with NIAID-OS ≥ 6 at day 14).

Anticipated sample size for the current study is N = 650. An evaluation by the DSMB will occur following ascertainment of 14-day safety outcomes for the first 30 patients that are randomized and dosed, and a second at the first 120 (i.e. n = 60 receiving Vadadustat).

Bayesian analysis for superiority will occur following ascertainment of outcome at fourteen days after randomization of N = 650 patients. We stipulate that a posterior probability of a ≥ 2.5% decrease in the proportion of NIAID-OS ≥ 6 as a function of treatment that is ≥ 0.85 constitutes sufficient evidence to declare that the active treatment condition is superior to placebo. The prior distribution for analysis will be ~Beta(α= 1, β = 1), or in the presence of covarying for stratification factors ~Normal (μ=0, σ^2=1 x 10) for the coefficients in the log-form of the appropriate generalized linear model.

We anticipate a decrease in the proportion of NIAID-OS ≥ 6 participants due to treatment will be approximately 15%, with preliminary data suggesting a rate of 25.1% in the placebo condition. Assuming this

effect, K = 1000 Monte Carlo clinical trial simulations, for N = 650 participants and the previously stated decision rules resulted in detection of a > 2.5% decrease in NIAID-OS > 6 due to the active treatment 99.1% of the time with a Type I Error rate of 3.5%. For more modest effects, 12.5% and 10% decreases in NIAID-OS > 6, the current sample and decision rules will lead to detection 96.1% and 86.3% of the time with Type I Error rates of 4.8% and 3.7% respectively.

REFERENCES

1. FDA. Innovation/Stagnation, Challenge and Opportunity on the Critical Path to New Medical Products, 2004.

2. FDA. Innovation or Stagnation, Critical Path Opportunities List, 2006.

3. Irony T. Bayesian Medical Device Clinical Trials in the Regulatory Setting. In: , 2008.

4. O'Neill RT. FDA's Critical Path Initiative, A Perspective on the Contribution of Biostatistics. Biometrical Journal. 2006:48(4): 559-564.

5. Temple R. How the FDA currently makes decisions on clinical studies. Clinical Trials and Meta-Analysis;2:276–81.

6. Woodcock J. FDA introductory comments, clinical studies design and evaluation issues. Clinical Trials and Meta-Analysis;2:273–5.

7. Fitzmaurice, G.M. & Laird, N.M. Generalized linear mixture models for handling nonignorable dropouts in longitudinal studies. Biostatistics 2000:1(2): 141-156.

8. Lilford RJ, Thornton JG, Braunholtz D. Clinical trials and rare diseases: a way out of a conundrum. BMJ 1995;311:1621–5.

9. Spiegelhalter DJ, Myles JP, Jones, Abrams K. An introduction to Bayesian methods in health technology assessment. BMJ 1999;319:508–12.

10. Berry DA. Bayesian clinical trials. Nature Reviews Drug Discovery 2006:5, 27-36.

11. Goodman SN. Introduction to Bayesian methods I, measuring the strength of the evidence. Clinical Trials and Meta-Analysis 2005;2:282–90.

12. Wijeysundera DN, Austin PC, Hux JE, Beattie WS, Laupacis A. Bayesian statistical inference enhances the interpretation of contemporary randomized controlled trials. Journal of Clinical Epidemiology 2009;62:13-21 e5.

13. Assmann, S.F., Pocock, S.J., Enos, L.E. & Kasten, L.E. Subgroup analysis and other (mis)uses of baseline data in clinical trials. The Lancet 2000:355: 1064-1069.

14. Pocock SJ, Assmann SE, Enos LE, Kasten LE. Subgroup analysis, covariate adjustment and baseline comparisons in clinical trial reporting: current practice and problems. Stat Med 2002;21:2917–30.

15. Parmar MK, Ungerleider RS, Simon R. Assessing whether to perform a confirmatory randomized clinical trial. J Natl Cancer Inst 1996;88:1645–51.

16. Best N, Thomas, A. (2000) . (Editors). (pp. 387-402). Bayesian Graphical Models and Software for GLMs. In: D. Dey, S. K. Ghosh, Mallick BK, eds. Generalized Linear Models: A Bayesian Perspective. New York: Marcel Dekker, Inc., 2000.

17. Fitzmaurice, G.M. & Laird, N.M. Generalized linear mixture models for handling nonignorable dropouts in longitudinal studies. Biostatistics 2000:1(2): 141-156.

18. Berry DA. Introduction to Bayesian methods III: use and interpretation of Bayesian tools in design and analysis. Clin Trials 2005;2:295-300; discussion 301-4, 364-78.

19. Carpenter B, Gelman A, Hoffman MD, et al. Stan, A Probabilistic Programming Language. J. Stat. Soft. 2017;76.

20. Stan Development Team. The Stan Core Library. Stan Development Team, 2017.

21. R Core Team. R: A Language and Environment for Statistical Computing. Vienna, Austria: R Foundation for Statistical Computing, 2017.

22. R. The R Project http, //www.r-project.org.

23. Gelman A. Prior distributions for variance parameters in hierarchical models. Bayesian Analysis 2006:1(3), pp.515-533.
24. Piironen J, Vehtari A. Sparsity information and regularization in the horseshoe and other shrinkage priors, 2017. (http://arxiv.org/pdf/1707.01694).
25. Carvalho CM, Polson NG, Scott JG. The horseshoe estimator for sparse signals. Biometrika 2010;97:465–80.
26. Park, T & Casella, G. The Bayesian LASSO. Journal of the American Statistical Association 2008;103.
27. Gelman A, Hill J. Data analysis using regression and multilevel. Cambridge: Cambridge University Press, 2007.
28. Spiegelhalter DJ, Abrams KR, Myles JP. Bayesian approaches to clinical trials and health-care evaluation. Chichester: John Wiley & Sons, 2004.
29. Vehtari A, Gelman A, Gabry J. Practical Bayesian model evaluation using leave-one-out cross-validation and WAIC. Stat Comput 2017;27:1413–32.
30. Bürkner P-C. Bayesian Regression Models using Stan [R package brms version 1.10.0], 2017.
31. Bürkner P-C. brms, An R Package for Bayesian Multilevel Models Using Stan. J. Stat. Soft. 2017;80.
32. Cinciripini PM, Green CE, Robinson JD, et al. Benefits of varenicline vs. bupropion for smoking cessation: a Bayesian analysis of the interaction of reward sensitivity and treatment. Psychopharmacology 2017;234:1769–79.
33. Dixon DO, Simon R. Bayesian subset analysis. Biometrics 1991;47:871–81.
34. Dixon DO, Simon R. Bayesian subset analysis in a colorectal cancer clinical trial. Stat Med 1992;11:13–22.

VSTAT COVID: Sensitivity Analysis for Missing Outcome Data

Analyses used all randomized participants without imputing outcomes (Unimputed). Subsequent analyses imputed all missing data in the Control condition as a positive outcome and all missing data in the VSTAT condition as a negative outcome (Analysis 1). Conversely, Analysis 2 imputed all missing data in the Control condition as a negative outcome and all missing data in the VSTAT condition as a positive outcome. This brackets the Unimputed estimates in the two most extreme cases for each of these outcome variables. The primary outcome NIAID≥6 at Day 14 did not differ substantially across imputation strategies. With few exceptions 95% Credible Intervals for each approach overlapped. Cases in which this did not occur were those with extremely infrequent outcomes (Death at Days 7, 14, and 28) which can result in highly variable relative risks in the context of low event counts, and for Hypotension at Days 7 and 14 which is likely due to the high proportions of missingness introducing high variability into potential estimates.

Sensitivity Analysis for Missing Data with Dichotomous Outcomes (Log-Binomial Model)

Outcome	Unimputed						Analysis 1						Analysis 2					
	N	RR	L95	U95	PP(RR>1)	PP(RR<1)	N	RR	L95	U95	PP(RR>1)	PP(RR<1)	N	RR	L95	U95	PP(RR>1)	PP(RR<1)
NIAID ≥6 Day 7	440	0.86	0.72	1.00	0.03	0.97	448	0.88	0.74	1.03	0.05	0.95	448	0.85	0.71	0.99	0.02	0.98
NIAID ≥6 Day 14	439	0.79	0.58	1.06	0.06	0.94	448	0.86	0.64	1.16	0.16	0.84	448	0.75	0.55	1.00	0.03	0.97
NIAID ≥6 Day 28	435	0.85	0.57	1.26	0.21	0.79	448	1.02	0.70	1.48	0.54	0.46	448	0.75	0.51	1.08	0.06	0.94
MSOFA=0 Day 7	436	1.14	0.95	1.39	0.92	0.08	448	1.19	0.99	1.44	0.97	0.03	448	1.08	0.89	1.31	0.77	0.23
MSOFA=0 Day 14	437	1.01	0.92	1.10	0.57	0.43	448	1.02	0.94	1.11	0.69	0.31	448	0.96	0.88	1.06	0.21	0.79
Vent-free survival Day 7	440	1.06	0.96	1.18	0.87	0.13	448	1.08	0.98	1.20	0.94	0.06	448	1.02	0.92	1.14	0.66	0.34
Vent-free survival Day 14	439	0.99	0.92	1.06	0.37	0.63	448	1.00	0.94	1.07	0.53	0.47	448	0.95	0.89	1.03	0.10	0.90
Vent-free survival Day 28	435	1.01	0.96	1.07	0.67	0.33	448	1.02	0.97	1.08	0.83	0.17	448	0.97	0.91	1.03	0.15	0.85
Death Day 7	442	0.05	0.00	0.97	0.02	0.98	448	1.01	0.19	5.34	0.50	0.50	448	0.03	0.00	0.45	0.00	1.00
Death Day 14	442	1.15	0.41	3.36	0.61	0.39	448	1.67	0.65	4.78	0.85	0.15	448	0.80	0.30	2.03	0.32	0.68
Death Day 28	437	1.01	0.56	1.83	0.52	0.48	448	1.33	0.77	2.33	0.85	0.15	448	1.33	0.77	2.33	0.85	0.15
Hypotension Day 7	266	0.90	0.38	2.06	0.40	0.60	448	9.94	5.83	18.99	1.00	0.00	448	0.09	0.05	0.17	0.00	1.00
Hypotension Day 14	119	1.65	0.82	3.45	0.92	0.08	448	18.32	10.69	36.01	1.00	0.00	448	0.08	0.05	0.13	0.00	1.00

Priors for Intercepts ~Normal(0,10) and all other coefficients ~Normal(0. 3.13) in the log-form.
Posterior probability (PP) of 1.00 really indicates PP > 0.999.
For crosstabs, N missing per group, model fit statistics, posterior predictive checks, see under Models section.
Models shown in this order: Unimputed, Analysis 1, Analysis 2.
