# Supplementary figures and images for: Identification of HIF1A as a therapeutic target during SARS-CoV-2–associated lung injury
Source: JCI Insight. 2025 Jun 17;10(14):e191463. doi: 10.1172/jci.insight.191463 (PMC12288972; doi:10.1172/jci.insight.191463)

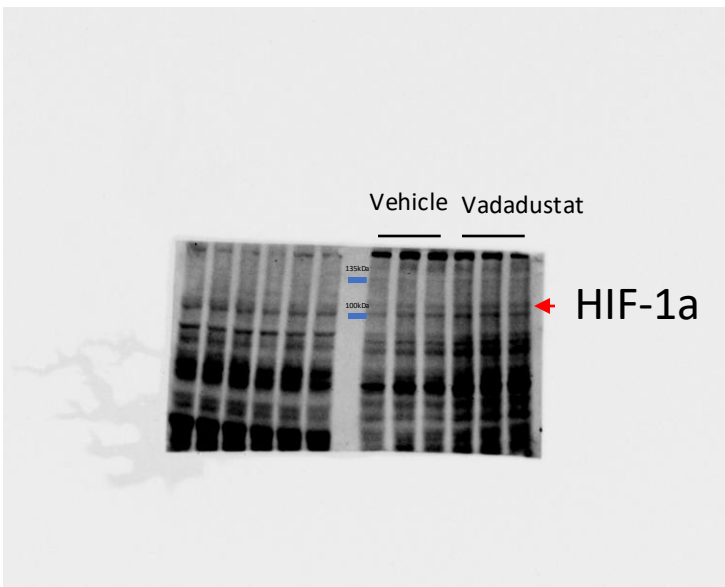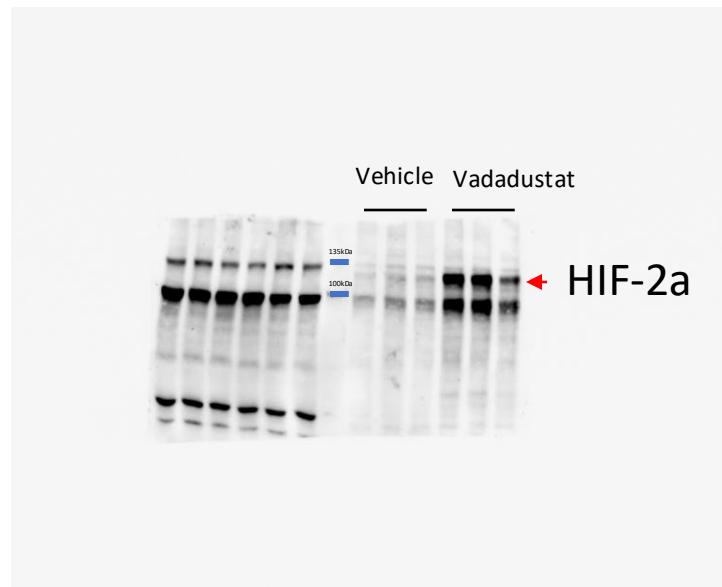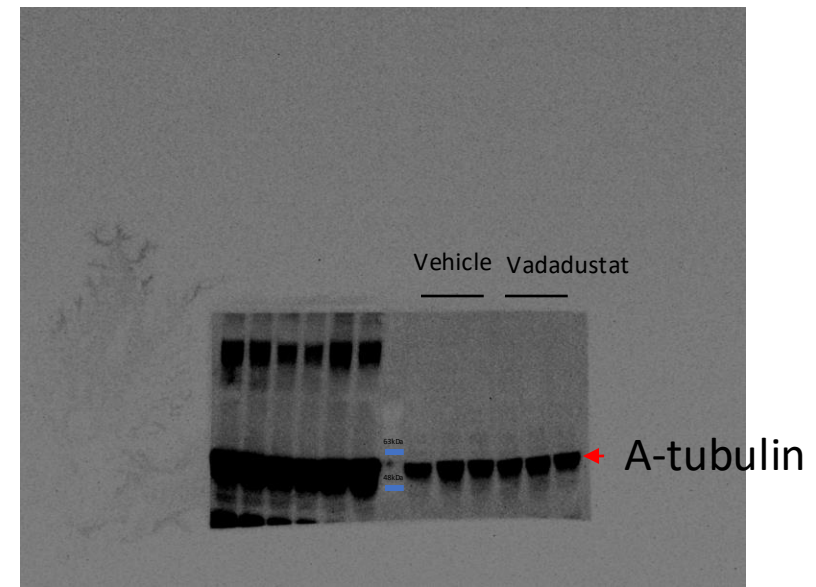

Supplement: Unedited blot and gel images [file jciinsight-10-191463-s222.pdf]
